# Three-Minute Enantioselective Amino Acid Analysis by Ultra-High-Performance Liquid Chromatography Drift Tube Ion Mobility-Mass Spectrometry Using a Chiral Core–Shell Tandem Column Approach

**DOI:** 10.1021/acs.analchem.3c05426

**Published:** 2024-02-01

**Authors:** Simon Jonas Jaag, Younes Valadbeigi, Tim Causon, Harald Gross, Michael Lämmerhofer

**Affiliations:** † Pharmaceutical (Bio-)Analysis, Institute of Pharmaceutical Sciences, University of Tuebingen, Auf der Morgenstelle 8, 72076 Tuebingen, Germany; ‡ Department of Chemistry, Faculty of Science, Imam Khomeini International University, Nowrouzian, 3414896818 Qazvin, Iran; § University of Natural Resources and Life Sciences, Vienna Department of Chemistry, Institute of Analytical Chemistry, Muthgasse 18, 1190 Vienna, Austria; ∥ Pharmaceutical Biology, Institute of Pharmaceutical Sciences, University of Tuebingen, Auf der Morgenstelle 8, 72076 Tuebingen, Germany

## Abstract

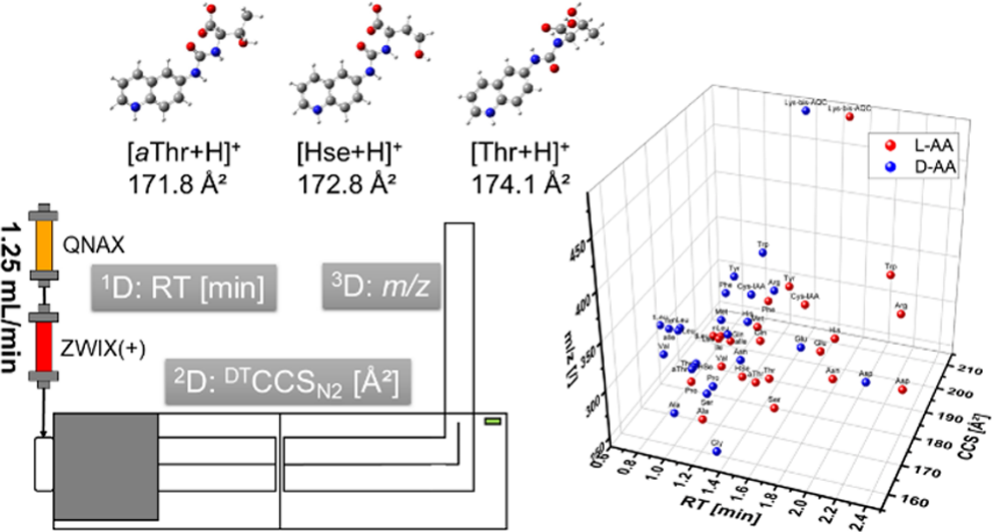

Fast liquid chromatography (LC) amino acid enantiomer separation of 6-aminoquinolyl-*N*-hydroxysuccinimidyl carbamate (AQC) derivatives using a chiral core–shell particle tandem column with weak anion exchange and zwitterionic-type quinine carbamate selectors in less than 3 min was achieved. Enantiomers of all AQC-derivatized proteinogenic amino acids and some isomeric ones (24 in total plus achiral glycine) were baseline separated (*R*_s_ > 1.5 except for glutamic acid with *R*_s_ = 1.3), while peaks of distinct amino acids and structural isomers (constitutional isomers and diastereomers of leucine and threonine) of the same configuration overlapped to various degrees. For this reason, drift tube ion mobility-mass spectrometry was added (i.e., LC-IM-MS) as an additional selectivity filter without extending run time. The IM separation dimension in combination with high-resolution demultiplexing enabled confirmation of threonine isomers (threonine, *allo*-threonine, homoserine), while leucine, isoleucine, and *allo*-isoleucine have almost identical collisional cross-section (*^DT^CCS_N2_*) values and added no selectivity to the partial LC separation. Density functional theory (DFT) calculations show that IM separation of threonine isomers was possible due to conformational stabilization by hydrogen bond formation between the hydroxyl side chain and the urea group. Generally, the CCS_N2_ of protonated ions increased uniformly with addition of the AQC label, while outliers could be explained by consideration of intramolecular interactions and additional structural analysis. Preliminary validation of the enantioselective LC-IM-MS method for quantitative analysis showed compliance of accuracy and precision with common limits in bioanalytical methods, and applicability to a natural lipopeptide and a therapeutic synthetic peptide could be demonstrated.

## Introduction

Fast enantioselective amino acid analysis has increasing importance in pharmaceutical sciences, biomedical research, and food science for the determination of d-amino acids in ribosomal or nonribosomal natural (lipo)peptides^1−4^ and synthetic therapeutic peptides (after hydrolysis),^5−7^ as biomarkers of diseases,^8,9^ and in enantioselective metabolomics.^10^ To this end, enantioselective liquid chromatography (LC) is the method of choice and can be performed either (i) directly without prior derivatization or after achiral derivatization on a chiral stationary phase (CSP) or (ii) indirectly after derivatization with a chiral derivatizing agent (CDA) and use of an achiral stationary phase (usually reversed-phase).^10^ For direct LC enantiomer separation of free amino acids, Crownpak CR-I^11^ (18-crown-6 with the 3,3′-bis-phenyl-2,2′-binaphthyl moiety replacing one ethylene bridge as a chiral selector), Chiralpak ZWIX(+)^12^ [with a (1″*S*,2″*S*)-transsulfocyclohexylcarbamoylquinine selector], and Chirobiotic T^13^ and TAG^14,15^ (with teicoplanin and teicoplanin aglycone selectors) and the corresponding core–shell teicoplanin column^16^ have shown broad enantioselectivity. For example, all free proteinogenic amino acids, except for Pro, have been directly resolved using Crownpak CR-I(+) column by high-performance liquid chromatography coupled with electrospray ionization tandem mass spectrometry (HPLC–ESI-MS)/MS in 5 min^11^ and supercritical fluid chromatography (SFC) in 3 min.^17^ With a teicoplanin core–shell column, fast enantiomer separations in time scales <1 min have been reported for proteinogenic and nonproteinogenic amino acids using LC-UV and injections of single amino acids, yet the simultaneous analysis of all amino acids in a single run has not been shown so far and poses additional challenges even with mass spectrometric (MS) detection.^18^ Achiral derivatization has been used to improve the enantioselectivity for CSPs, introduce a strong fluorophore or chromophore for spectroscopic detection, or a moiety with improved ionization efficiency for MS detection and (ideally) characteristic fragment ions for tandem MS (MS/MS) experiments.^19−26^ The resultant LC separations of complex derivatized amino acid mixtures have typically been on the 5–30 min time scale. To overcome limited chemoselectivity for separation of the challenging suite of Leu and Thr isomers, two-dimensional liquid chromatography (2D-LC) with achiral reversed-phase liquid chromatography (RP-LC) in the first dimension and a chiral column in the second dimension has been suggested but extends the total analysis time (typically >60 min).^22,23,25,27^ Very recently, a three-dimensional liquid chromatography (3D-LC) approach for enantioselective analysis of aliphatic amino acids in urine with RP in the first dimension, a mixed-mode column having remarkable selectivity for the structural isomers of Leu (Leu/Ile/*a*Ile) in the second dimension, and an enantioselective column in the third dimension was suggested.^28^

On the other hand, numerous indirect LC enantiomer separation methods have been reported in the literature (for a recent overview, see, e.g., ref (10)). They also recently experienced a renaissance in enantioselective metabolomics. However, all indirect methods must be employed with care, especially when relative quantification is applied for deriving enantiomer ratios directly from peak areas.^10^ Kinetic resolution due to incomplete derivatization, racemization, distinct detector response of the detected diastereomers (e.g., distinct ESI ionization efficiency, different fragmentation rates in tandem MS) and enantiomeric impurities in the CDA may easily introduce bias and need proper consideration by calibration and over the course of validation.^10^ Like in LC, these problems may also exist in IM-MS and can be alleviated by direct enantiomer separation methods.

Due to the low-throughput nature of LC enantiomer separation methods, the potential of ion mobility (IM) separation for addressing different types of isomers on a ms time scale has been explored in recent years.^29^ IM can separate isomeric ions in the gas phase based on their mobility differences observed under the influence of an electric field applied across a drift cell filled with inert buffer gas, such as nitrogen or helium. Importantly, IM separation provides some orthogonality to mass spectrometry that separates ions according to the mass-to-charge ratio (*m*/*z*), which is realized using hybrid IM-MS instrumentation. However, under typical IM-MS conditions, enantiomers exhibit identical mobilities and IM-derived collision cross sections (CCS), meaning that chiral auxiliaries are required to permit their separation.^30,31^ Indeed, several studies have shown proof-of-principle enantioselective amino acid analysis using IM-MS with additional chiral chelating agents (forming ternary metal complexes),^32^ cyclodextrins,^33^ or linear oligosaccharides^34^ as chiral sectors. Distinction of enantiomers in such cases is enabled via the formation of diastereomeric complexes or supramolecular assemblies with d- and l-amino acid enantiomers, which exhibit distinct mobilities and can therefore be separated via IM. On the other hand, a number of indirect approaches employing CDAs to form diastereomers prior to analysis by IM-MS have also been published and several have achieved enantiomer separations for a larger set of amino acids, e.g., trapped ion mobility-MS (TIM-MS) in combination with (+)-1-(9-fluorenyl)ethyl chloroformate (FLEC)^35,36^ and (*S*)-naproxen chloride, respectively, the latter incorporating a short SCX-based ion-exclusion sample preseparation with a total analysis time of 3 min.^37^ Progress has been made in recent studies making use of estradiol-3-benzoate-17β-chloroformate derivatization and analysis with U-shaped IM-QqQ-MS^38^ and use of *N*-(2,4-dinitro-5-fluorophenyl)-l-alaninamide (FDAA, Marfey’s reagent) with TIM-MS.^39^ Yet, resolution for several diastereomeric pairs was limited and IMS resolution at half height for most Leu isomers could not be achieved.^30^ For this reason, the goal was to develop a rapid, enantioselective amino acid analysis method with a 1–5 min analysis time scale. Fast LC enantiomer separations on a 1 min time scale have been accomplished on chiral superficially porous particle (SPP) columns recently.^40−44^ However, in such rapid LC enantiomer separations of a complex mixture of amino acids, many peak overlaps of distinct amino acids are observed due to limited chemoselectivity and insufficient peak capacity of CSPs in a 1 min gradient LC run. Mass spectrometric detection can provide selectivity to distinguish between the majority of the overlapped peaks, except for some isobaric and isomeric amino acids (structural isomers of Thr and Leu). On the contrary, it has been demonstrated that IM can resolve some constitutional amino acid isomers and diastereomers on the ms time scale.^30^

Hence, the current work focuses on the combination of fast enantioselective LC with SPP columns with IM-MS for selective analysis of AQC-derivatized proteinogenic amino acids (AQC-AAs), including their structural isomers (i.e., enantiomers, constitutional isomers, and diastereomers). Ion suppression effects resulting from LC coelutions due to co-ionizations in the ESI source should be compensated for by d- and l-uniformly ^13^C^15^N-labeled AQC-AA internal standards. It is the first time that direct LC enantiomer separation is coupled to IM-MS yielding comprehensive enantioselective amino acid analysis on a time scale of 1–5 min. In contrast to reported indirect IM-MS enantiomer separations, the presented new method does not suffer from problems, such as kinetic resolution, bias from enantiomeric impurities in CDA, and complications that arise from the detection of diastereomers (such as different detector responses and fragmentation rates in tandem MS), relevant especially when relative quantification is employed for determination of enantiomer ratios. The potential of liquid chromatography ion mobility-mass spectrometry (LC-IM-MS) for enantioselective amino acid profiling was evaluated by application to a nonribosomal lipopeptide and a synthetic therapeutic peptide, and the quantitative performance was elucidated as well.

## Experimental Section

Materials, sample preparation, derivatization, and calculation of chromatographic parameters can be found in the Supporting Information (Notes S1–S4, Tables S1–S3, and Figures S1–S4).

### Instrumentation

Evaluation of the single column and tandem column performances was done on an Agilent 1290 Infinity II LC-Instrument (Agilent Technologies, Waldbronn, Germany) with a binary pump (G4220A), thermostated column compartment (G1316A), hyphenated to an HTS PAL autosampler (CTC Analytics, Zwingen, Switzerland) and an API 4000 triple quadrupole mass spectrometer (Sciex, Darmstadt, Germany) controlled by Analyst 1.7 software (Sciex).

An Agilent 1290 Infinity II UHPLC system was used for the chromatographic separation with a tandem column consisting of a QN-AX^43^ (*tert*-butylcarbamoylquinine selector, Figure S5a) coupled in-line with a ZWIX(+)^44^ (with a (1″*S*,2″*S*)-transsulfocyclohexylcarbamoylquinine selector, Figure S5b) core–shell column (both 2.7 μm particle diameter, 160 Å and 50 × 3 mm column dimension connected through a short stainless steel capillary, 0.12 × 75 mm). The injection volume was 2 μL and the column temperature was 50 °C. Drift tube ion mobility-mass spectrometry (DTIM-MS) measurements were made using an Agilent 6560 IM-QTOFMS equipped with a Dual AJS ESI Ion Source (Agilent Technologies) and controlled using MassHunter Acquisition software. The following source conditions were used: drying gas flow of 8 L/min at 275 °C, sheath gas flow of 12 L/min at 350 °C, nebulizer gas pressure of 30 psi, capillary voltage of 3500 V, and nozzle voltage of 500 V. Reference masses (purine and HP-921) were constantly infused into the second nebulizer to ensure accurate mass determination.

Using the 4-bit multiplexed operation mode with 8 packages/frame in a pseudorandom Hadamard-type sequence to improve analytical performance,^45^ a trap filling time of 1250 μs, a trap release time of 150 μs, a maximum drift (arrival) time of 50 ms, and a total of 5 IM transients were summed into each data frame. The TOFMS was operated in the 1700 extended dynamic range mode (2 GHz) and was tuned and calibrated using the vendor-recommended ESI-L tune mix (G1969-85000 and 0.1 mmol/L HP-0321 from Agilent Biopolymer Reference Kit, Agilent Technologies). Additional external calibration for *^DT^CCS_N2_* determination followed previously elaborated protocols using the same tune mix ions as calibrants.^46^

### Data Analysis

LC-DTIM-TOFMS data were preprocessed using a combination of vendor and open-source tools. All datafiles were demultiplexed using the PNNL Preprocessor (2021.04.21) and mass recalibrated using the IM-MS Reprocessor (Agilent Technologies). *^DT^CCS_N2_* determination was performed using the IM-MS Browser 10.0 (Agilent Technologies) to determine and subsequently apply the linear regression coefficients to calibrate all datafiles.^46^ Additional high-resolution demultiplexing (HRdm)^47^ of selected examples was also performed (Supporting Information, Note S5 for all preprocessing settings for standard and high-resolution demultiplexing workflows). Skyline 22.2 was used for targeted data evaluation and extraction of XICs, including IM-filtered examples. Origin Pro 2022 was used to generate graphs, and Microsoft Excel 2019 was used for calculation.

### Computational Methods

Structures of all conformers of protonated AQC-derivatized amino acids were fully optimized by density functional theory (DFT) with the ωB97xD functional. The basis set 6-311++G(d,p), including both diffuse and polarization functions, was used for the calculations. Frequency calculations were performed at the same level of theory at 298.15 K to find optimized structures for the local minima. Charge distribution was calculated by using the Merz–Kollman (MK) method. Gaussian 16 software was used for the DFT calculations.^48^ The Gaussian output files containing geometrical parameters of the optimized structures and MK charges were used to build input files for CCS_N2_ calculations. CCS_N2_ calculations were performed using MOBCAL-MPI software using the trajectory method (TM) at 298 K.^49^

## Results and Discussion

### Separation of Enantiomers by Single and Tandem Columns

LC conditions were optimized with the goal of achieving both a short (<5 min) analysis time and simultaneously separating all proteinogenic AQC-AAs in one run. To this end, two different chiral columns and their combination in a tandem arrangement were evaluated.^27,50^ The resolution results (Table 1) show that baseline separation (*R*_s_ ≥ 1.5) for 22 of 24 chiral AQC-AA enantiomer pairs can be achieved using the single QN-AX column with only Arg (*R*_s_ = 0.98, Figure S6a) and Asp (*R*_s_ = 0.79, Figure S6b) not fully separated. The basic Arg exhibits weak retention on the QN-AX column, which can be explained by repulsive effects between positively charged Arg side chain and the positively charged quinuclidine moiety of the stationary phase. Conversely, the basic amino acids His (*R*_s_ = 3.25) and Lys (*R*_s_ = 4.13) can be resolved well by the QN-AX column, which can be rationalized by the lower basicity of the His side chain (p*K*_a_ = 6.04) compared to the Arg residue (p*K*_a_ = 12.10) and bis-AQC derivatization of Lys, which leads to a neutral side chain without repulsive electrostatic effects. The low resolution of Asp enantiomers on the QN-AX column is mainly a result of the dominating ionic interactions of α- and side-chain carboxylate groups with the anion-exchange site of the stationary phase, which occur mostly nonstereoselectively, i.e., are largely of equal strength for both enantiomers leading to low enantioselectivity. Stereoselective interactions of other functional groups, such as the urea of AQC-Asp and carbamate group of the chiral selector, seem to have limited relative strength and influence compared to the anion-exchange interactions.

**Table 1:** Resolution (*R*_s_) Values of Enantiomer Pairs Using the Single QN-AX, ZWIX(+), or the Combined Tandem Columns^a^

AQC-AA	QN-AX	ZWIX(+)	tandem
*a*Ile	3.30	0.00	4.27
Ala	1.97	0.44	2.75
Arg	0.98	6.49	7.67
Asn	7.08	3.86	7.08
Asp	0.79	2.36	2.11
*a*Thr	4.06	2.79	3.70
Cys-IAA	3.93	2.88	4.60
Gln	2.60	1.84	2.83
Glu	1.92	1.31	1.27
His	3.25	4.17	5.72
Hse	3.30	2.36	2.83
Ile	3.30	0.00	4.08
Leu	2.56	0.00	2.29
Lys-bis-AQC	4.13	1.93	3.78
Met	3.67	0.98	3.98
*n*Leu	3.41	0.00	4.06
Phe	5.02	1.57	3.78
Pro	1.70	0.00	1.89
Ser	4.33	4.60	5.78
Thr	5.19	2.79	3.75
*t*Leu	4.87	0.00	4.03
Trp	4.72	8.58	9.87
Tyr	4.98	2.70	5.38
Val	6.74	1.18	5.77

a Each 2.7 μm particle diameter, 160 Å and 50 mm × 3 mm column dimension. Mobile phase A: 10 mM NH_4_FA and 10 mM FA in ACN/MeOH/H_2_O (49:49:2; v/v/v), mobile phase B: 50 mM NH_4_FA and 50 mM FA in ACN/MeOH/H_2_O (49:49:2; v/v/v); flow rate: 1.25 mL/min, gradient: 0–0.4 min 0% B, 0.4–1.0 min 0–100% B, 1.0–3.0 min 100% B, 3.0–3.2 min 100–0% B, 3.2–4.0 min 0% B; column temperature: 50 °C.

On the ZWIX(+) column, the guanidinium group of Arg experiences attractive electrostatic interactions with the sulfonate moiety of the ZWIX(+) selector; hence, Arg enantiomers are well retained (Figure S6a). On the contrary, the carboxylate groups of Asp experience a repulsive electrostatic effect on ZWIX(+) due to the sulfonate moiety of the chiral selector and hence show less retention. The stereoselective urea–carbamate hydrogen bonding interaction gains relative importance, affording good enantioselectivity (Figure S6b). On the ZWIX(+) column, Arg and Asp were favorably separated, however, only 14 of 24 enantiomer pairs yielded *R*_s_ > 1.5 under the given conditions with insufficient resolution observed for the aliphatic, hydrophobic amino acids, such as Leu isomers, Ala, and Val, probably due to their weak retention with the employed polar organic elution mode (Table S4, hydrophobic interactions do not play a major role under these highly organic elution conditions).

**Figure 1:**
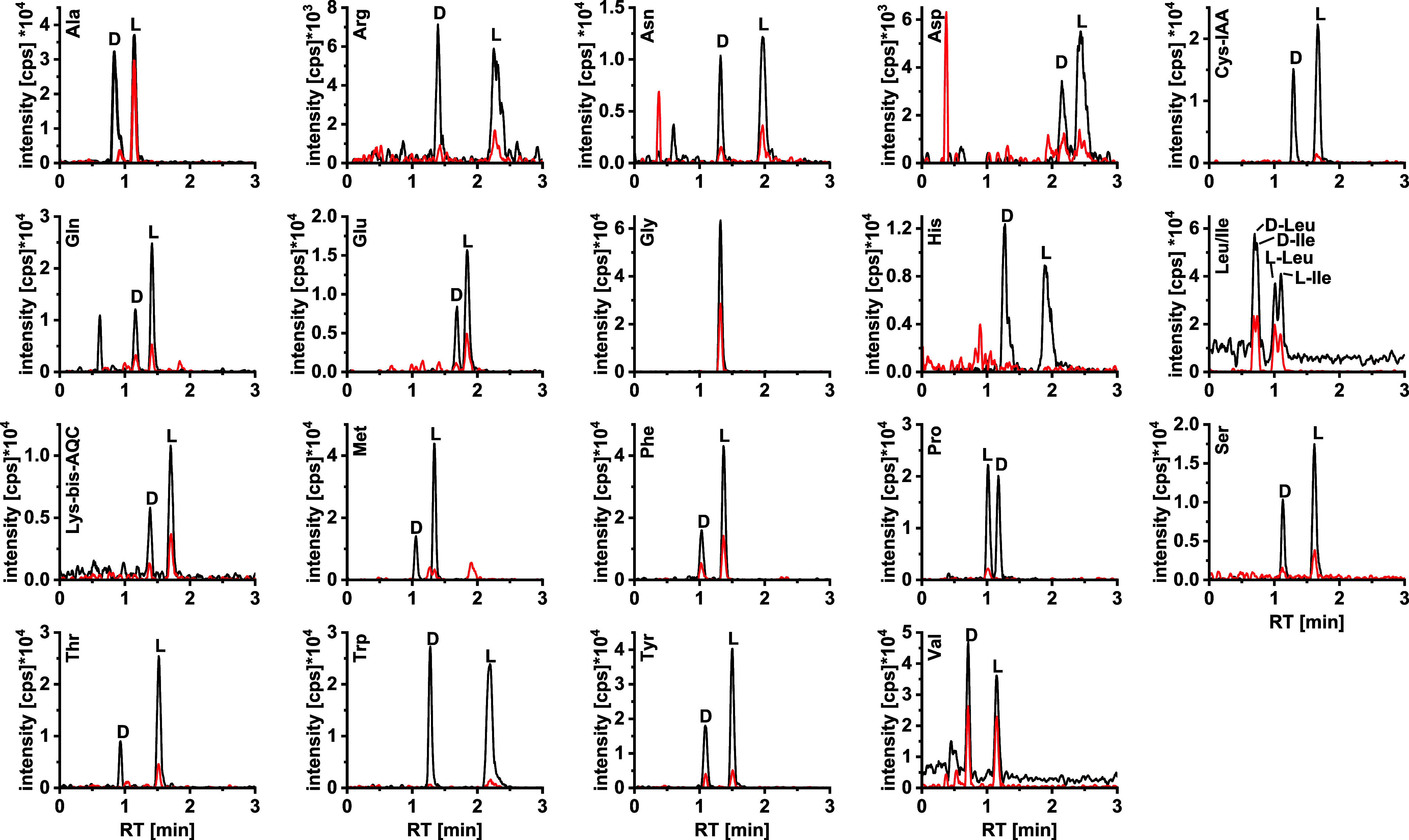
Extracted ion chromatograms (EICs) (intensity vs LC retention times) of the 20 AQC-derivatized proteinogenic amino acids (black trace) and their respective U^-13^C^15^N standards (red trace). Tandem column: QN-AX coupled in-line with ZWIX(+) prototype core–shell columns (3.0 × 50 mm, 2.7 μm, respectively). Mobile phase A: 10 mM NH_4_FA and 10 mM FA in ACN/MeOH/H_2_O (49:49:2; v/v/v); mobile phase B: 50 mM NH_4_FA and 50 mM FA in ACN/MeOH/H_2_O (49:49:2; v/v/v); flow rate: 1.25 mL/min; gradient: 0–0.4 min 0% B, 0.4–1.0 min 0–100% B, 1.0–3.0 min 100% B, 3.0–3.2 min 100–0% B, 3.2–4.0 min 0% B; column temperature: 50 °C.

Based on this observation, a combination of the two columns in a tandem arrangement was evaluated. The separation factors achieved on the tandem column (QN-AX coupled in series with ZWIX(+) as the second column in the tandem approach; both with d-enantiomer eluting before the l-enantiomer except for Pro for which the elution order is reversed on both columns) are due to a combined additive retention effect from both columns:^51^ Arg and Asp benefitted from the enantioselectivity of the ZWIX(+) column (Figure S6a,b) while maintaining high resolution for the other amino acids, e.g., Asn like other amino acids was fully baseline separated on both columns (Figure S6c). Pro enantiomers were insufficiently retained and not resolved on ZWIX(+) under given conditions; however, QN-AX contributed sufficient enantioselectivity (Figure S6d). With this arrangement, only the resolution for Glu was substantially reduced compared to the single QN-AX column (*R*_s_ = 1.92 vs 1.27) and it was the only AQC-AA with *R*_s_ < 1.5 using the tandem column setup. While most AQC-derivatized enantiomers could be separated within 1 min using the tandem column approach, important constitutional isomers and diastereomers coelute under such fast LC elution regime and therefore need specific attention, which was partly achieved by extending the separation to 3 min. In total, 23 of 24 AQC-AA enantiomer pairs can be resolved with a resolution ≥1.5, while Glu is partially resolved (*R*_s_ = 1.27) within a 3 min analysis time plus re-equilibration (Figure 1).

### LC Separation of Isomeric Amino Acids

In addition to the separation of enantiomers, the tandem column method was evaluated for the separation of Leu isomers (Leu, Ile, *a*Ile, *t*Leu, *n*Leu) and Thr isomers (Thr, *a*Thr, Hse) of identical configurations. This is of critical importance since these isomers cannot be distinguished by MS due to their identical precursor ion *m*/*z* and lack of diagnostic fragment ions in tandem MS experiments for AQC-AA derivatives.

### Leu Isomers

**Figure 2:**
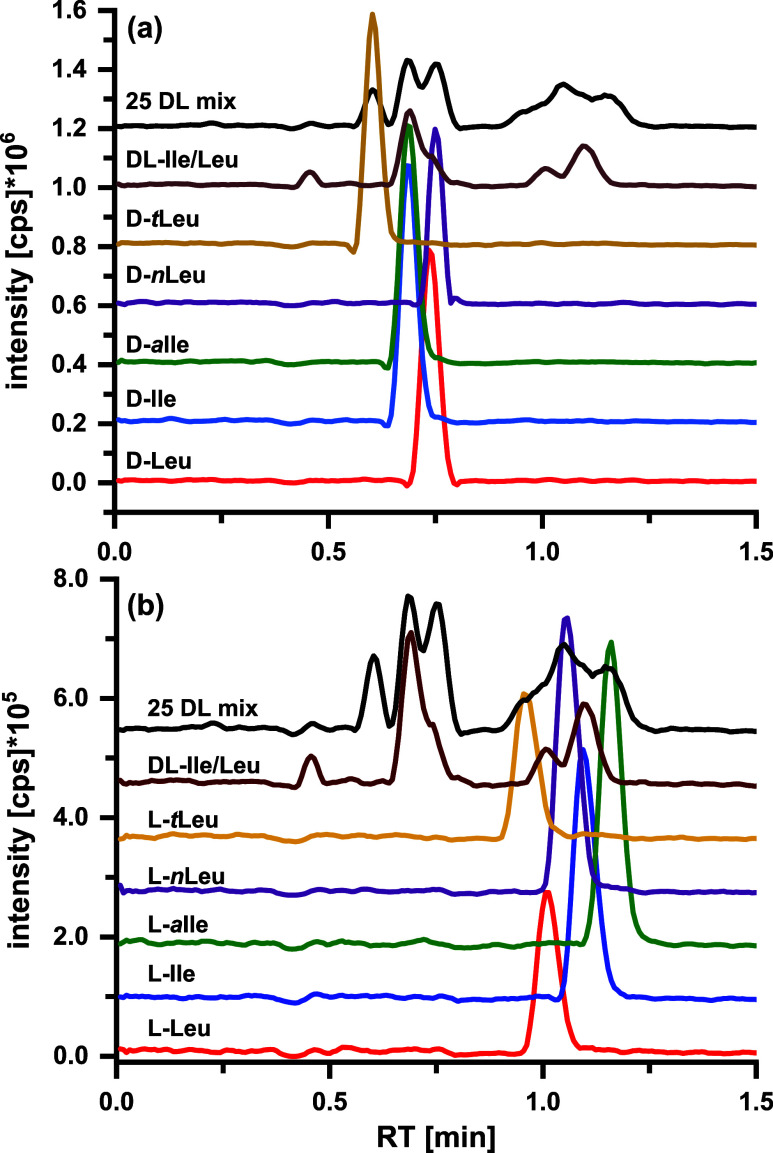
Extracted ion chromatograms (EICs) (intensity vs LC retention times) of the AQC–leucine isomers. (a) d-leucine isomers and (b) l-leucine isomers. Experimental conditions are the same as those described in Figure 1.

Enantiomers of the investigated amino acids can be resolved within 1 min after the AQC derivatization on the employed tandem column. However, under such fast elution regime, constitutional isomers and diastereomers coelute. They need specific attention, which was partly achieved by extending the analysis time. Using optimized conditions on the tandem column, d-*t*Leu elutes first and can be separated with a resolution of 1.18 from the coeluting pair d-*a*Ile/d-Ile (Figure 2a and Table S5). Conversely, a partial separation between the biologically important isomer pairs d-Ile and d-Leu (*R*_s_ = 0.75) can be achieved. There was no separation between d-Leu and the noncanonical d-*n*Leu in the mixture even though a slight difference in the retention time was observed for the single standard injections. However, *n*Leu is usually not present in biological and pharmaceutical samples. The important pairs l-Leu and l-Ile (*R*_s_ = 0.79, Table S6) can be partially separated if the other Leu isomers are not present. l-Ile and l-*a*Ile (*R*_s_ = 0.88) are partially separated, but only a minor degree of separation of l-*t*Leu from l-Leu (*R*_s_ = 0.42) is observed (Figure 2b). Nevertheless, this level of resolution of Leu isomers could provide enough selectivity between the biologically relevant l-Ile and l-Leu, while their d-enantiomers can be determined as their sum in the initial screening approach.

### Thr/*a*Thr/Hse

**Figure 3:**
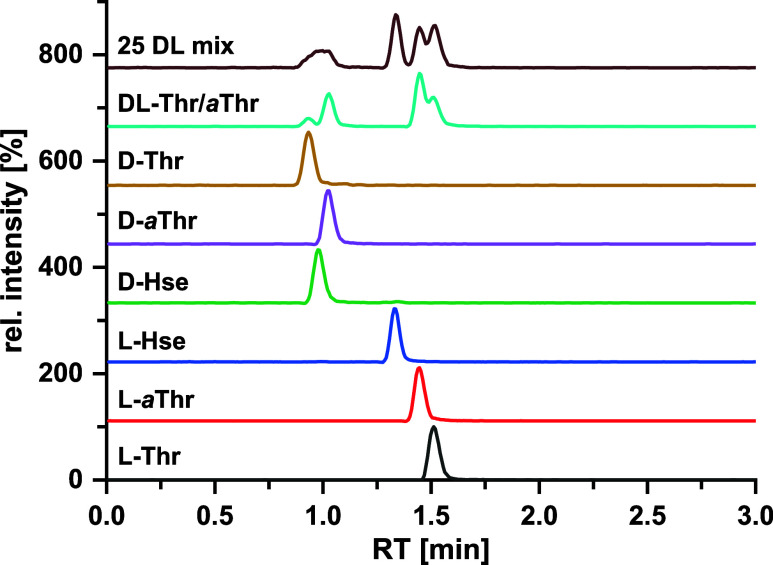
Extracted ion chromatograms (EICs) (intensity vs LC retention times) of the AQC–threonine isomers. Tandem column: QN-AX + ZWIX(+) prototype core–shell columns (3.0 × 50 mm, 2.7 μm, respectively). Experimental conditions are the same as described in Figure 1.

The separation of the AQC-Thr isomers is shown in Figure 3. In the 25 dl-mixture, the d-enantiomers of Thr, *a*Thr, and Hse coelute as a single broad peak. l-Hse can be baseline separated from l-*a*Thr (*R*_s_ = 2.83, Table S7), but only partial separation between l-*a*Thr and l-Thr (*R*_s_ = 0.83) is observed. However, biological samples typically do not contain Hse and hence only the separation of a mixture of Thr/*a*Thr is relevant for the majority of cases, whereby near-baseline separation is achieved (i.e., d-Thr and d-*a*Thr: *R*_s_ = 1.18; Table S8).

### Evaluation of IM-MS to Improve the Fast Enantioselective Method

**Figure 4:**
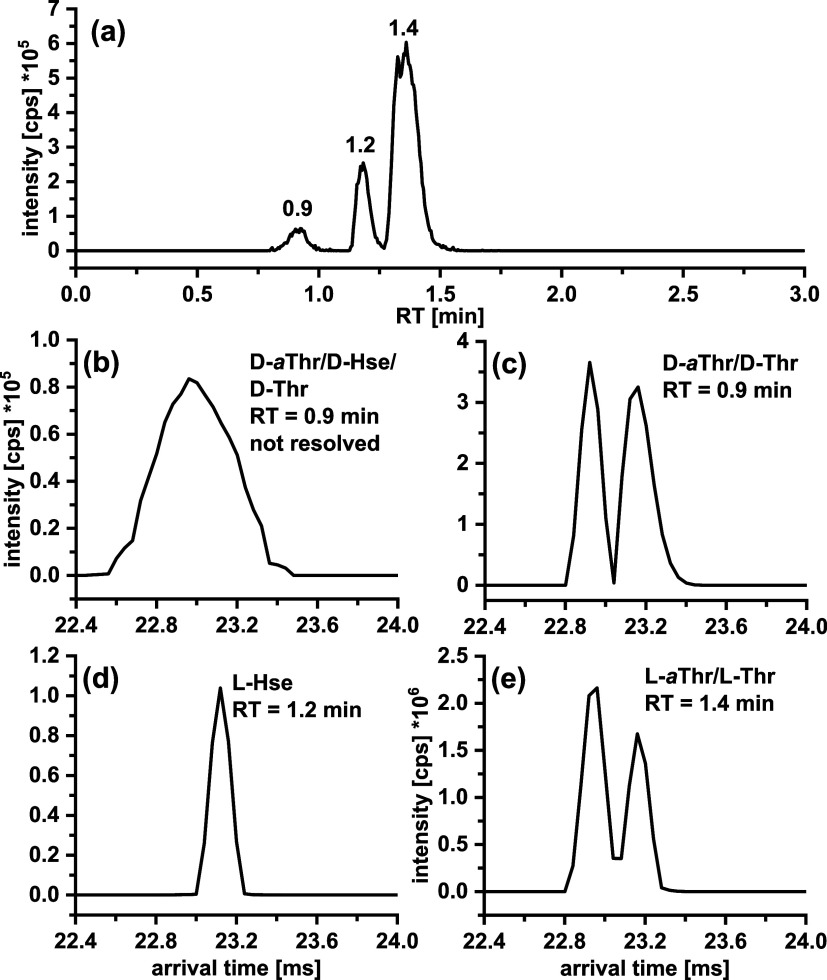
(a) Extracted ion chromatogram (EIC) of a mixture of three AQC–threonine isomers and (b–e) corresponding arrival time spectra after high-resolution demultiplexing (HRdm) of the respective AQC–threonine isomers. Panels (b, d, e) show the arrival time spectra of a sample containing dl-Hse, while panel (c) shows a spectra of a sample without dl-Hse where in comparison to (b) d-*a*Thr and d-Thr can be separated. Extraction of the spectra was performed at 302.150 *m*/*z* ± 15 mDa.

The aforementioned shortcomings of enantioselective LC for separating constitutional isomers and diastereomers under fast elution conditions raised the question of whether results could be improved using an additional IM separation dimension integrated without extending the total analysis time. First, the gain in resolving power between standard arrival time spectra with broad IM peaks (Figure S7a) and HRdm spectra (Figure S7b) with much sharper peaks with the same arrival times and, consequently, ^*DT*^*CCS*_*N2*_ values can be clearly demonstrated as has already been shown for other analytes.^45,47,52^ Across the full set of amino acids evaluated in this work, the addition of the AQC derivatization led to a systematic increase in the observed *^DT^CCS_N2_* values of the corresponding protonated molecular ions (Figure S8). This broad trend is unsurprising due to the consistent protonation site on the heteroatom of the aminoquinoline group and the structural rigidity of the AQC group itself, which is also in good agreement with observations from previous work focused on dansylation of amino acids.^53^ However, some outliers within this trend were observed, including Trp and Tyr, which exhibit a much smaller increase in *^DT^CCS_N2_* because of the structural compression due to π–π interactions between the aromatic rings of the amino acid and AQC (Figure S9 and Table S9), while Val has a large shift in comparison to the structurally similar Thr. Val and Thr have large differences in their *^DT^CCS_N2_* values for the underivatized forms due to the contribution of the terminal OH group of Thr (Figure S10). In the case of the key isomer sets, Leu, Ile, and *a*Ile have almost identical ^*DT*^*CCS*_*N2*_ values (180.9 Å^2^), while *n*Leu is slightly larger (182.1 Å^2^) with a Δ^*DT*^*CCS*_*N2*_ of only 0.66% (Table 2, Figure S11, and Table S10). An intermediate ^*DT*^*CCS*_*N2*_ value was obtained for *t*Leu (181.6 Å^2^), leading to a Δ^*DT*^*CCS*_*N2*_ of only 0.39% compared to Leu, Ile, *a*Ile and 0.28% compared to *n*Leu and unsatisfactory results with HRdm data processing (Table S10 and Figure S11). Compared to the underivatized analogues (i.e., [AA-AQC+H]^+^ vs [AA+H]^+^), the selectivity for *t*Leu vs *n*Leu is reduced from 2.26 to 0.28% following AQC derivatization, while Leu vs Ile selectivity is also substantially reduced almost to no selectivity from 0.61 to <0.1%. These results can be rationalized by the free rotation of alkyl chains, leading to an averaged ^*DT*^*CCS*_*N2*_ for these isomers. In contrast, the combination of IM separation and application of HRdm allows separation of *a*Thr from Thr to be achieved for both d- and l-forms (Figure 4) and confirms the correct assignment of three l-isomers (*a*Thr, Hse, and Thr), while only the presence of all three d-isomers (Hse, *a*Thr, Thr) in samples could not be correctly assigned on a routine basis (representing the limitations of HRdm) and would require confirmatory measurements when inconclusive results are obtained. These results are possible due to the larger differences of the ^*DT*^*CCS*_*N2*_ values with Δ^*DT*^*CCS*_*N2*_ (*a*Thr, Hse) = 0.58%, Δ^*DT*^*CCS*_*N2*_ (*a*Thr, Thr) = 1.34%, and Δ^*DT*^*CCS*_*N2*_ (Hse, Thr) = 0.75%. In this case, the higher degree of separation achieved is due to the impact of the OH group on the alkyl chain and the formation of a hydrogen bond with the HNCONH of the AQC group preventing free rotation of the alkyl chain of the Thr isomers (Figures S10, S12 and S13; Tables S11–13). Optimized structures of different conformers of Leu isomers in the gas phase and their calculated *CCS*_*N2*_ values can be found in Figures S14–S16 and Tables S14–S16. Corresponding information for some uncommon AQC-AAs, sometimes present in nonribosomal peptides, are summarized in Figure S17 and Tables S17–19.

**Table 2:** Overview of the Chromatographic, Ion Mobility, and Mass Spectrometry Results of the Enantioselective Analysis with the QNAX-ZWIX(+) Tandem Column^a^

AQC-AA	RT(d) [min]	RT(l) [min]	*R*_s_	*^DT^CCS_N2_* [Å^2^]	*t*_A_ [ms]	*m*/*z*
*a*Ile	0.68	1.15	4.27	180.9	24.19	302.150
Ala	0.94	1.15	2.75	164.7	21.89	260.103
Arg	1.39	2.30	7.67	188.3	25.31	345.167
Asn	1.31	1.97	7.08	174.3	23.32	303.109
Asp	2.19	2.44	2.11	175.2	23.44	304.093
*a*Thr	0.98	1.45	3.70	171.8	22.94	290.114
Cys^b^	1.28	1.67	4.60	183.5	24.68	349.097
Gln	1.17	1.41	2.83	178.0	23.85	317.124
Glu	1.70	1.84	1.27	178.2	23.88	318.108
Gly^c^	1.32	n/a	n/a	159.5	21.15	246.087
His	1.28	1.91	5.72	181.1	24.29	326.125
Hse	0.98	1.34	2.83	172.8	23.07	290.114
Ile	0.68	1.06	4.08	180.9	24.19	302.150
Leu	0.75	1.06	2.29	180.9	24.19	302.150
Lys^d^	1.38	1.70	3.78	209.4	28.44	487.209
Met	1.07	1.34	3.98	182.0	24.39	320.106
*n*Leu	0.75	1.06	4.06	182.1	24.35	302.150
Phe	1.04	1.36	3.78	187.1	25.12	336.134
Pro	1.18	1.02	1.89	168.6	22.50	286.119
Ser	1.13	1.62	5.78	168.8	22.49	276.098
Thr	0.98	1.52	3.75	174.1	23.25	290.114
*t*Leu	0.60	1.01	4.03	181.6	24.28	302.150
Trp	1.27	2.19	9.87	191.4	25.80	375.145
Tyr	1.09	1.50	5.38	188.4	25.34	352.129
Val	0.71	1.15	5.77	175.6	23.44	288.134

a Each 50 mm × 3 mm column dimension packed with 2.7 μm core–shell particles. The exact (calcd)*m*/*z* value of the protonated species is provided. Mobile phase A: 10 mM NH_4_FA and 10 mM FA in ACN/MeOH/H_2_O (49:49:2; v/v/v); mobile phase B: 50 mM NH_4_FA and 50 mM FA in ACN/MeOH/H_2_O (49:49:2; v/v/v); flow rate: 1.25 mL/min; gradient: 0–0.4 min 0% B, 0.4–1.0 min 0–100% B, 1.0–3.0 min 100% B, 3.0–3.2 min 100–0% B, 3.2–4.0 min 0% B; column temperature: 50°C. *t*_A_ is the arrival time. A void time of 0.44 min was determined by injection of 1,3,5-tri-*tert*-butylbenzene as a void marker.

b Cys in its AQC-Cys-IAA form.

c Gly is an achiral amino acid.

d Lys in its bis-AQC-Lys form.

### Analysis of Natural Lipopeptides

**Figure 5:**
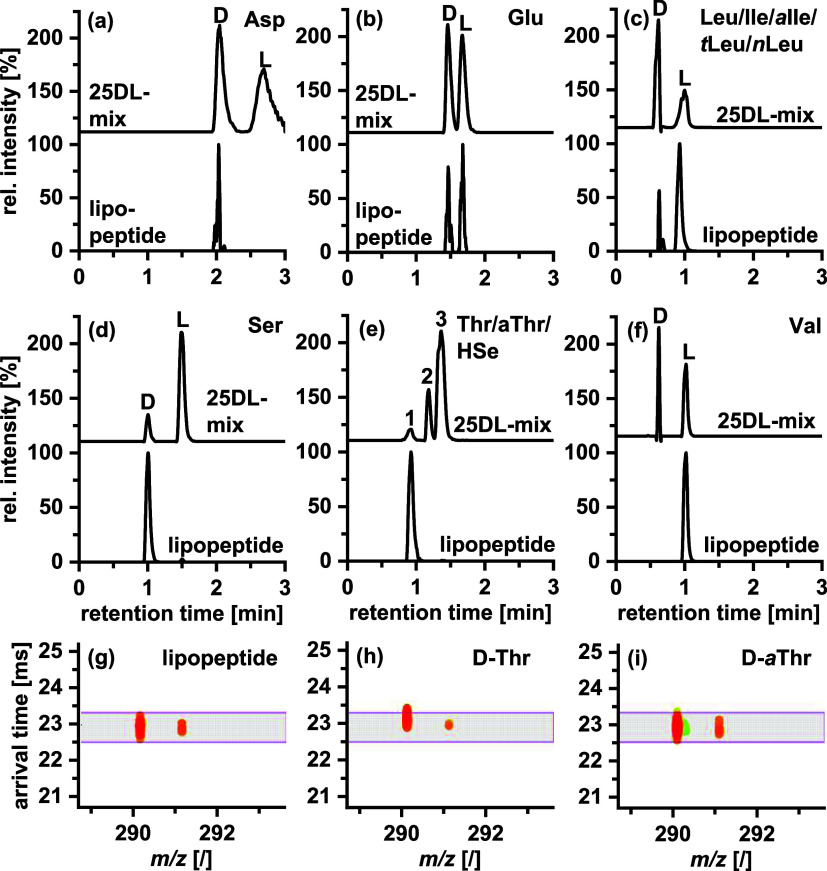
LC-IM-MS analysis of the lipopeptide hydrolysate. (a–f) Extracted ion chromatograms (EICs) of the hydrolysate and 2D contour plots of the (g) lipopeptide hydrolysate, (h) d-Thr standard, and (i) d-*a*Thr standard. The purple area indicates the IM-filtering range for d-*a*Thr. Peak assignment in (e): (1) d-Thr/d-aThr/d-Hse, (2) l-Hse, and (3) l-*a*Thr/l-Thr. Tandem column: QN-AX + ZWIX(+) prototype core–shell columns (3.0 × 50 mm, 2.7 μm, respectively). Mobile phase A: 10 mM NH_4_FA and 10 mM FA in ACN/MeOH/H_2_O (49:49:2; v/v/v); mobile phase B: 50 mM NH_4_FA and 50 mM FA in ACN/MeOH/H_2_O (49:49:2; v/v/v); flow rate: 1.25 mL/min, gradient: 0–0.7 min 0–100% B, 0.7–1.66 min 100% B, 1.66–1.7 min 100–0% B, 1.7–3.0 min 0% B; column temperature: 50 °C.

The LC-IM-MS method was subsequently employed to analyze a nonribosomal lipopeptide. Nonribosomal lipopeptides often contain any of Thr/*a*Thr/Hse as well as any of Leu/Ile/*a*Ile residues. From prior investigations, which were mainly based on NMR and bioinformatic studies (data not shown), partial information about the undecapeptide portion of the lipopeptide of interest was available. It possessed the following amino acid sequence: Leu1-Asp2-Thr3-Leu4-Leu5-Ser6-l-Leu7-Gln8-l-Leu9-l-Val10-l-Glu11. However, until this point, the absolute configurations of only 4 out of 11 amino acids were clarified. We now probed if our method can, upon full hydrolysis, confirm the amino acid composition and determine the absolute configurations of the remaining seven amino acids. The results (Figure 5, Tables 3, and S20) demonstrate the capability of the method to assign the absolute configuration of the incorporated amino acids. It can be readily derived from the chromatograms that Asp2 and Ser6 (Figure 5a+d) are present in their d-configuration and Val as the l-enantiomer (Figure 5f). For Glu, a 1:1 mixture of d- and l-enantiomers was determined (Figure 5b). Before hydrolysis, the lipopeptide contained one Gln and one Glu residue. In the course of peptide hydrolysis, the amide side chain of Gln is hydrolyzed (deamidation), leading to two equivalents of Glu (note: the 1:1 ratio is not due to racemization of Glu as evidenced by the absence of racemization of IS, and as also shown previously for another lipopeptide).^27^ This means that either Gln or Glu in the lipopeptide must have a d-configuration and the other a l-configuration. To our delight, the NRPS-based biosynthetic gene cluster of the investigated lipopeptide was known (Figure S20).^54^ In this context, the absolute configuration is determined either by the presence of an epimerization (E) or of a dual epimerization/condensation (C/E) domain.^55^ They are always localized downstream of the concerned NRPS module and are able to convert l-configured amino acids into their corresponding d-configuration. Since solely the Gln-module 8 was followed up by a C/E domain (Figure S20), Gln8 could be readily assigned bioinformatically as d-Gln8. Consequently, Glu11 had to be l-configured. Concerning the Thr isomers, the configuration could be assigned as the d-enantiomer. However, as LC–MS results alone could not elucidate which of the d-Thr, d-*a*Thr, or d-Hse isomers were present, the IM data were used as a second criterion for confirmation. Hse can be present in lipopeptides from *Pseudomonas* sp. The arrival time for the amino acid building block in the lipopeptide did not match with that of Hse (see Figures S21 and S22). It confirms previous complementary information from bioinformatics and NMR: Hse could be ruled out for bioinformatic reasons since A domains for Hse differ significantly from those recognizing Thr. Second, the analysis of the spin systems of each amino acid of the compound, employing an HSQC-TOCSY 2D-NMR spectrum, readily unveiled the presence of a Thr residue. Figure 5h,i shows the results for individual standards of Thr and *a*Thr with different arrival times in the IM dimension (23.25 ms for Thr and 22.94 ms for *a*Thr). Comparison with the arrival times in the sample (22.94 ms; Figure 5g) confirms the identity as *a*Thr. With an excellent match to the LC retention time (0.92 min) and not to that of d-Thr (RT = 0.85 ± 0.012 min), we could unequivocally assign this amino acid by enantioselective LC-IM-MS as d-*a*Thr3 (Figure S22).

**Table 3:** Amino Acid Composition and Determined Configuration of the Natural Lipopeptide

amino acid	Asp	Thr	Ser	Leu	Gln	Val	Glu
number	1	1	1	5	1	1	1
configuration	d	d-*a*Thr	d	n/a^a^	d^b^	l	l^b^

a Multiple Leu present with both d- and l-configurations.

b Gln is deamidated through the hydrolysis conditions.

In attempts to assign the absolute configuration of the five Leu isomers (Leu1/4/5/7/9) in the lipopeptide sample, overlays of sample and reference standard LC chromatograms (Figure S23a,b for corresponding d- and l-AQC-AAs) were used. Comparison of the retention time of the l-enantiomer peak in the sample with the individual l-AQC-AA standards of the other isomers reveals a good match with l-Leu, while there is a significant mismatch with the other isomers, including l-Ile and l-*a*Ile (Figures S23b and S24). Likewise, the comparison with d-enantiomer standards reveals the best agreement with d-Leu, but this result has to be taken with care due to the very minor retention time differences with d-Ile and d-*a*Ile and the insignificant differences in IM arrival times for this suite of isomers. Confirmation of the absolute configuration would need further verification, e.g., enantioselective 2D-LC^27^ or accepting longer run times with the current method. For this case, the correct assignment was made using NMR data, which suggested Leu as an amino acid residue in the lipopeptide. An l:d-enantiomer ratio of 4:1 can be derived from the peak area ratio in Figure 5c. The position of the single d-Leu amino acid in the lipopeptide remains open and needs further complementary investigation. However, this is beyond the scope of the present study.

The somatostatin-mimicking therapeutic peptide octreotide (Figure S25a) was also analyzed with absolute configurations of the amino acids in this peptide, agreeing with the results of the specifications (Figure S25b–g and Table S21). In this case, for Cys-IAA-AQC (Figure S25b) and Lys-bis-AQC (Figure S25c), the l-enantiomer was found. Two equiv of Phe were confirmed and could be assigned as one d- and one l-enantiomer (Figure S25d). In the case of the Thr isomers, either l-*a*Thr or l-Thr was suggested based on LC retention time (Figure S25e). IMS data (Figure S25h–j) together with LC retention time allow unequivocal identification as l-Thr because RT and arrival time (1.38 min, 23.25 ms, respectively) in the octreotide hydrolysate sample match perfectly with the l-Thr reference (1.38 min, 23.25 ms) but not l-*a*Thr (1.33 min, 22.94 ms). This indicates that in critical cases, IM-MS can help to reliably determine amino acid compositions in peptides. Another example, aureobasidin A, a cyclic depsipeptide antibiotic, is given in Table S22.

### Quantitative Method Performance

As the absolute concentrations of individual amino acids can vary greatly in real samples, the quantitative capabilities of the enantioselective LC-IM-MS method were evaluated using an 8-level calibration (Table S3). To this end, an external calibration with stable isotope-labeled internal standards (SIL-IS) was used. The molar concentrations of the SIL-IS mixture range from 12.1 mol % for l-Ala to only 0.4 mol % for l-His (Table S2). Corresponding d-amino acid SIL-IS were prepared by a racemisation procedure described elsewhere.^24^ Due to the varying amounts in the employed U-^13^C^15^N-labeled cell extract, the SIL-IS signal intensities for some isomers were too low, and (in these cases) a surrogate IS strategy with normalization to the more intense l-U-^13^C^15^N-Val signal was used. The calibration functions together with their linearity (in terms of *R*^2^), limit of detection (LOD), limit of quantification (LOQ), linear range, accuracy (as % recovery) and precision (% RSD) of a quality control sample (1 μM, *n* = 3), and the applied quantification method for d- and l-AQC-AA are summarized in Tables S23 and S24. The majority of d-AQC-AA meet the criteria for accuracy (85–115%) and precision (≤15%) as defined by the FDA.^56^ Only d-Ala (16.1%) and d-Thr (20.2%) do not meet the precision criteria, which might originate from insufficient correction by the surrogate IS (Table S23). Similarly, all l-amino acids meet both the accuracy and precision criteria except for l-His (accuracy of 83.2%), which might also originate from insufficient correction by the surrogate IS (Table S24). As previously stated, the resolution between d-Leu and d-Ile with this rapid method is not sufficient for accurate quantification, and only the sum of the corresponding constitutional Leu/Ile isomer pairs can be determined. For l-Leu and l-Ile their ratio can be approximated due to partial separation by enantioselective LC. The LOQ calculated according to ICH guideline Q2(R1)^57^ ranges from 0.16 μM for l-Phe up to 0.72 μM for d-Asp. Examples where the enantiomer concentrations were largely different are provided in Figures S26 and S27 and demonstrate the capability of the method to still provide baseline separation at such extreme enantiomer ratios. Overall, the performance appears to be adequate for application of relative quantification of the amino acid composition in synthetic and natural peptides.

## Conclusions

The in-line coupled tandem column consisting of two prototype core–shell chiral columns showed superior resolution compared to the single columns for a set of 24 AQC-derivatized amino acid enantiomer pairs and diastereomers and constitutional isomers of leucine and threonine. New insights into the capabilities of IM for resolving derivatized amino acid enantiomers could be derived from experimental results and additional computational assessments. Generally, IM resolution between key amino acid isomers was either equal to or reduced upon AQC derivatization. While the combination of DTIM separation and HRdm (or another type of IM analyzer with high native resolving power) can resolve almost all combinations of Thr, *a*Thr, and Hse isomers in a rapid analysis method, the mobility differences for AQC-derivatized Leu isomers are too small to be addressed by current IM technologies given the simultaneous requirements of comprehensive sampling of the fast LC separation (i.e., LC fwhm of <5 s) and high IM resolving powers of >200 for ions across a wide mass range. The new LC-IM-MS method offers capability to be used for routine, high-throughput quantitative analysis of amino acid enantiomers in pharmaceutical and nutritional products as well as food samples. The same strategy of enantioselective amino acid analysis by UHPLC with IM-MS detection could be applied to teicoplanin and TAG core–shell columns. However, both teicoplanin and TAG show the “wrong” enantiomer elution order (l before d; for free and AQC-derivatized amino acids), which makes it a bit inconvenient if the d-trace enantiomer is eluting on the tailing edge of the major l-enantiomer peak.

## Supporting Information Available

Additional information on the experimental section including materials used and sample preparation protocols, calculation of chromatographic performance, data analysis procedure for LC-IM-MS data, quantitative performance evaluation, DFT simulations, CCS values, EICs of enantiomer separation of single and tandem columns, standard and HRdm IM spectra, and retention time vs drift time plots of lipopeptide and octreotide hydrolysate samples (PDF)
