# Supplementary material for: Three-Minute Enantioselective Amino Acid Analysis by Ultra-High-Performance Liquid Chromatography Drift Tube Ion Mobility-Mass Spectrometry Using a Chiral Core–Shell Tandem Column Approach
Source: Anal Chem. 2024 Feb 1;96(6):2666–75. doi: 10.1021/acs.analchem.3c05426 (PMC10867800; doi:10.1021/acs.analchem.3c05426)
Supplement: Supplementary file 1 — ac3c05426_si_001.pdf [file ac3c05426_si_001.pdf]

**Supporting Information**

# Three-Minute Enantioselective Amino Acid Analysis by Ultra-High Performance Liquid Chromatography Drift Tube Ion Mobility-Mass Spectrometry Using a Chiral Core-Shell Tandem Column Approach

Simon Jonas Jaag[a], Younes Valadbeigi[b], Tim Causon[c,*], Harald Gross[d], Michael Lämmerhofer[a,*]

[a] Pharmaceutical (Bio-)Analysis, Institute of Pharmaceutical Sciences, University of Tuebingen, Auf der Morgenstelle 8, 72076 Tuebingen, Germany

[b] Department of Chemistry, Faculty of Science, Imam Khomeini International University, Nowrouzian, 3414896818, Qazvin, Iran

[c] University of Natural Resources and Life Sciences, Vienna Department of Chemistry, Institute of Analytical Chemistry, Muthgasse 18, 1190 Vienna, Austria

[d] Pharmaceutical Biology, Institute of Pharmaceutical Sciences, University of Tuebingen, Auf der Morgenstelle 8, 72076 Tuebingen, Germany

*Authors for correspondence:

Assoc. Prof. Tim Causon
University of Natural Resources and Life Sciences Vienna
Department of Chemistry
Institute of Analytical Chemistry
E-Mail: tim.causon@boku.ac.at

Prof. Dr. Michael Lämmerhofer
Pharmaceutical (Bio-)Analysis
Institute of Pharmaceutical Sciences
University of Tuebingen
Auf der Morgenstelle 8
72076 Tuebingen, Germany
T +49 7071 29 78793, F +49 7071 29 4565
E-Mail: michael.laemmerhofer@uni-tuebingen.de

**Table of Contents**

**Supplementary Note S1. Materials** ........ 7
**Supplementary Note S2. Manual sample preparation** ........ 8
**Supplementary Note S3. Automatized sample preparation using a pipetting robot** .....10
**Supplementary Note S4. Calculation of chromatographic parameters** ........16
**Supplementary Note S5. Data Analysis with LC-IM-MS data pre-processing** ........17
**Supplementary Note S6. Quantitative method performance** ........51

**Table S1.** Amino acid reference standards with corresponding 3-letter and 1-letter code (if available) for the proteinogenic (1-20) and isobaric amino acids (21-25) ........ 7
**Table S2.** Composition of the "cell-free" amino acid mix L-U-$^{13}C^{15}N$ based on the certificate of analysis from Cambridge Isotope Laboratories. ........ 8
**Table S3.** Concentration levels before and after AQC-derivatization. ........12
**Table S4.** Chromatographic performance parameters for the single QN-AX and ZWIX(+) column and the combined tandem column. Void times (tri-*tert*-butylbenzene): tandem: 0.44 min, QN-AX and ZWIX(+): 0.22 min. Same experimental conditions as in Figure S6. ........19
**Table S5.** Evaluation of the chromatographic performance of the leucine isomer separation in the 25 DL-AQC-AA mix sample. Tandem column: QN-AX + ZWIX(+) prototype core shell columns (3.0x50 mm, 2.7µm, respectively). Same LC conditions as in Figure S6. ........20
**Table S6**: Evaluation of the chromatographic performance of the separation of DL-Ile and DL-Leu containing sample without the other three Leu isomers. Tandem column: QN-AX + ZWIX(+) prototype core shell columns (3.0x50 mm, 2.7µm, respectively). Same LC conditions as in Figure S6. ........20
**Table S7.** Chromatographic performance evaluation of the threonine isomer mixture consisting of DL threonine (Thr), *allo*-threonine (*a*Thr) and homo-serine (Hse). Tandem column: QN-AX + ZWIX(+) prototype core shell columns (3.0x50 mm, 2.7µm, respectively). Same LC conditions as in Figure S6. ........20
**Table S8.** Chromatographic performance evaluation of the threonine isomer mixture consisting of DL-threonine (Thr) and DL-*allo*-threonine (*a*Thr). Tandem column: QN-AX + ZWIX(+) prototype core shell columns (3.0x50 mm, 2.7µm, respectively). Same LC conditions as in Figure S6. ........21
**Table S9**. The calculated $CCS_{N2}$ values and relative Gibbs free energies for the conformers of $[Trp+H]^+$. $^{DT}CCS_{N2}$ = 191.4 Å$^2$. ........24
**Table S10**. Calculation of the difference in the collisional cross section (CCS) of leucine and threonine isomers. ........24
**Table S11.** The calculated $CCS_{N2}$ values and relative Gibbs free energies for the conformers of $[Thr+H]^+$. $^{DT}CCS_{N2}$ = 174.1 Å$^2$. ........26
**Table S12.** The calculated $CCS_{N2}$ values and relative Gibbs free energies for the conformers of $[aThr+H]^+$. Experimental $^{DT}CCS_{N2}$ = 171.8 Å$^2$. ........29

**Table S13.** The calculated $CCS_{N2}$ values and relative Gibbs free energies for the conformers of $[HSe+H]^+$. Experimental $^{DT}CCS_{N2}$ = 172.8 Å². ....31
**Table S14.**The calculated $CCS_{N2}$ values and relative Gibbs free energies for the conformers of $[aIle+H]^+$. Experimental $^{DT}CCS_{N2}$ = 180.9 Å². ....33
**Table S15.**The calculated $CCS_{N2}$ values and relative Gibbs free energies for the conformers of $[Leu+H]^+$. Experimental $^{DT}CCS_{N2}$ = 180.9 Å². ....35
**Table S16.** The calculated $CCS_{N2}$ values and relative Gibbs free energies for the conformers of $[Ile+H]^+$. Experimental $^{DT}CCS_{N2}$ = 180.9 Å². ....37
**Table S17.** The calculated $CCS_{N2}$ values and relative Gibbs free energies for the conformers of *N*-methylphenylalanine $[NMePhe+H]^+$....39
**Table S18.** The calculated $CCS_{N2}$ values and relative Gibbs free energies for the conformers of *N*-methylvaline $[NMeVal+H]^+$. ....41
**Table S19.**The calculated $CCS_{N2}$ values and relative Gibbs free energies for the conformers of 3-hydroxy-*N*-methylvaline $[NMeValOH+H]^+$. ....43
**Table S20.** Comparison of the experimentally obtained and theoretically calculated CCS-values from the lipopeptide sample hydrolysate. The exact (calc.) *m/z* value of the protonated species is provided. ....44
**Table S21.** Comparison of experimentally obtained and theoretically calculated CCS-values from octreotide sample hydrolysate. The exact (calc.) *m/z* value of the protonated species is provided. Irregular amino acid: Threoninol (Thr-ol). ....44
**Table S22.** Comparison of the experimentally obtained and theoretically calculated CCS-values from the aureobasidin A sample hydrolysate. The exact (calc.) *m/z* value of the protonated species is provided. Irregular amino acids: 3-hydroxy-*N*-methylvaline (NMeValOH), 2-hydroxy-3-methylpentanoic acid (OIle), *N*-methylvaline (NMeVal), *N*-methylphenylalanine (NMePhe). ....45
**Table S23.** Calibration functions of the D-AQC-AAs including the linearity ($R^2$), the limit of detection (LOD), the limit of quantification (LOQ), the concentration range used for calibration, the accuracy and precision of a quality control sample and the applied quantification method. Either quantification based on light-to-heavy peak area ratios (L/H) or surrogate calibration with normalization of the peak areas by peak area of L-U-$^{13}C^{15}N$-Val (surrog.) was used depending on the signal intensity of the SIL-IS peak. ....52
**Table S24.** Calibration functions of the L-AQC-AAs including the linearity ($R^2$), the limit of detection (LOD), the limit of quantification (LOQ), the concentration range used for calibration, the accuracy and precision of a quality control sample and the applied quantification method. Either quantification based on light-to-heavy peak area ratios (L/H), surrogate calibration with normalization of the peak areas by peak area of L-U-$^{13}C^{15}N$-Val (surrog.) or calibration by normalization by total ion current (TIC) was used depending on most appropriate conditions. ....53

**Figure S1.** Starting deck configuration for the alkylation step of the amino acids. The reservoir (bottom left, slot 1) contains the 10 mL 0.4 M borate buffer pH 8.8 (well 1), 10 mL 10 mM IAA solution (well 2) and 10 mL 10 mM DTT solution (well 3). The sample plate (bottom centre, slot 1) contains the samples (calibrants, QCs, columns 1 to 4) and blanks (column 5)...............12
**Figure S2.** Final deck state after complete alkylation. .......................................................13
**Figure S3.** Start deck state of the AQC-derivatization step. The reservoir (bottom left, slot 1) contains the 10 mL 0.4 M borate buffer pH 8.8 (well 1), 10 mL 10 mM IAA solution (well 2) and 10 mL 10 mM DTT solution (well 3). The derivatization chamber (bottom centre, slot 2) contains 120 µL 14 mM AQC reagent (dissolved in ACN) in each well of column 12. The alkylated sample plate originates from the alkylation step 120 µL of the AQC-derivatized and racemised SIL-IS mix were added in column 12 prior running the protocol. ..........................................14
**Figure S4.** Final deck state after AQC-derivatization and IS spiking. ..................................15
**Figure S5**. Structures of the stationary phase of the (a) QN-AX column and of the (b) ZWIX(+) column. ........................................................................................................................15
**Figure S6.** Extracted ion chromatograms (EIC) of the AQC-derivatized (a) arginine, (b) aspartic acid, (c) asparagine and (d) proline LC enantiomer separation using the single QN-AX, ZWIX(+) or the combined tandem column. Mobile phase A: ACN/MeOH/$H_2O$ 49:49:2 (v/v/v) 10 mM $NH_4FA$ + 10 mM FA, mobile phase B: ACN/MeOH/$H_2O$ 49:49:2 (v/v/v) 50 mM $NH_4FA$ + 50 mM FA; flow rate: 1.25 mL/min, gradient: 0-0.4 min 0 %B, 0.4-1.0 min 0-100 %B, 1.0-3.0 min 100 %B, 3.0-3.2 min 100-0 %B, 3.2-4.0 min 0 %B, column temperature: 50°C..18
**Figure S7.** Standard arrival time spectra (a) and high-resolution demultiplexed arrival time spectra (b). The peak labels are the corresponding $^{DT}CCS_{N2}$ values [Å²]. The broader IM peak observed for D-Thr following HRdm is due to the low signal intensity of this compound in the examples shown. Assessment of higher D-Thr concentrations or additional fine-tuning of HRdm settings provide the same peak width and arrival time as L-Thr. ..........................................22
**Figure S8.** Impact of AQC derivatization on $^{DT}CCS_{N2}$ in comparison to corresponding protonated amino acids. Data for underivatized amino acids retrieved from the CCS Compendium (https://mcleanresearchgroup.shinyapps.io/CCS-Compendium)....................23
**Figure S9**. Optimized structures of different conformers of $[Trp+H]^+$ in the gas phase and their calculated $CCS_{N2}$ values. The numbers in parenthesis are the relative Gibbs free energies in kJ $mol^{-1}$. The calculated $CCS_{N2}$ values of the most stable isomers **b** and **e** (190.7 and 190.5 Å$^2$) are in agreement with the experimental $^{DT}CCS_{N2}$ (191.4 Å$^2$) indicating the twisted structure of $[Trp+H]^+$ in the gas phase because of a π-π interaction. ................................................23
**Figure S10.** Optimized structures of different conformers of $[Thr+H]^+$ in the gas phase and their calculated $CCS_{N2}$ values. The numbers in parenthesis are the relative Gibbs free energies in kJ $mol^{-1}$. .....................................................................................................25
**Figure S11.** Arrival time spectra of single D-leucine isomers standards after application of high-resolution de-multiplexing. Expected Leu isomers in real samples cannot be resolved in complex mixtures using IM with HRdm. ....................................................................27

**Figure S12.** Optimized structures of different conformers of $[aThr+H]^+$ in the gas phase and their calculated $CCS_{N2}$ values. The numbers in parenthesis are the relative Gibbs free energies in kJ $mol^{-1}$. ........ 28
**Figure S13.** Optimized structures of different conformers of $[HSe+H]^+$ in the gas phase and their calculated $CCS_{N2}$ values. The numbers in parenthesis are the relative Gibbs free energies in kJ $mol^{-1}$. ........ 30
**Figure S14.** Optimized structures of different conformers of $[aIle+H]^+$ in the gas phase and their calculated $CCS_{N2}$ values. The numbers in parenthesis are the relative Gibbs free energies in kJ $mol^{-1}$. ........ 32
**Figure S15.** Optimized structures of different conformers of $[Leu+H]^+$ in the gas phase and their calculated $CCS_{N2}$ values. The numbers in parenthesis are the relative Gibbs free energies in kJ $mol^{-1}$. ........ 34
**Figure S16.** Optimized structures of different conformers of $[Ile+H]^+$ in the gas phase and their calculated $CCS_{N2}$ values. The numbers in parenthesis are the relative Gibbs free energies in kJ $mol^{-1}$. ........ 36
**Figure S17.** Optimized structures of different conformers of *N*-methylphenylalanine $[NMePhe+H]^+$ in the gas phase and their calculated $CCS_{N2}$ values. The numbers in parenthesis are the relative Gibbs free energies in kJ $mol^{-1}$. ........ 38
**Figure S18.** Optimized structures of different conformers of *N*-methylvaline $[NMeVal+H]^+$ in the gas phase and their calculated $CCS_{N2}$ values. The numbers in parenthesis are the relative Gibbs free energies in kJ $mol^{-1}$. ........ 40
**Figure S19.** Optimized structures of different conformers of 3-hydroxy-*N*-methylvaline $[NMeValOH+H]^+$ in the gas phase and their calculated $CCS_{N2}$ values. The numbers in parenthesis are the relative Gibbs free energies in kJ $mol^{-1}$. ........ 42
**Figure S20**. NRPS gene cluster of the investigated lipopeptide. Each module consists of a condensation (C), an adenylation (A) and a carrier protein (CP) domain. The adenylation domain recognizes and activates a specific amino acid and hands it over to the CP domain. C domains fuse two amino acids that are presented by CP domains. Finally, the formed peptide string is cleaved off the mega-enzyme with the help of a thioesterase domain (TE). The red boxes indicate modules 8 and 11, responsible for the recognition and activation of Gln and Glu, respectively. Dual epimerization/condensation domains are capable to convert the absolute configuration of the alpha-proton of the amino acid of the preceding module. ........ 46
**Figure S21**. HRdm arrival time spectra of the lipopeptide hydrolysate sample and single D-Thr, D-Hse and D-*a*Thr standards. Same conditions as in Figure 5. ........ 46
**Figure S22.** Retention time vs. arrival time plot for the (a) lipopeptide hydrolysate, (b) D-Thr, (c) D-*a*Thr and (d) D-Hse standards. Same conditions as in Figure 5. ........ 47
**Figure S23.** EICs of leucine isomers for the lipopeptide hydrolysate sample, 25 DL AQC-AA mix and single (a) D-AA and single (b) L-AA reference standard injections. Same experimental conditions as in Figure 5. ........ 48

**Figure S24.** Retention time vs. drift time plot for the (a) lipopeptide hydrolysate, (b) D-*t*Leu, (c) D-*n*Leu, (d) D-*a*Ile, (e) D-Ile, (f) D-Leu, (g) L-*t*Leu, (h) L-*n*Leu, (i) L-*a*Ile, (j) L-Ile and (k) L-Leu. Same conditions as in Figure S22. ....................................................................................49

**Figure S25.** Enantioselective analysis of the peptide octreotide after its full hydrolysis**.** (a) Structure of octreotide. (b-g), EICs of the respective AQC-derivatized amino acids and drift time vs. *m/z* contour plots from threonine isomers of (h) octreotide hydrolysate, (i) L-*a*Thr and L-Thr single standards. Same experimental conditions as in Figure 5..................................50

**Figure S26.** Extracted ion chromatograms (EICs) of AQC-derivatized L-Hse and D-Hse standards. The D-Hse standard has an L-Hse impurity peak with 4.8 % of the total peak area, documenting the applicability of the method for amino acids with extreme enantiomer ratio. 54

**Figure S27.** Extracted ion chromatograms (EICs) of single AQC-derivatized L-Phe and D-Phe standards and an additional lipopeptide sample b (after hydrolysis and AQC-derivatization) which has a small D-Phe peak (0.5 % peak area) while the majority is L-Phe (99.5 %), documenting the applicability of the method for amino acids with extreme enantiomer ratio. 54

## Supplementary Note S1. Materials

Octreotide and aureobasidin A peptide samples were obtained from Avachem Scientific (San Antonio, TX, USA) and BOC Sciences (Shirley, NY, USA), respectively. 6-Aminoquinolyl-*N*-hydroxysuccinimidyl carbamate (AQC) was purchased from BLDpharm (Kaiserslautern, Germany). D- and L-amino acids (see Supporting Information Table S1) were from Sigma Aldrich (Schnelldorf, Germany). A uniformly L-$^{13}C^{15}N$ (U-$^{13}C^{15}N$)-labelled cell free amino acid mixture as internal standard (IS, for composition see Supporting Information Table S2) was purchased from Cambridge Isotope Laboratories (Andover, MA, USA). Acetonitrile (ACN), formic acid (FA) and methanol (MeOH) were from Carl Roth (Karlsruhe, Germany) or Sigma Aldrich (Vienna, Austria).

Prototype core-shell ZWIX(+) and QN-AX columns (both 3.0x50mm, 2.7 µm, 160 Å) were from former studies (Schmitt et al.[1]; Geibel et al.[2]). Ammonium formate ($NH_4FA$), boric acid, deuterium chloride (DCl), deuterium oxide ($D_2O$), dithiothreitol (DTT), hydrochloric acid (HCl), iodoacetamide (IAA) and sodium hydroxide (NaOH) were purchased from Sigma Aldrich. Ultra-pure water was provided from a Water Purelab Analytics Purification system from Elga (Celle, Germany) or a Milli-Q IQ 7000 purification system equipped with an LC-Pak polisher cartridge (Merck Chemicals and Life Science GmbH, Vienna).

**Table S1.** Amino acid reference standards with corresponding 3-letter and 1-letter code (if available) for the proteinogenic (1-20) and isobaric amino acids (21-25)

| | | |
|---|---|---|
| 1. Aspartic acid (D, Asp) | 10. Arginine (R, Arg) | 19. Alanine (A, Ala) |
| 2. Methionine (M, Met) | 11. Histidine (H, His) | 20. Threonine (T, Thr) |
| 3. Asparagine (N, Asn) | 12. Isoleucine (I, Ile) | 21. *allo*-Threonine (*a*Thr) |
| 4. Glutamine (Q, Gln) | 13. Leucine (L, Leu) | 22. Homoserine (Hse) |
| 5. Tryptophan (W, Trp) | 14. Cysteine (C, Cys) | 23. *allo*-Isoleucine (*a*Ile) |
| 6. Tyrosine (Y, Tyr) | 15. Glycine (G, Gly) | 24. *tert*-Leucine (*t*Leu) |
| 7. Glutamic acid (E, Glu) | 16. Valine (V, Val) | 25. *nor*-Leucine (*n*Leu) |
| 8. Lysine (K, Lys) | 17. Serine (S, Ser) | |
| 9. Proline (P, Pro) | 18. Phenylalanine (F, Phe) | |

**Table S2.** Composition of the "cell-free" amino acid mix L-U-$^{13}C^{15}N$ based on the certificate of analysis from Cambridge Isotope Laboratories.

| amino acid | molar [%] | weight [%] |
|---:|---:|---:|
| Ala | 12.1 | 8.2 |
| Arg | 2.8 | 3.7 |
| Asn | 4.0 | 4.0 |
| Asp | 4.6 | 4.6 |
| Cys | 0.6 | 1.1 |
| Gln | 3.7 | 4.1 |
| Glu | 3.4 | 3.7 |
| Gly | 7.0 | 3.9 |
| His | 0.4 | 0.4 |
| Ile | 7.0 | 7.0 |
| Leu | 11.5 | 11.5 |
| Lys | 9.6 | 10.7 |
| Met | 1.1 | 1.3 |
| Phe | 5.9 | 7.4 |
| Pro | 2.7 | 2.4 |
| Ser | 3.2 | 2.5 |
| Thr | 2.8 | 2.5 |
| Trp | 5.5 | 8.6 |
| Tyr | 3.1 | 4.3 |
| Val | 9.0 | 8.0 |

## Supplementary Note S2. Manual sample preparation

### Peptide hydrolysis

1 mg of lipopeptide sample was dissolved in 6 M DCl in $D_2O$ and sealed in a 1.5 mL glass hydrolysis vial under nitrogen protection. Full hydrolysis was carried out at 110°C for 16 h. The sample was evaporated to dryness and re-dissolved in 1 mL 0.4 M sodium borate buffer pH 8.8, vortexed and centrifuged at 16,000 g for 60 s.

### Stock solutions

Amino acid stock solutions were prepared at a concentration of 62.5 mM in 0.1 M HCl except for tyrosine which was dissolved in 0.5 M HCl. The following mixtures of amino acids were prepared by mixing the amino acid stock solutions and dilution with 0.1 M HCl where required: Single amino acid reference standards for both D- and L-enantiomers separately at 2.5 mM. A Mix of 25 proteinogenic amino acids (see Table S1) with c = 2.5 mM for the D- and L-enantiomers, respectively. A mix of D-Leu:L-Leu:D-Ile:L-Ile and D-Thr:L-Thr:D-*a*Thr:L-*a*Thr with 1:2:3:4 mM, respectively.

**AQC-derivatization**

For *S*-alkylation, 50 µL of sample were mixed with 250 µL of 0.4 M sodium borate buffer pH 8.8 and 25 µL of 10 mM DTT. After each mixing step the sample was vortexed 60 s and centrifuged at 3,000g for 60 s prior incubation. Reduction of disulfides by DTT was allowed to proceed for 10 min at 55°C. Subsequently, samples were alkylated by addition of 50 µL of 10 mM IAA and incubated for 10 min at 55°C. Unreacted IAA was quenched by addition of 25 µL DTT and incubation for 10 min at 55°C. 200 µL of this solution was transferred to a new vial and diluted with 50 µL 0.4 M sodium borate buffer. 55.5 µL of this solution was mixed with 394.5 µL of 0.4 M sodium borate buffer and 50 µL of 4 mg/mL AQC solution (in ACN) and incubated for 10 min at 55°C.

**Preparation of U-$^{13}C^{15}N$-labelled IS and spiking**

A U-$^{13}C^{15}N$-amino acid mix consisting of the 20 proteinogenic L-amino acids (c = 5 mg/mL in 0.1 M HCl) was alkylated and AQC-derivatized in the same way as described above and then racemised by incubation at 95°C for 6 h. 450 µL of sample and 50 µL of internal standard were mixed together.

For long-term storage and transportation, the samples were aliquoted to 100 µL, lyophilised at 0.050 mbar overnight using a FreeZone Benchtop Freeze Dryer 4.5 L, -105°C from Labconco (Kansas City, MO, USA). Prior to usage, the samples were reconstituted with 100 µL $H_2O$/MeOH 1:1 (v/v), vortexed 60 s, centrifuged at 3,000 g for 60 s and finally transferred to an HPLC vial with micro insert.

A second sample set with a calibrant series was prepared using a pipetting robot and further details can be found in the Supplementary Note S3 (Table S3 and Figures S1-4).

## Supplementary Note S3. Automatized sample preparation using a pipetting robot

### Automatized sample preparation using the OT-2 pipette robot

An OT-2 pipette robot from Opentrons (New York City, NY, USA) was used and equipped with a P20 8-channel Gen 2 (20 µL), a P300 8-channel Gen 2 (300 µL) pipette and a temperature module Gen 2. The original Opentrons pipette tips for the 20 µL and 300 µL pipettes were used. A 12-well reservoir with 22 mL wells (Starlab, Hamburg, Germany), 96-well PCR plate with 200 µL wells (HSP9601) from Bio-Rad (Feldkirchen, Germany) and a PierceASeal Foil heat sealable seal (Sigma-Aldrich) were used.

Due to high consumption of pipette tips the protocol was divided into two steps: First the alkylation was performed and second the AQC-derivatization and SIL-IS spiking was performed. All mentioned transfer volumes are per well. Tips were changed before each aspiration step, tip position for aspiration was 1 mm above the bottom and for dispensing 0.5 mm above the bottom. The “blowout” function was used at the dispensing step at the destination well. For mixing of the solutions the mixing function was used which comprises of a specified number of aspiration and dispensing steps with a pre-defined mixing volume. After each heating step the well plate was centrifuged (Eppendorf Centrifuge 5415 R) at 3,000 rpm for 3 min to recover condensate from the sealing foil. A calibration series was prepared manually with the concentrations levels as described in Table S3.

### Alkylation

The starting deck configuration is shown in Figure S1. Samples and blanks (10 µL) were transferred from the sample plate to the reaction chamber plate, then 5 µL of the DTT solution and 70 µL borate buffer were added and a mixing step with 40 µL mix volume was done three times. The protocol was paused and the reaction chamber was sealed with an aluminium foil to prevent evaporation and the plate was put onto the heater block module (slot 10) and reduction was done for 10 min at 55°C. After alkylation the plate was cooled down, the foil was removed and the plate put back to its original slot 3. The protocol was continued with the transfer of 10 µL of IAA solution, then a mixing with 20 µL mixing volume and three replications was performed. The plate was sealed and transferred to the heater module and alkylation was done for 10 min at 55°C. After alkylation the plate was moved back to slot 3 and cooled down to room temperature. The foil was removed and 5 µL DTT was added and mixing with a 20 µL volume was performed in triplicate. The plate was sealed again and the alkylation quenched for 10 min at 55°C on the heater module. After quenching the plate was moved back, cooled down and the foil was removed. The final deck state configuration is shown in Figure S2.

**AQC derivatization and SIL-IS spiking**

The starting deck for AQC derivatization and SIL-IS spiking is shown in Figure S3. The alkylated sample plate from the previous protocol step was used and AQC-derivatized and racemised SIL-IS was added to column 12. In the first step the samples and blanks (11 µL) from the alkylated sample plate (slot 3) were transferred to the derivatization chamber plate (slot 2). Then 79 µL borate buffer and 10 µL AQC solution were transferred and mixed with 20 µL mixing volume three times. The plate was sealed and transferred to the temperature module and derivatization was done for 10 min at 55°C. After derivatization the plate was moved back to slot 3, cooled down and the foil was removed. The SIL-IS (8 µL) was transferred to two new well plates (“IS spiked sample” and “IS spiked sample 2”) to obtain duplicate well plates (one for initial analysis, the second for storage). The AQC-derivatized samples and blanks (72 µL) were transferred to the SIL-IS containing plates and mixing performed three times with 65 µL mixing volume. The final deck state configuration is shown in Figure S4. The sample plates can be directly analysed using the 96-well plates seal by a foil or submitted for lyophilisation for long-term storage.

**Long term storage and reconstitution prior analysis**

For long-term storage the samples can be transferred to 1.5 mL eppi tubes and lyophilised at 0.050 mbar overnight using a FreeZone Benchtop Freeze Dryer 4.5 L, -105°C from Labconco (Kansas City, MO, USA). Prior usage, the samples were reconstituted with 100 µL $H_2O$/MeOH 1:1 (v/v), vortexed 60 s, centrifuged at 3,000 g for 60 s and finally transferred to an HPLC vial with micro insert. Lyophilised samples can be stored a -20°C.

**Table S3.** Concentration levels before and after AQC-derivatization.

| concentration level | c(prior derivat-ization) [µM] | c(final) [µM] |
|---|---|---|
| C8 | 1,000 | 10 |
| C7 | 200 | 2 |
| C6 | 150 | 1.5 |
| C5 | 80 | 0.8 |
| C4 | 60 | 0.6 |
| C3 | 40 | 0.4 |
| C2 | 20 | 0.2 |
| C1 | 10 | 0.1 |

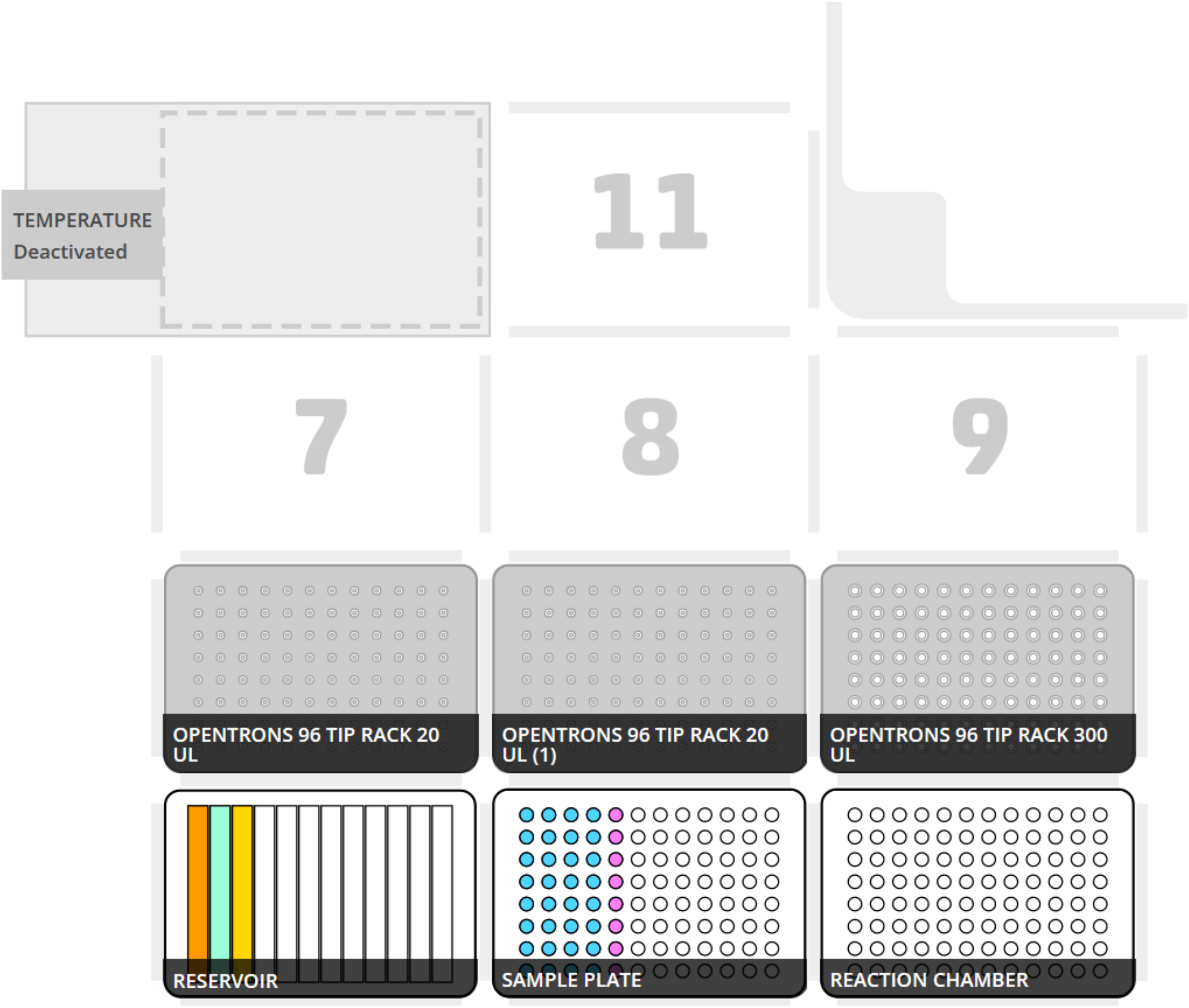


**Figure S1.** Starting deck configuration for the alkylation step of the amino acids. The reservoir (bottom left, slot 1) contains the 10 mL 0.4 M borate buffer pH 8.8 (well 1), 10 mL 10 mM IAA solution (well 2) and 10 mL 10 mM DTT solution (well 3). The sample plate (bottom centre, slot 1) contains the samples (calibrants, QCs, columns 1 to 4) and blanks (column 5).

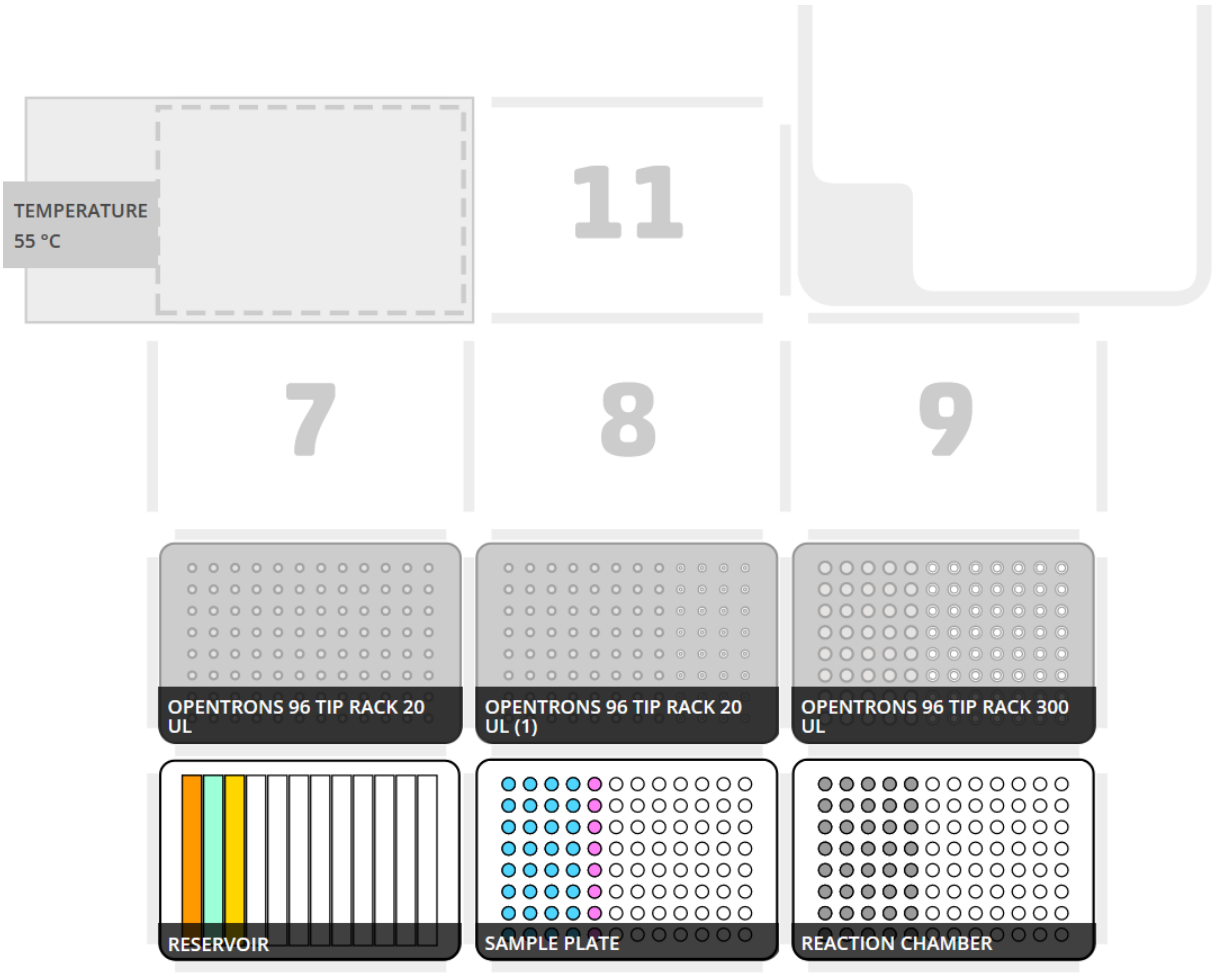


**Figure S2.** Final deck state after complete alkylation.

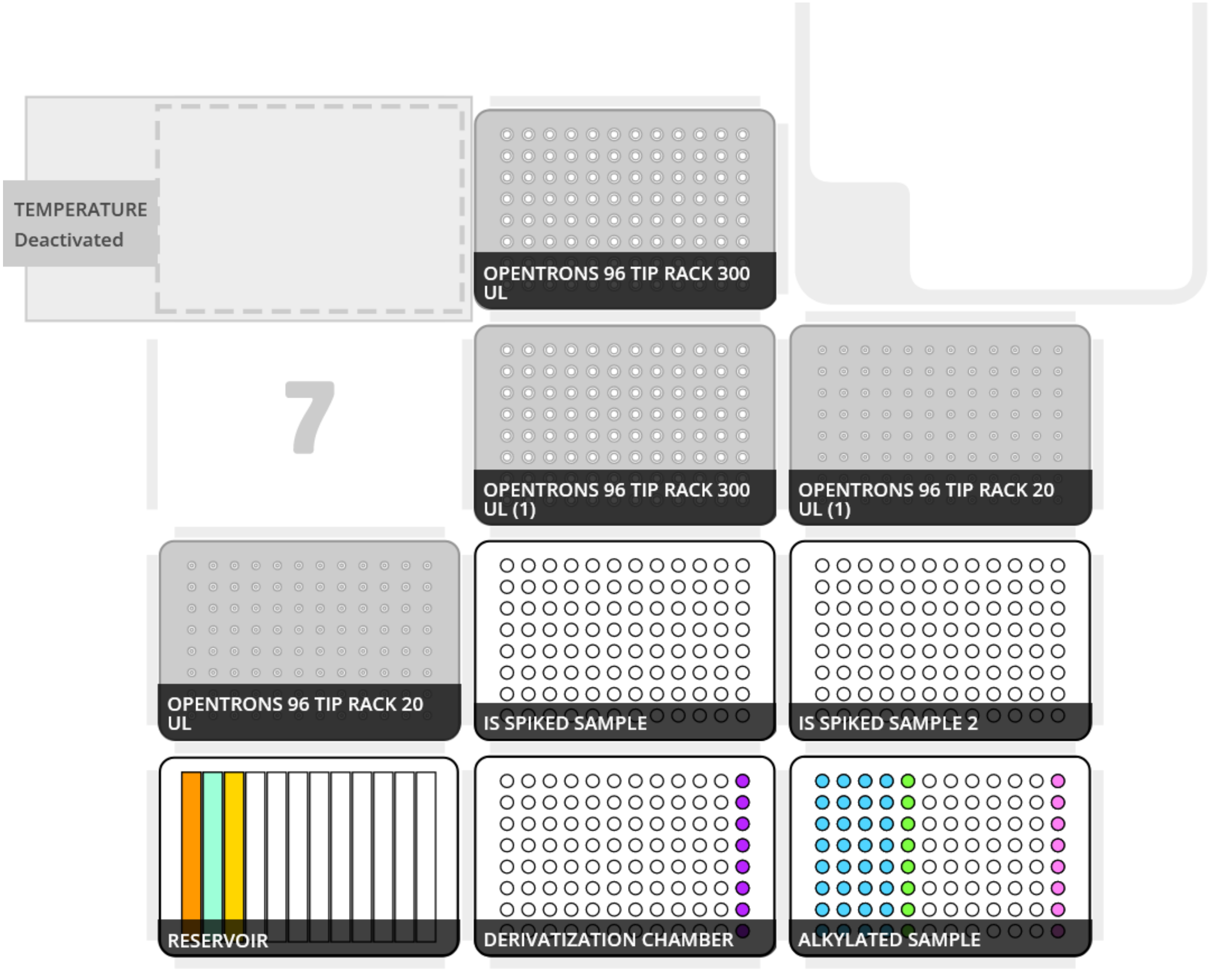


**Figure S3.** Start deck state of the AQC-derivatization step. The reservoir (bottom left, slot 1) contains the 10 mL 0.4 M borate buffer pH 8.8 (well 1), 10 mL 10 mM IAA solution (well 2) and 10 mL 10 mM DTT solution (well 3). The derivatization chamber (bottom centre, slot 2) contains 120 µL 14 mM AQC reagent (dissolved in ACN) in each well of column 12. The alkylated sample plate originates from the alkylation step 120 µL of the AQC-derivatized and racemised SIL-IS mix were added in column 12 prior running the protocol.

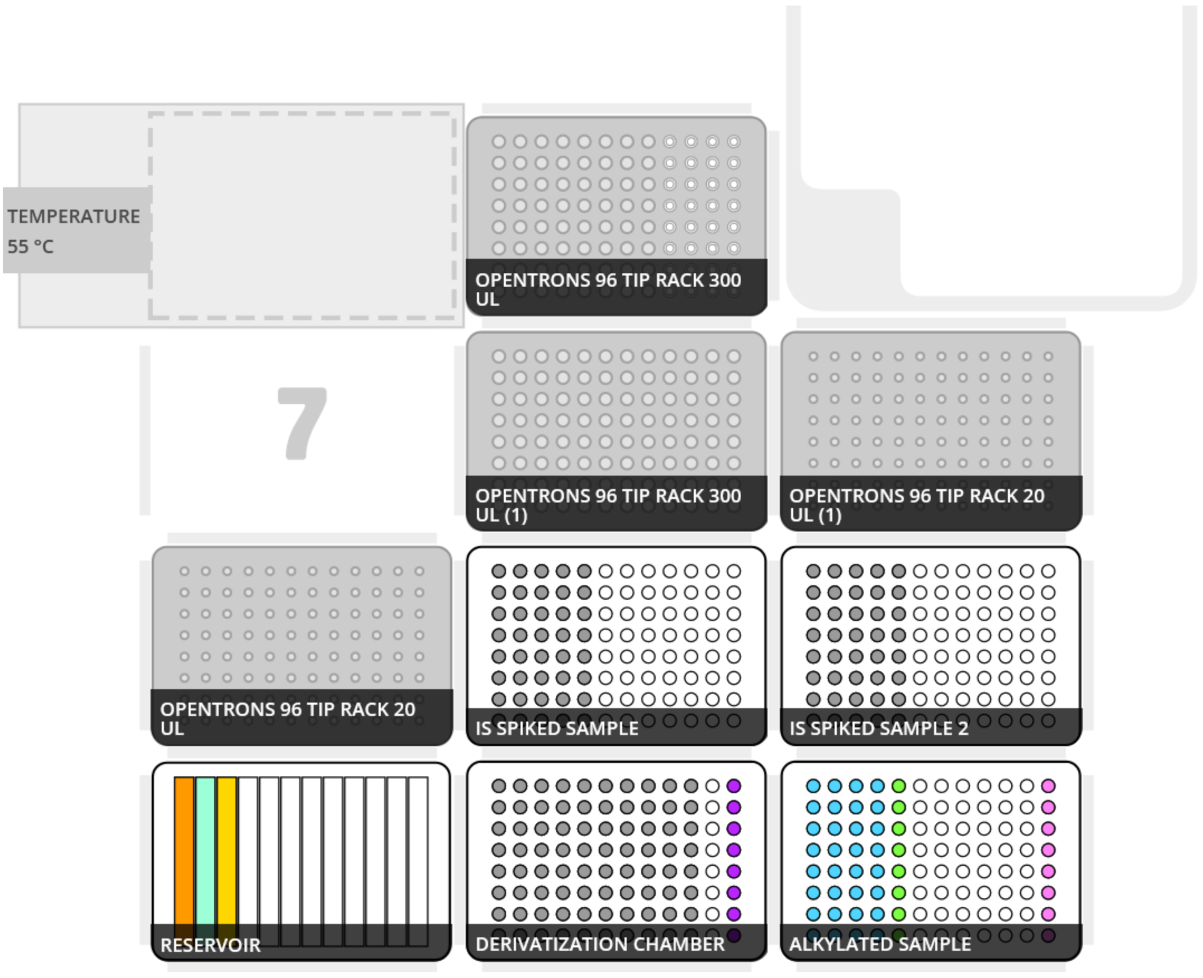


**Figure S4.** Final deck state after AQC-derivatization and IS spiking.

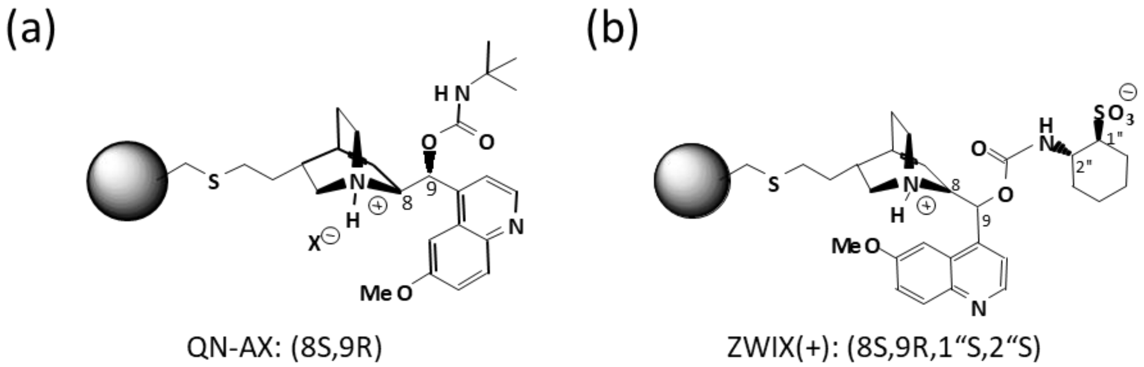


**Figure S5**. Structures of the employed chiral stationary phases. (a) QN-AX column and (b) ZWIX(+) column.

**Supplementary Note S4. Calculation of chromatographic parameters**

The resolution $R_s$ was calculated by:

$$R_s = 1.18 \cdot \frac{t_{R_2} - t_{R_1}}{w_{1/2_1} + w_{1/2_2}}$$

(eq. 1)

Wherein $w_{1/2}$ is the peak width at half height for the respective peaks and $t_{R2}$ and $t_{R1}$ as the retention times of the second and first eluting peaks, respectively.

**Supplementary Note S5. Data Analysis with LC-IM-MS data pre-processing**

**Standard workflow**

1. Using the PNNL Pre-processor, datafiles were subject to summing of adjacent frames and minimum pulse coverage set to 50% for de-multiplexing.
2. Datafiles were mass re-calibrated with purine and HP-921 used as reference masses.
3. Datafiles were *CCS* calibrated using vendor software (IM-MS Browser).
4. Peak-picking was performed using vendor software (IM-MS Browser).

**High-resolution demultiplexing (HRdm) workflow**

The application of high-resolution demultiplexing was already described by Butler et al.[3] and May et al.[4]

1. Using the PNNL Pre-processor, 3-point interpolation of arrival time spectra was applied in addition to the standard demultiplexing workflow settings. No summing of adjacent frames was used.
5. Peak-picking was performed using vendor software (IM-MS Browser).
2. Each feature list (per data file) was exported into the vendor *.CEF format and HRdm applied using the following settings: HR processing level – medium; m/z width multiplier – 6; IF multiplier – 0.95.
3. Datafiles were mass re-calibrated with purine and HP-921 used as reference masses.
4. HRdm datafiles were *CCS* calibrated using vendor software (IM-MS Browser).
5. Peak-picking for HRdm datafiles was performed using vendor software (IM-MS Browser).

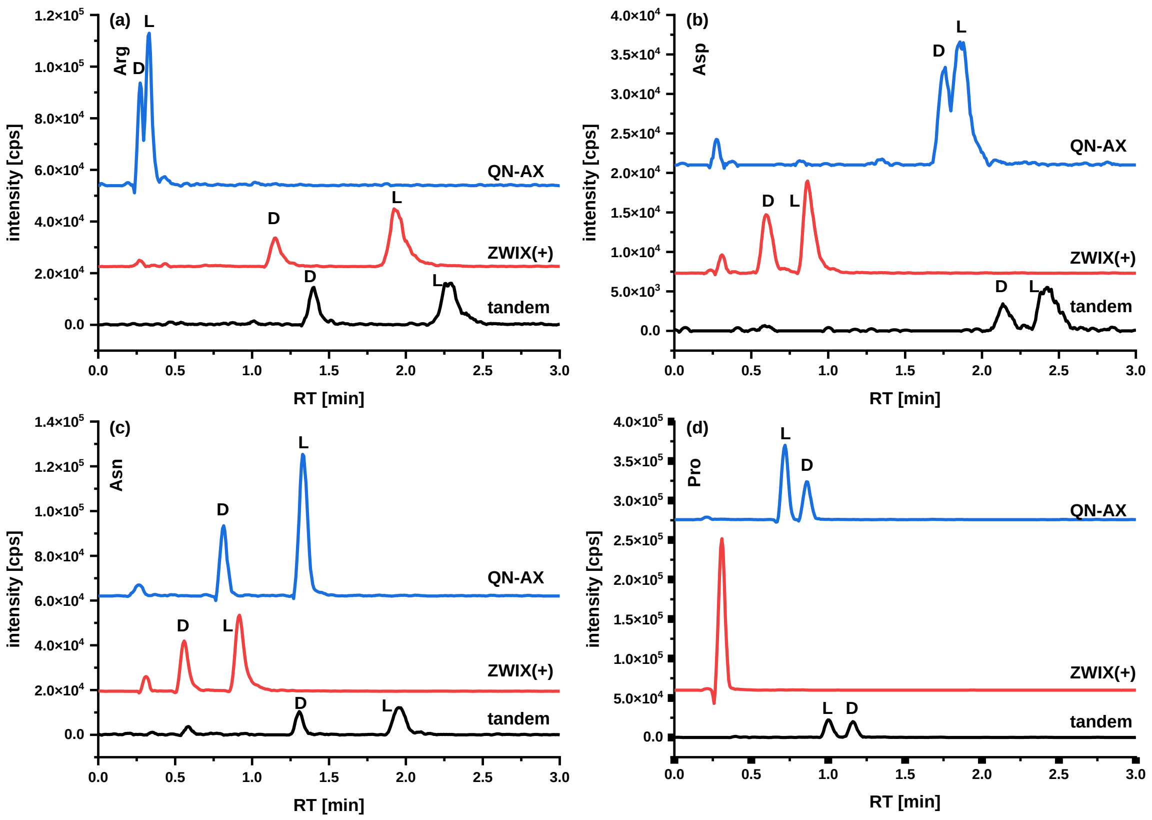


**Figure S6.** Extracted ion chromatograms (EIC) of the AQC-derivatized (a) arginine, (b) aspartic acid, (c) asparagine and (d) proline LC enantiomer separation using the single QN-AX, ZWIX(+) or the combined tandem column. Mobile phase A: ACN/MeOH/$H_2O$ 49:49:2 (v/v/v) 10 mM $NH_4FA$ + 10 mM FA, mobile phase B: ACN/MeOH/$H_2O$ 49:49:2 (v/v/v) 50 mM $NH_4FA$ + 50 mM FA; flow rate: 1.25 mL/min, gradient: 0-0.4 min 0 %B, 0.4-1.0 min 0-100 %B, 1.0-3.0 min 100 %B, 3.0-3.2 min 100-0 %B, 3.2-4.0 min 0 %B, column temperature: 50°C. Enantiomer ratio, D:L=1:2.

**Table S4.** Chromatographic performance parameters for the single QN-AX and ZWIX(+) column and the combined tandem column. Void times (tri-*tert*-butylbenzene): tandem: 0.44 min, QN-AX and ZWIX(+): 0.22 min. Same experimental conditions as in Figure S6.

| | QN-AX | | | ZWIX(+) | | | tandem | | |
|---|---|---|---|---|---|---|---|---|---|
| **AQC-AA** | **RT(D) [min]** | **RT(L) [min]** | **$R_s$** | **RT(D) [min]** | **RT(L) [min]** | **$R_s$** | **RT(D) [min]** | **RT(L) [min]** | **$R_s$** |
| *a*Ile | 0.27 | 0.32 | 3.30 | 1.16 | 1.93 | 0.00 | 1.39 | 2.30 | 4.27 |
| Ala | 0.59 | 0.79 | 1.97 | 0.35 | 0.38 | 0.44 | 0.94 | 1.15 | 2.75 |
| Arg | 0.82 | 1.36 | 0.98 | 0.57 | 0.93 | 6.49 | 1.31 | 1.97 | 7.67 |
| Asn | 1.75 | 1.85 | 7.08 | 0.61 | 0.87 | 3.86 | 2.19 | 2.44 | 7.08 |
| Asp | 0.85 | 1.15 | 0.79 | 0.54 | 0.76 | 2.36 | 1.28 | 1.67 | 2.11 |
| *aT*hr | 0.73 | 0.95 | 4.06 | 0.46 | 0.60 | 2.79 | 1.17 | 1.41 | 3.70 |
| bis-AQC-Lys | 0.77 | 1.05 | 4.13 | 0.73 | 0.91 | 1.93 | 1.38 | 1.70 | 3.78 |
| Cys-IAA | 1.34 | 1.47 | 3.93 | 0.48 | 0.58 | 2.88 | 1.70 | 1.84 | 4.60 |
| Gln | 0.86 | 0.86 | 2.60 | 0.53 | 0.53 | 1.84 | 1.32 | 1.32 | 2.83 |
| Glu | 0.43 | 0.65 | 1.92 | 0.90 | 1.43 | 1.31 | 1.28 | 1.91 | 1.27 |
| Gly | 0.45 | 0.87 | n/a | 0.31 | 0.31 | n/a | 0.68 | 1.06 | n/a |
| His | 0.45 | 0.71 | 3.25 | 0.31 | 0.31 | 4.17 | 0.75 | 1.06 | 5.72 |
| Hse | 0.38 | 0.71 | 3.30 | 0.31 | 0.31 | 2.36 | 0.60 | 1.01 | 2.83 |
| Ile | 0.45 | 0.71 | 3.30 | 0.31 | 0.31 | 0.00 | 0.75 | 1.06 | 4.08 |
| Leu | 0.45 | 0.87 | 2.56 | 0.31 | 0.31 | 0.00 | 0.68 | 1.15 | 2.29 |
| Met | 0.71 | 0.99 | 3.67 | 0.39 | 0.44 | 0.98 | 1.07 | 1.34 | 3.98 |
| *n*Leu | 0.69 | 1.03 | 3.41 | 0.35 | 0.43 | 0.00 | 1.04 | 1.36 | 4.06 |
| Phe | 0.86 | 0.73 | 5.02 | 0.31 | 0.31 | 1.57 | 1.18 | 1.02 | 3.78 |
| Pro | 0.72 | 1.05 | 1.70 | 0.44 | 0.83 | 0.00 | 1.13 | 1.62 | 1.89 |
| Ser | 0.61 | 1.05 | 4.33 | 0.35 | 0.61 | 4.60 | 0.98 | 1.52 | 5.78 |
| Thr | 0.70 | 1.01 | 5.19 | 0.35 | 0.61 | 2.79 | 0.98 | 1.45 | 3.75 |
| *t*Leu | 0.61 | 0.89 | 4.87 | 0.35 | 0.51 | 0.00 | 0.98 | 1.34 | 4.03 |
| Trp | 0.79 | 1.19 | 4.72 | 0.51 | 1.31 | 8.58 | 1.27 | 2.19 | 9.87 |
| Tyr | 0.71 | 1.09 | 4.98 | 0.41 | 0.57 | 2.70 | 1.09 | 1.50 | 5.38 |
| Val | 0.46 | 0.86 | 6.74 | 0.27 | 0.31 | 1.18 | 0.71 | 1.15 | 5.77 |

**Table S5.** Evaluation of the chromatographic performance of the leucine isomer separation in the 25 DL-AQC-AA mix sample. Tandem column: QN-AX + ZWIX(+) prototype core shell columns (3.0x50 mm, 2.7µm, respectively). Same LC conditions as in Figure S6.

| AQC-AA | RT [min] | $w_{1/2}$ [min] | $t'_R$ [min] | $R_s$ | $^{DT}CCS_{N2}$ [Å²] |
|---|---|---|---|---|---|
| D-*t*Leu | 0.60 | 0.04 | 0.16 | n/a | 181.6 |
| D-*a*Ile | 0.68 | 0.04 | 0.24 | 1.18 | 180.9 |
| D-Ile | 0.68 | 0.05 | 0.24 | 0.00 | 180.9 |
| D-Leu | 0.75 | 0.06 | 0.31 | 0.75 | 180.9 |
| D-*n*Leu | 0.75 | 0.06 | 0.31 | 0.00 | 182.1 |
| L-*t*Leu | 1.01 | 0.08 | 0.57 | 2.19 | 181.6 |
| L-Ile | 1.06 | 0.06 | 0.62 | 0.42 | 180.9 |
| L-Leu | 1.06 | 0.10 | 0.62 | 0.00 | 180.9 |
| L-*n*Leu | 1.06 | 0.03 | 0.62 | 0.00 | 182.1 |
| L-*a*Ile | 1.15 | 0.09 | 0.71 | 0.88 | 180.9 |

**Table S6**: Evaluation of the chromatographic performance of the separation of DL-Ile and DL-Leu containing sample without the other three Leu isomers. Tandem column: QN-AX + ZWIX(+) prototype core shell columns (3.0x50 mm, 2.7µm, respectively). Same LC conditions as in Figure S6.

| AQC-AA | RT [min] | $w_{1/2}$ [min] | $t'_R$ [min] | $R_s$ | $^{DT}CCS_{N2}$ [Å²] |
|---|---|---|---|---|---|
| D-Ile | 0.69 | 0.04 | 0.25 | n/a | 180.9 |
| D-Leu | 0.73 | 0.03 | 0.29 | 0.67 | 180.9 |
| L-Leu | 1.02 | 0.06 | 0.58 | 3.80 | 180.9 |
| L-Ile | 1.1 | 0.06 | 0.66 | 0.79 | 180.9 |

**Table S7.** Chromatographic performance evaluation of the threonine isomer mixture consisting of DL threonine (Thr), *allo*-threonine (*a*Thr) and homo-serine (Hse). Tandem column: QN-AX + ZWIX(+) prototype core shell columns (3.0x50 mm, 2.7µm, respectively). Same LC conditions as in Figure S6.

| AQC-AA | RT [min] | $w_{1/2}$ [min] | $t'_R$ [min] | $R_s$ | $^{DT}CCS_{N2}$ [Å²] |
|---|---|---|---|---|---|
| D-*a*Thr | 0.98 | 0.11 | 0.54 | n/a | 171.8 |
| D-Hse | 0.98 | 0.11 | 0.54 | 0.00 | 172.8 |
| D-Thr | 0.98 | 0.11 | 0.54 | 0.00 | 174.1 |
| L-Hse | 1.34 | 0.04 | 0.90 | 2.83 | 172.8 |
| L-*a*Thr | 1.45 | 0.04 | 1.01 | 1.62 | 171.8 |
| L-Thr | 1.52 | 0.06 | 1.08 | 0.83 | 174.1 |

**Table S8.** Chromatographic performance evaluation of the threonine isomer mixture consisting of DL-threonine (Thr) and DL-*allo*-threonine (*a*Thr). Tandem column: QN-AX + ZWIX(+) prototype core shell columns (3.0x50 mm, 2.7µm, respectively). Same LC conditions as in Figure S6.

| AQC-AA | RT [min] | $w_{1/2}$ [min] | $t'_R$ [min] | $R_s$ | $^{DT}CCS_{N2}$ [Å²] |
|---|---|---|---|---|---|
| D-Thr | 0.94 | 0.04 | 0.5 | n/a | 174.1 |
| D-*a*Thr | 1.03 | 0.05 | 0.59 | 1.18 | 171.8 |
| L-*a*Thr | 1.45 | 0.05 | 1.01 | 4.96 | 171.8 |
| L-Thr | 1.50 | 0.06 | 1.06 | 0.54 | 174.1 |

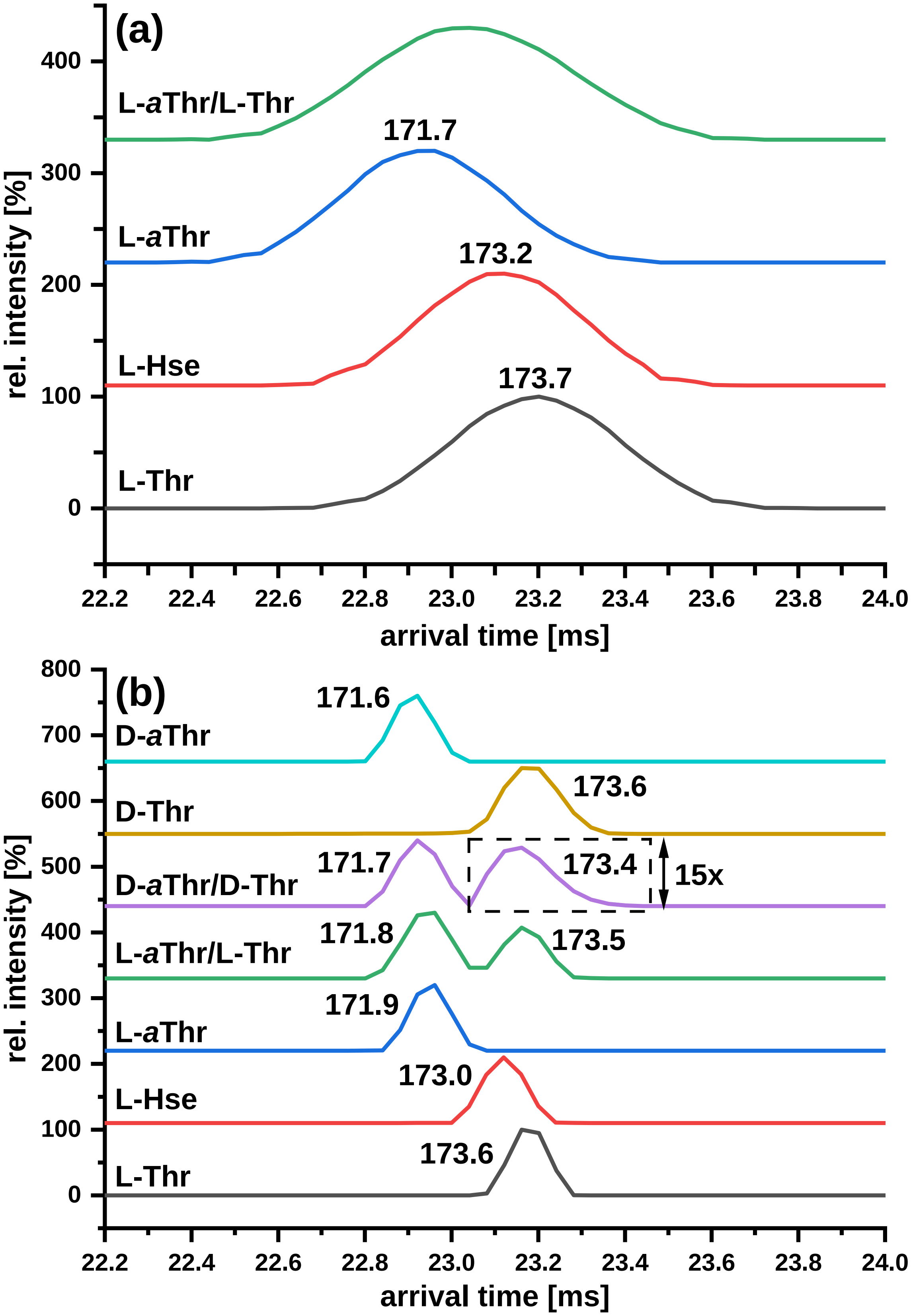


**Figure S7.** Standard arrival time spectra (a) and high-resolution demultiplexed arrival time spectra (b). The peak labels are the corresponding $^{DT}CCS_{N2}$ values [Å²]. The broader IM peak observed for D-Thr following HRdm is due to the low signal intensity of this compound in the examples shown. Assessment of higher D-Thr concentrations or additional fine-tuning of HRdm settings provide the same peak width and arrival time as L-Thr.

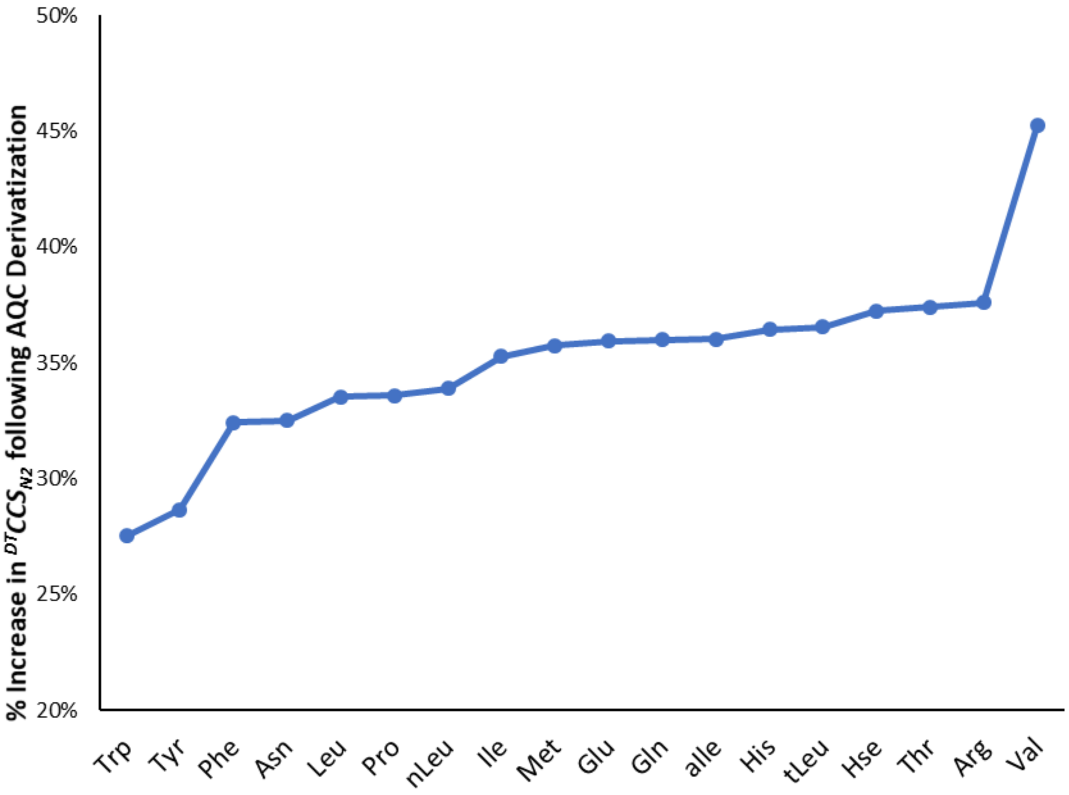


**Figure S8.** Impact of AQC derivatization on $^{DT}CCS_{N2}$ in comparison to corresponding protonated amino acids. Data for underivatized amino acids retrieved from the CCS Compendium (https://mcleanresearchgroup.shinyapps.io/CCS-Compendium)

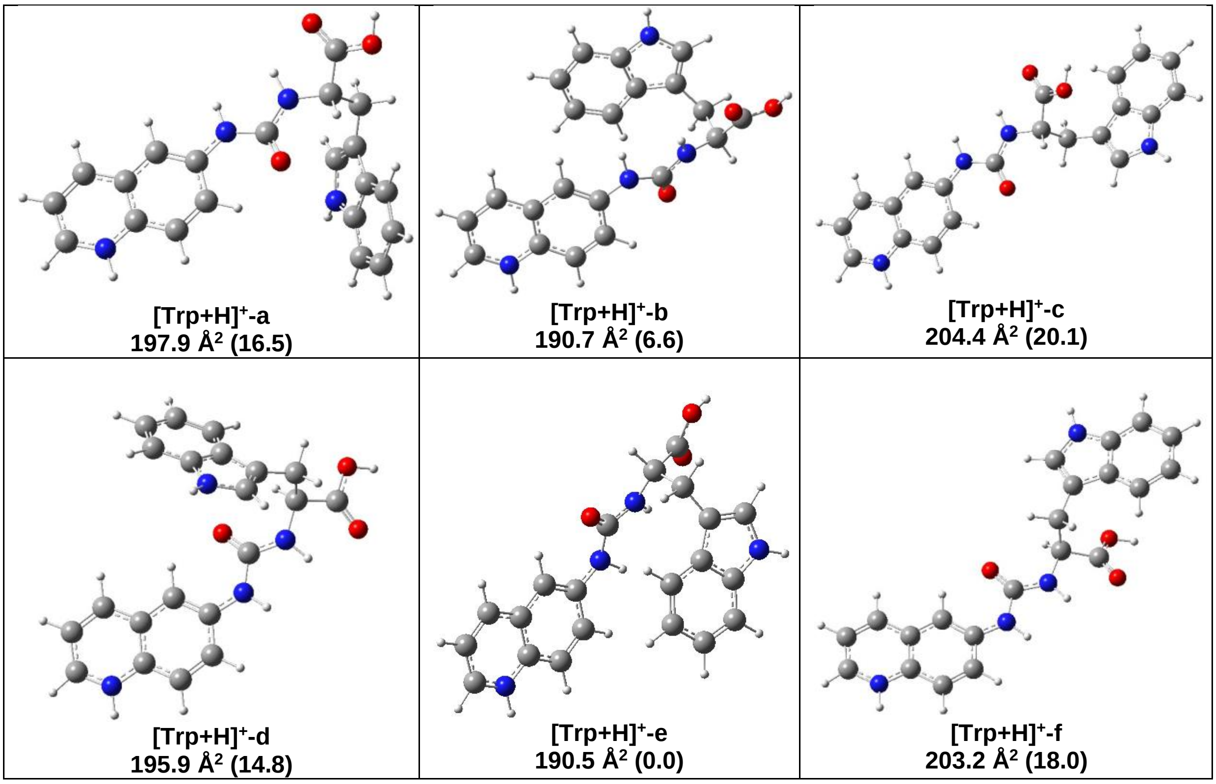


**Figure S9**. Optimized structures of different conformers of $[Trp+H]^+$ in the gas phase and their calculated $CCS_{N2}$ values. The numbers in parenthesis are the relative Gibbs free energies in kJ $mol^{-1}$. The calculated $CCS_{N2}$ values of the most stable isomers **b** and **e** (190.7 and 190.5 $Å^2$) are in agreement with the experimental $^{DT}CCS_{N2}$ (191.4 $Å^2$) indicating the twisted structure of $[Trp+H]^+$ in the gas phase because of a π-π interaction.

**Table S9**. The calculated $CCS_{N2}$ values and relative Gibbs free energies for the conformers of $[Trp+H]^+$. $^{DT}CCS_{N2}$ = 191.4 $Å^2$.

| **$[M+H]^+$** | **$\Delta G$ (kJ $mol^{-1}$)** | **$CCS_{N2}$ ($Å^2$)** |
|---|---|---|
| $[Trp+H]^+$-a | 16.5 | 197.9 |
| $[Trp+H]^+$-b | 5.6 | 190.7 |
| $[Trp+H]^+$-c | 20.1 | 204.4 |
| $[Trp+H]^+$-d | 14.8 | 195.9 |
| **$[Trp+H]^+$-e** | **0.0** | **190.5** |
| $[Trp+H]^+$-f | 18.0 | 203.2 |

**Table S10**. Calculation of the difference in the collisional cross section (CCS) of leucine and threonine isomers.

| **AQC-AA (A)** | **$^{DT}CCS_{N2}$ (A) [$Å^2$]** | **AA-AQC (B)** | **$^{DT}CCS_{N2}$ (B) [$Å^2$]** | **$\Delta^{DT}CCS_{N2}$ (A,B) [%]** |
|---|---|---|---|---|
| Ile | 180.9 | Leu | 180.9 | 0.00 |
| Ile | 180.9 | *a*Ile | 180.9 | 0.00 |
| Ile | 180.9 | *t*Leu | 181.6 | 0.39 |
| Ile | 180.9 | *n*Leu | 182.1 | 0.66 |
| Leu | 180.9 | *a*Ile | 180.9 | 0.00 |
| Leu | 180.9 | *t*Leu | 181.6 | 0.39 |
| Leu | 180.9 | *n*Leu | 182.1 | 0.66 |
| *a*Ile | 180.9 | *t*Leu | 181.6 | 0.39 |
| *a*Ile | 180.9 | *n*Leu | 182.1 | 0.66 |
| *t*Leu | 181.6 | *n*Leu | 182.1 | 0.28 |
| *a*Thr | 171.8 | Hse | 172.8 | 0.58 |
| *a*Thr | 171.8 | Thr | 174.1 | 1.34 |
| Hse | 172.8 | Thr | 174.1 | 0.75 |

| | | |
|---|---|---|
| 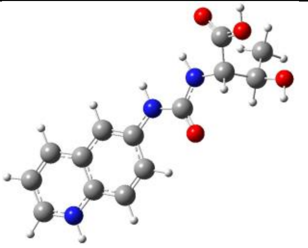<br>**[Thr+H]+-a**<br>**174.9 Å$^2$ (17.3)** | 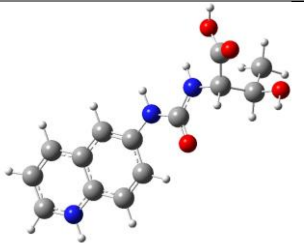<br>**[Thr+H]+-b**<br>**175.1 Å$^2$ (36.2)** | 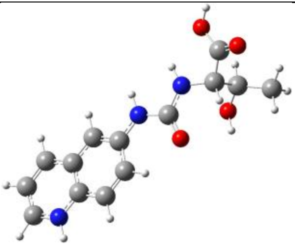<br>**[Thr+H]+-c**<br>**174.8 Å$^2$ (17.1)** |
| 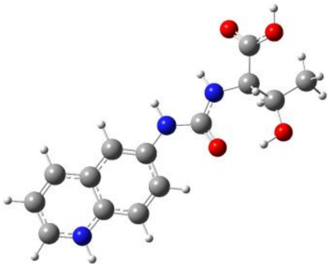<br>**[Thr+H]+-d**<br>**173.5 Å$^2$ (25.3)** | 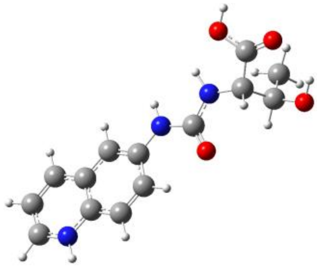<br>**[Thr+H]+-e**<br>**174.1 Å$^2$ (19.2)** | 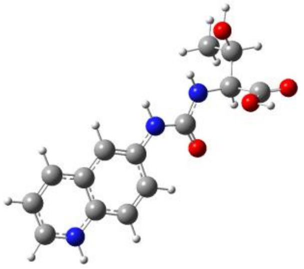<br>**[Thr+H]+-f**<br>**175.9 Å$^2$ (9.7)** |
| 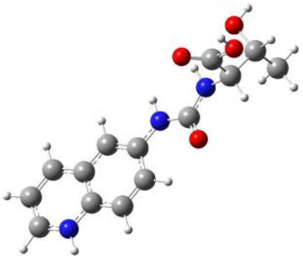<br>**[Thr+H]+-g**<br>**174.2 Å$^2$ (0.0)** | 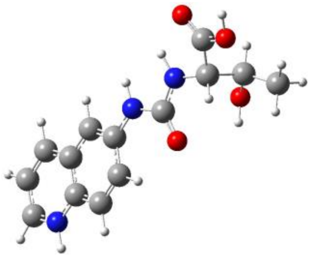<br>**[Thr+H]+-h**<br>**172.5 Å$^2$ (16.5)** | 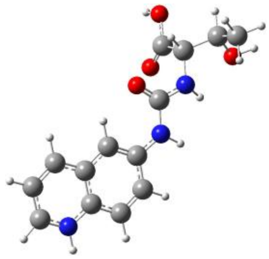<br>**[Thr+H]+-i**<br>**173.9 Å$^2$ (0.3)** |
| 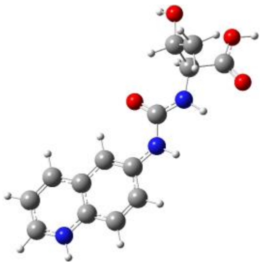<br>**[Thr+H]+-j**<br>**175.2 Å$^2$ (13.9)** | 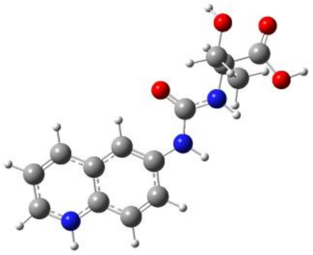<br>**[Thr+H]+-k**<br>**175.5 Å$^2$ (28.2)** | 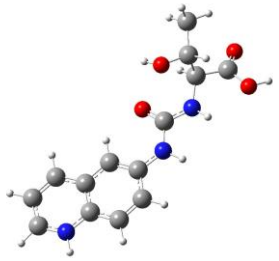<br>**[Thr+H]+-l**<br>**174.1 Å$^2$ (19.4)** |
| 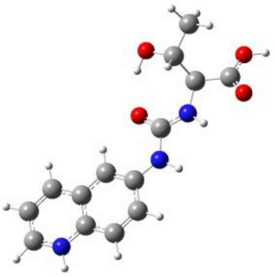<br>**[Thr+H]+-m**<br>**173.3 Å$^2$ (20.7)** | 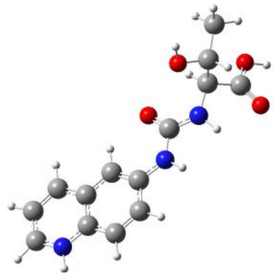<br>**[Thr+H]+-n**<br>**173.9 Å$^2$ (9.9)** | 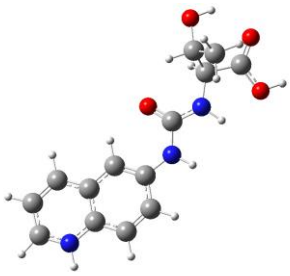<br>**[Thr+H]+-o**<br>**175.0 Å$^2$ (20.1)** |

**Figure S10.** Optimized structures of different conformers of [Thr+H]$^+$ in the gas phase and their calculated $CCS_{N2}$ values. The numbers in parenthesis are the relative Gibbs free energies in kJ mol$^{-1}$.

**Table S11.** The calculated $CCS_{N2}$ values and relative Gibbs free energies for the conformers of $[Thr+H]^+$. $^{DT}CCS_{N2}$ = 174.1 Å².

| $[M+H]^+$ | ∆G (kJ mol$^{-1}$) | $CCS_{N2}$ (Å$^2$) |
|---|---|---|
| $[Thr+H]^+$-a | 17.3 | 174.9 |
| $[Thr+H]^+$-b | 36.2 | 175.1 |
| $[Thr+H]^+$-c | 17.1 | 174.8 |
| $[Thr+H]^+$-d | 25.3 | 173.5 |
| $[Thr+H]^+$-e | 19.2 | 174.1 |
| $[Thr+H]^+$-f | 9.7 | 175.9 |
| **$[Thr+H]^+$-g** | **0.0** | **174.2** |
| $[Thr+H]^+$-h | 16.5 | 172.5 |
| $[Thr+H]^+$-i | 0.3 | 173.9 |
| $[Thr+H]^+$-j | 13.9 | 175.2 |
| $[Thr+H]^+$-k | 28.2 | 175.5 |
| $[Thr+H]^+$-l | 19.4 | 174.1 |
| $[Thr+H]^+$-m | 20.7 | 173.3 |
| $[Thr+H]^+$-n | 9.9 | 173.9 |
| $[Thr+H]^+$-o | 20.1 | 175.0 |

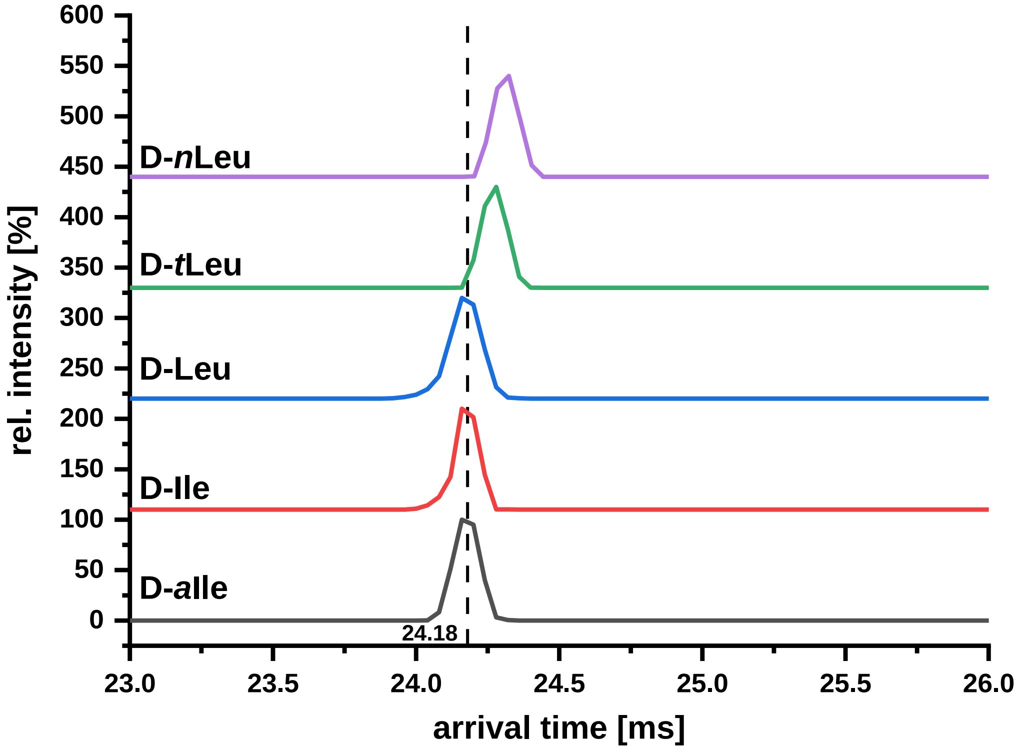


**Figure S11.** Arrival time spectra of single D-leucine isomers standards after application of high-resolution de-multiplexing. Expected Leu isomers in real samples cannot be resolved in complex mixtures using IM with HRdm.

| | | |
|---|---|---|
| 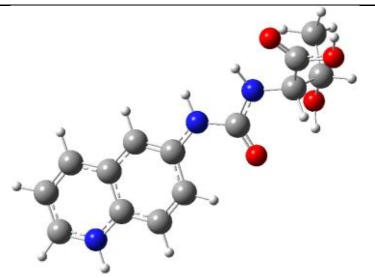<br>**[aThr+H]$^+$-a**<br>**172.9 Å$^2$ (5.6)** | 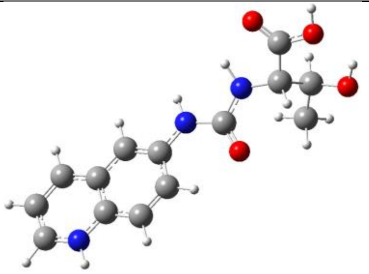<br>**[aThr+H]$^+$-b**<br>**172.2 Å$^2$ (11.8)** | 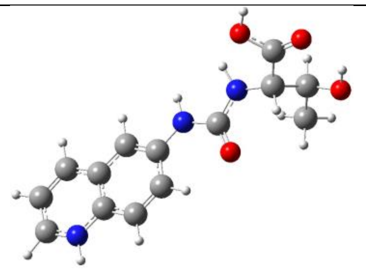<br>**[aThr+H]$^+$-c**<br>**174.4 Å$^2$ (21.9)** |
| 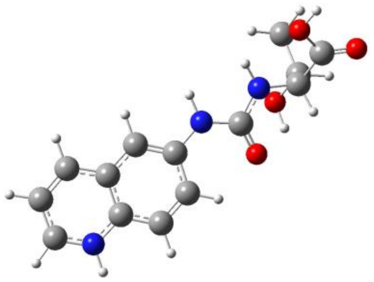<br>**[aThr+H]$^+$-d**<br>**176.0 Å$^2$ (17.5)** | 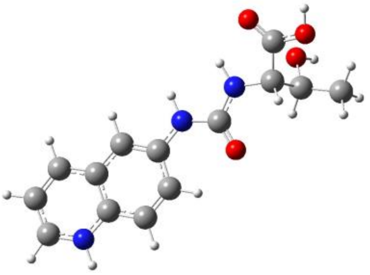<br>**[aThr+H]$^+$-e**<br>**173.2 Å$^2$ (7.7)** | 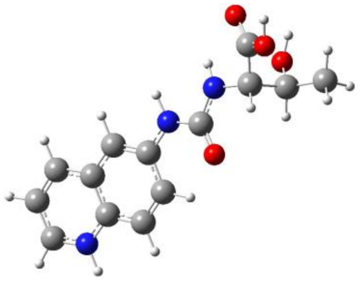<br>**[aThr+H]$^+$-f**<br>**173.7 Å$^2$ (6.3)** |
| 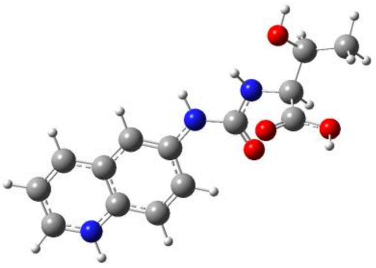<br>**[aThr+H]$^+$-g**<br>**171.2 Å$^2$ (4.9)** | 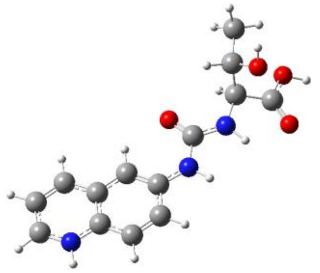<br>**[aThr+H]$^+$-h**<br>**171.7 Å$^2$ (1.0)** | 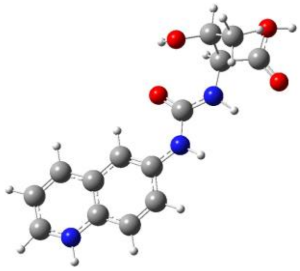<br>**[aThr+H]$^+$-i**<br>**171.8 Å$^2$ (1.8)** |
| 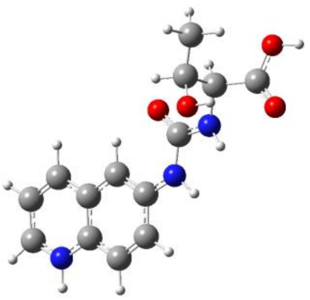<br>**[aThr+H]$^+$-j**<br>**174.2 Å$^2$ (2.6)** | 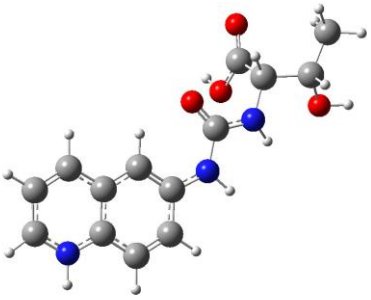<br>**[aThr+H]$^+$-k**<br>**174.9 Å$^2$ (5.8)** | 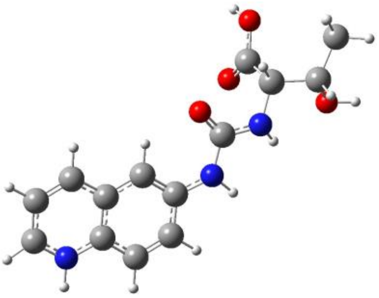<br>**[aThr+H]$^+$-l**<br>**172.2 Å$^2$ (0.0)** |

**Figure S12.** Optimized structures of different conformers of [aThr+H]$^+$ in the gas phase and their calculated $CCS_{N2}$ values. The numbers in parenthesis are the relative Gibbs free energies in kJ mol$^{-1}$.

**Table S12.** The calculated $CCS_{N2}$ values and relative Gibbs free energies for the conformers of $[aThr+H]^+$. Experimental $^{DT}CCS_{N2}$ = 171.8 Å².

| **[M+H]⁺** | **ΔG (kJ mol⁻¹)** | **$CCS_{N2}$ (Å²)** |
|---|---|---|
| $[aThr+H]^+$-a | 5.6 | 172.9 |
| $[aThr+H]^+$-b | 11.8 | 172.2 |
| $[aThr+H]^+$-c | 21.9 | 174.4 |
| $[aThr+H]^+$-d | 17.5 | 176.0 |
| $[aThr+H]^+$-e | 7.7 | 173.2 |
| $[aThr+H]^+$-f | 6.3 | 173.7 |
| $[aThr+H]^+$-g | 4.9 | 171.2 |
| $[aThr+H]^+$-h | 1.0 | 171.7 |
| $[aThr+H]^+$-i | 1.8 | 171.8 |
| $[aThr+H]^+$-j | 2.6 | 174.2 |
| $[aThr+H]^+$-k | 5.8 | 174.9 |
| **$[aThr+H]^+$-l** | **0.0** | **172.2** |

| | | |
|---|---|---|
| 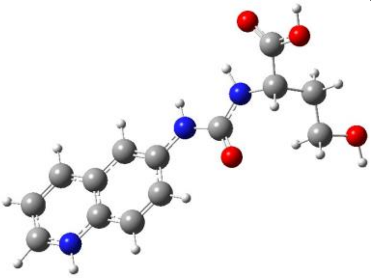<br>**$[HSe+H]^+$-a**<br>**175.4 Å$^2$ (19.4)** | 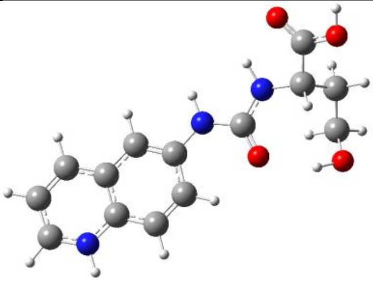<br>**$[HSe+H]^+$-b**<br>**173.1 Å$^2$ (7.6)** | 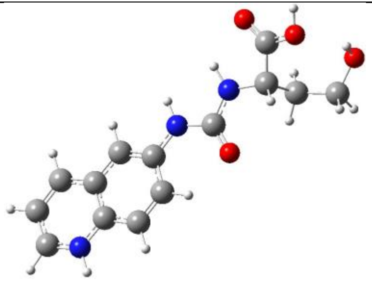<br>**$[HSe+H]^+$-c**<br>**173.4 Å$^2$ (21.2)** |
| 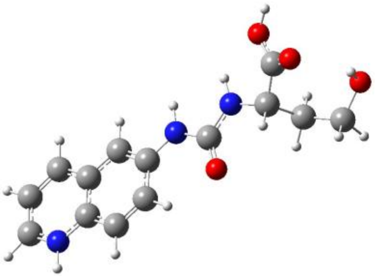<br>**$[HSe+H]^+$-d**<br>**174.6 Å$^2$ (21.7)** | 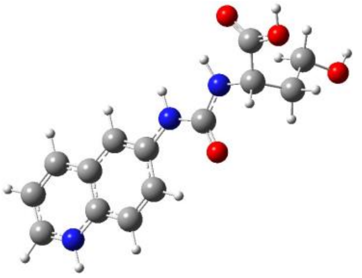<br>**$[HSe+H]^+$-e**<br>**175.6 Å$^2$ (16.3)** | 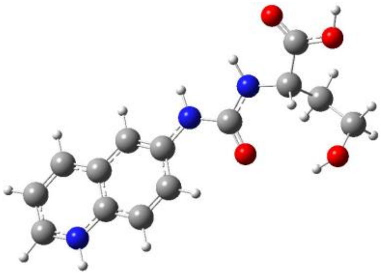<br>**$[HSe+H]^+$-f**<br>**172.8 Å$^2$ (18.3)** |
| 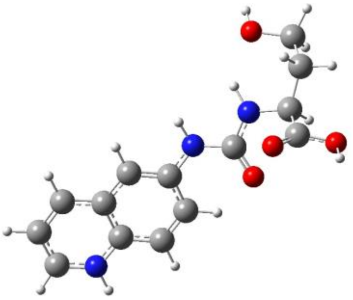<br>**$[HSe+H]^+$-g**<br>**172.3 Å$^2$ (4.3)** | 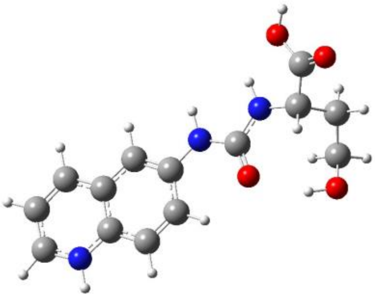<br>**$[HSe+H]^+$-h**<br>**175.4 Å$^2$ (19.6)** | 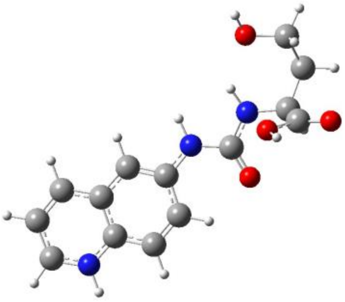<br>**$[HSe+H]^+$-i**<br>**173.4 Å$^2$ (0.6)** |
| 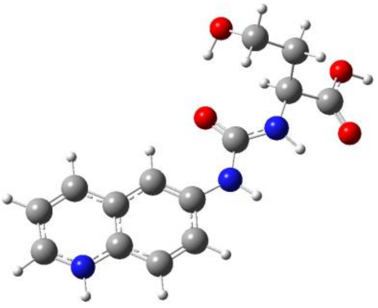<br>**$[HSe+H]^+$-j**<br>**174.0 Å$^2$ (4.1)** | 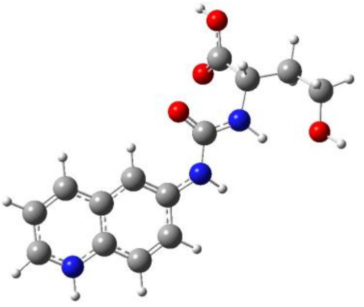<br>**$[HSe+H]^+$-k**<br>**172.5 Å$^2$ (0.0)** | 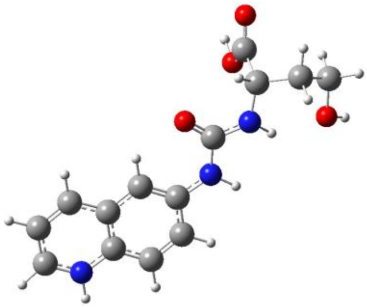<br>**$[HSe+H]^+$-l**<br>**174.2 Å$^2$ (1.1)** |

**Figure S13.** Optimized structures of different conformers of $[HSe+H]^+$ in the gas phase and their calculated $CCS_{N2}$ values. The numbers in parenthesis are the relative Gibbs free energies in kJ mol$^{-1}$.

**Table S13.** The calculated $CCS_{N2}$ values and relative Gibbs free energies for the conformers of $[HSe+H]^+$. Experimental $^{DT}CCS_{N2}$ = 172.8 Å².

| **$[M+H]^+$** | **ΔG (kJ $mol^{-1}$)** | **$CCS_{N2}$ (Å$^2$)** |
|---|---|---|
| $[HSe+H]^+$-a | 19.4 | 175.4 |
| $[HSe+H]^+$-b | 7.6 | 173.1 |
| $[HSe+H]^+$-c | 21.2 | 173.4 |
| $[HSe+H]^+$-d | 21.7 | 174.6 |
| $[HSe+H]^+$-e | 16.3 | 175.6 |
| $[HSe+H]^+$-f | 18.3 | 172.8 |
| $[HSe+H]^+$-g | 4.3 | 172.3 |
| $[HSe+H]^+$-h | 19.6 | 175.4 |
| $[HSe+H]^+$-i | 0.6 | 173.4 |
| $[HSe+H]^+$-j | 4.1 | 174.0 |
| **$[HSe+H]^+$-k** | **0.0** | **172.5** |
| $[HSe+H]^+$-l | 1.1 | 174.2 |

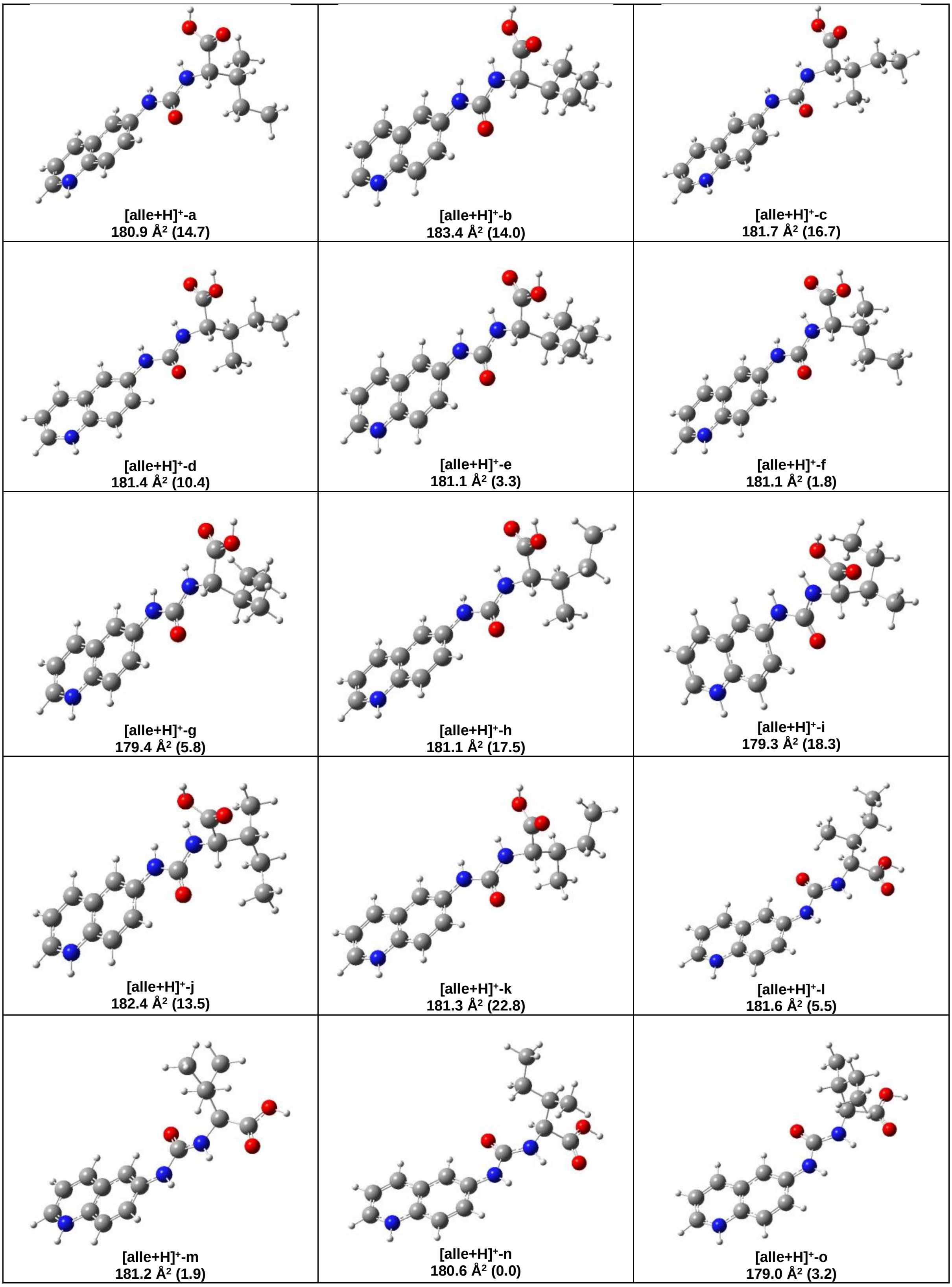


**Figure S14.** Optimized structures of different conformers of [alle+H]$^+$ in the gas phase and their calculated $CCS_{N2}$ values. The numbers in parenthesis are the relative Gibbs free energies in kJ mol$^{-1}$.

**Table S14.** The calculated *$CCS_{N2}$* values and relative Gibbs free energies for the conformers of $[alle+H]^+$. Experimental $^{DT}CCS_{N2}$ = 180.9 Å$^2$.

| **$[M+H]^+$** | **ΔG (kJ mol$^{-1}$)** | **$CCS_{N2}$ (Å$^2$)** |
|---|---|---|
| $[alle+H]^+$-a | 14.7 | 180.9 |
| $[alle+H]^+$-b | 14.0 | 183.4 |
| $[alle+H]^+$-c | 16.7 | 181.7 |
| $[alle+H]^+$-d | 10.4 | 181.4 |
| $[alle+H]^+$-e | 3.3 | 181.1 |
| $[alle+H]^+$-f | 1.8 | 181.1 |
| $[alle+H]^+$-g | 5.8 | 179.4 |
| $[alle+H]^+$-h | 17.5 | 181.1 |
| $[alle+H]^+$-i | 18.3 | 179.3 |
| $[alle+H]^+$-j | 13.5 | 182.4 |
| $[alle+H]^+$-k | 22.8 | 181.3 |
| $[alle+H]^+$-l | 5.5 | 181.6 |
| $[alle+H]^+$-m | 1.9 | 181.2 |
| **$[alle+H]^+$-n** | **0.0** | **180.6** |
| $[alle+H]^+$-o | 3.2 | 179.0 |

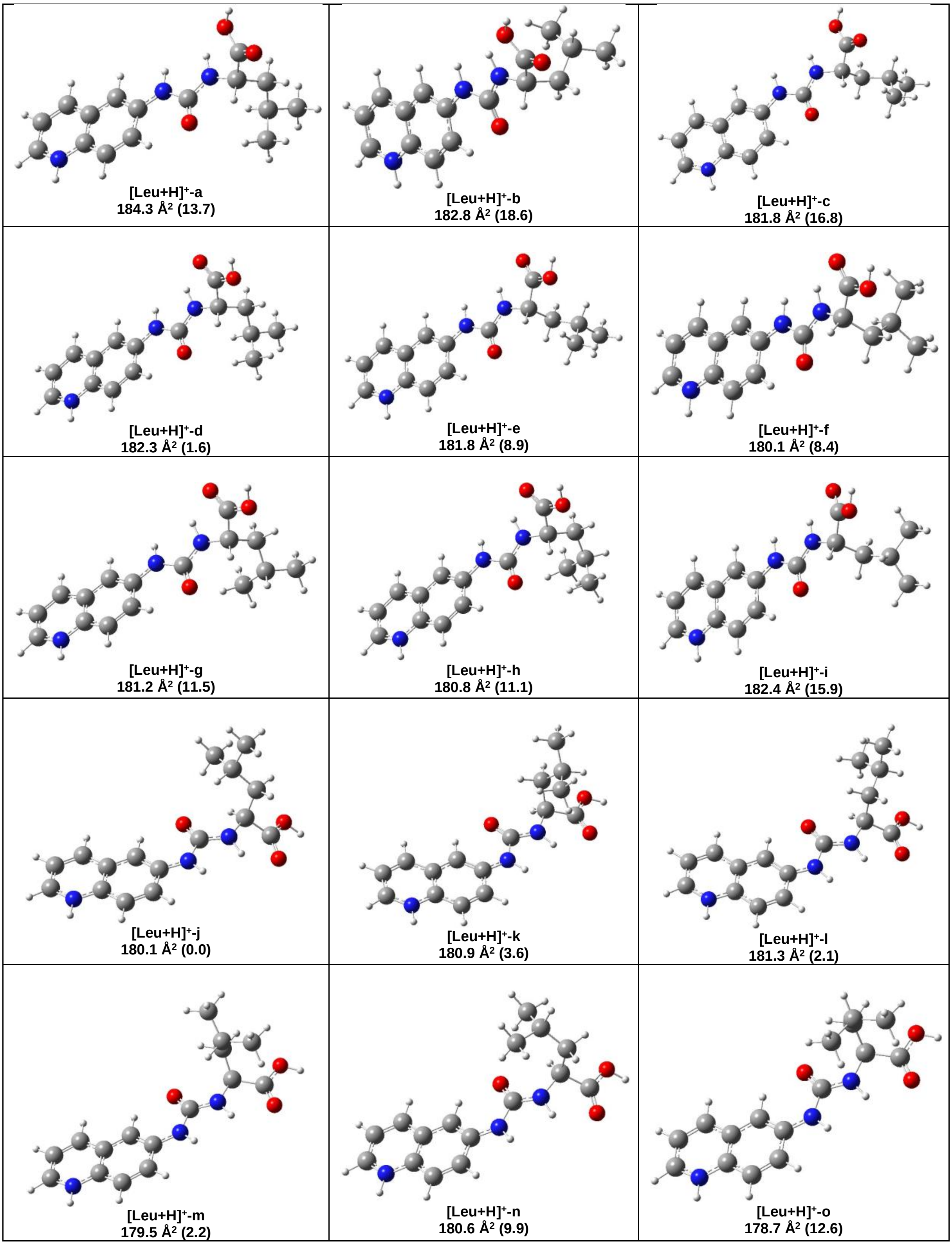


**Figure S15.** Optimized structures of different conformers of $[Leu+H]^+$ in the gas phase and their calculated $CCS_{N2}$ values. The numbers in parenthesis are the relative Gibbs free energies in kJ mol$^{-1}$.

**Table S15**.The calculated *$CCS_{N2}$* values and relative Gibbs free energies for the conformers of $[Leu+H]^+$. Experimental $^{DT}CCS_{N2}$ = 180.9 Å².

| **$[M+H]^+$** | **ΔG (kJ mol$^{-1}$)** | **$CCS_{N2}$ (Å$^2$)** |
|---|---|---|
| $[Leu+H]^+$-a | 13.7 | 184.3 |
| $[Leu+H]^+$-b | 18.6 | 182.8 |
| $[Leu+H]^+$-c | 16.8 | 181.8 |
| $[Leu+H]^+$-d | 1.6 | 182.3 |
| $[Leu+H]^+$-e | 8.9 | 181.8 |
| $[Leu+H]^+$-f | 8.4 | 180.1 |
| $[Leu+H]^+$-g | 11.5 | 181.2 |
| $[Leu+H]^+$-h | 11.1 | 180.8 |
| $[Leu+H]^+$-i | 15.9 | 182.4 |
| **$[Leu+H]^+$-j** | **0.0** | **180.1** |
| $[Leu+H]^+$-k | 3.6 | 180.9 |
| $[Leu+H]^+$-l | 2.1 | 181.3 |
| $[Leu+H]^+$-m | 2.2 | 179.5 |
| $[Leu+H]^+$-n | 9.9 | 180.6 |
| $[Leu+H]^+$-o | 12.6 | 178.7 |

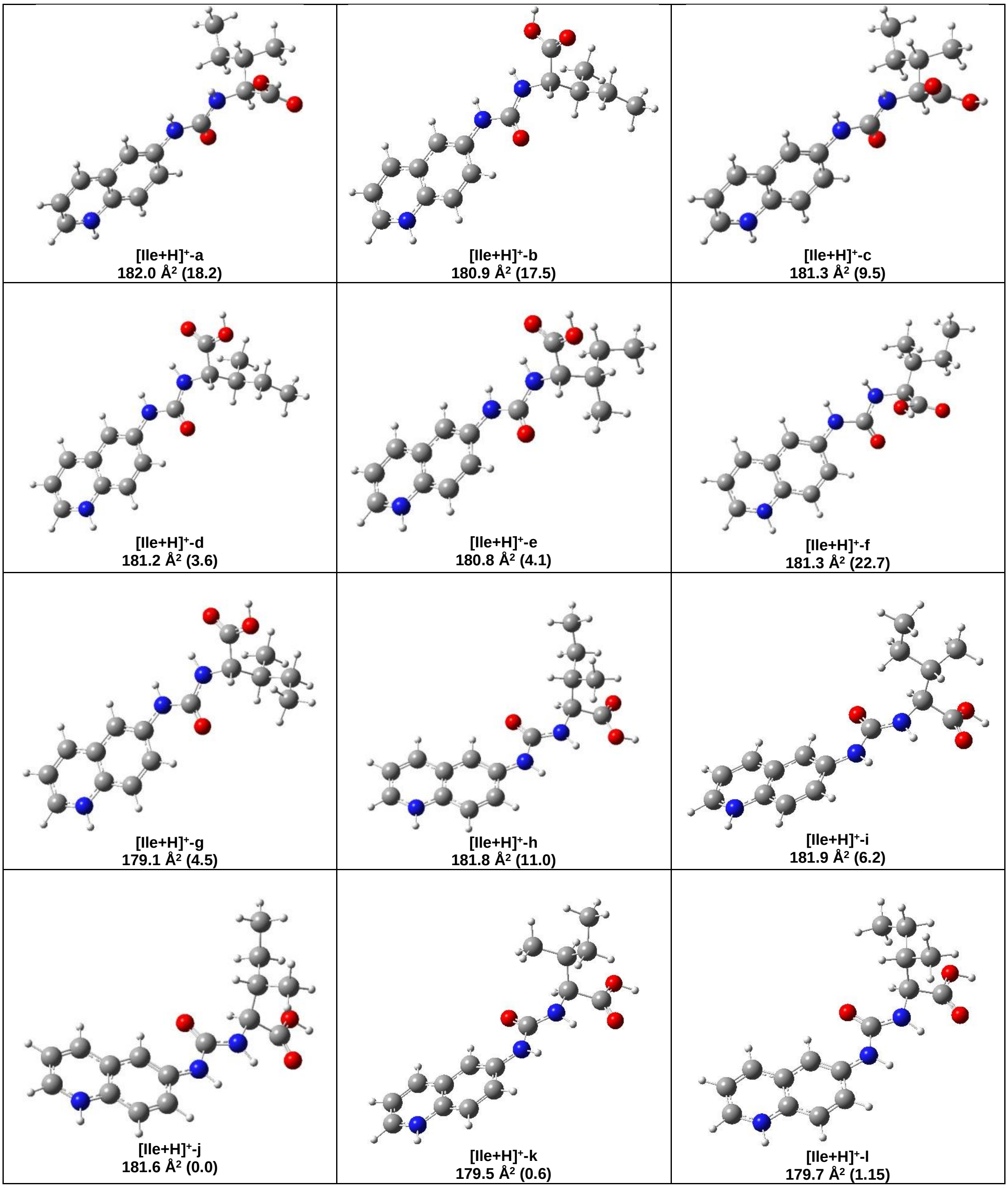


**Figure S16.** Optimized structures of different conformers of [Ile+H]$^+$ in the gas phase and their calculated $CCS_{N2}$ values. The numbers in parenthesis are the relative Gibbs free energies in kJ mol$^{-1}$.

**Table S16.** The calculated $CCS_{N2}$ values and relative Gibbs free energies for the conformers of $[Ile+H]^+$. Experimental $^{DT}CCS_{N2}$ = 180.9 Å².

| $[M+H]^+$ | ΔG (kJ mol$^{-1}$) | $CCS_{N2}$ (Å$^2$) |
|---|---|---|
| $[Ile+H]^+$-a | 18.2 | 182.0 |
| $[Ile+H]^+$-b | 17.5 | 180.9 |
| $[Ile+H]^+$-c | 9.5 | 181.3 |
| $[Ile+H]^+$-d | 3.6 | 181.2 |
| $[Ile+H]^+$-e | 4.1 | 180.8 |
| $[Ile+H]^+$-f | 22.7 | 181.3 |
| $[Ile+H]^+$-g | 4.5 | 179.1 |
| $[Ile+H]^+$-h | 11.0 | 181.8 |
| $[Ile+H]^+$-i | 6.2 | 181.9 |
| **$[Ile+H]^+$-j** | **0.0** | **181.6** |
| $[Ile+H]^+$-k | 0.6 | 179.5 |
| $[Ile+H]^+$-l | 1.5 | 179.7 |

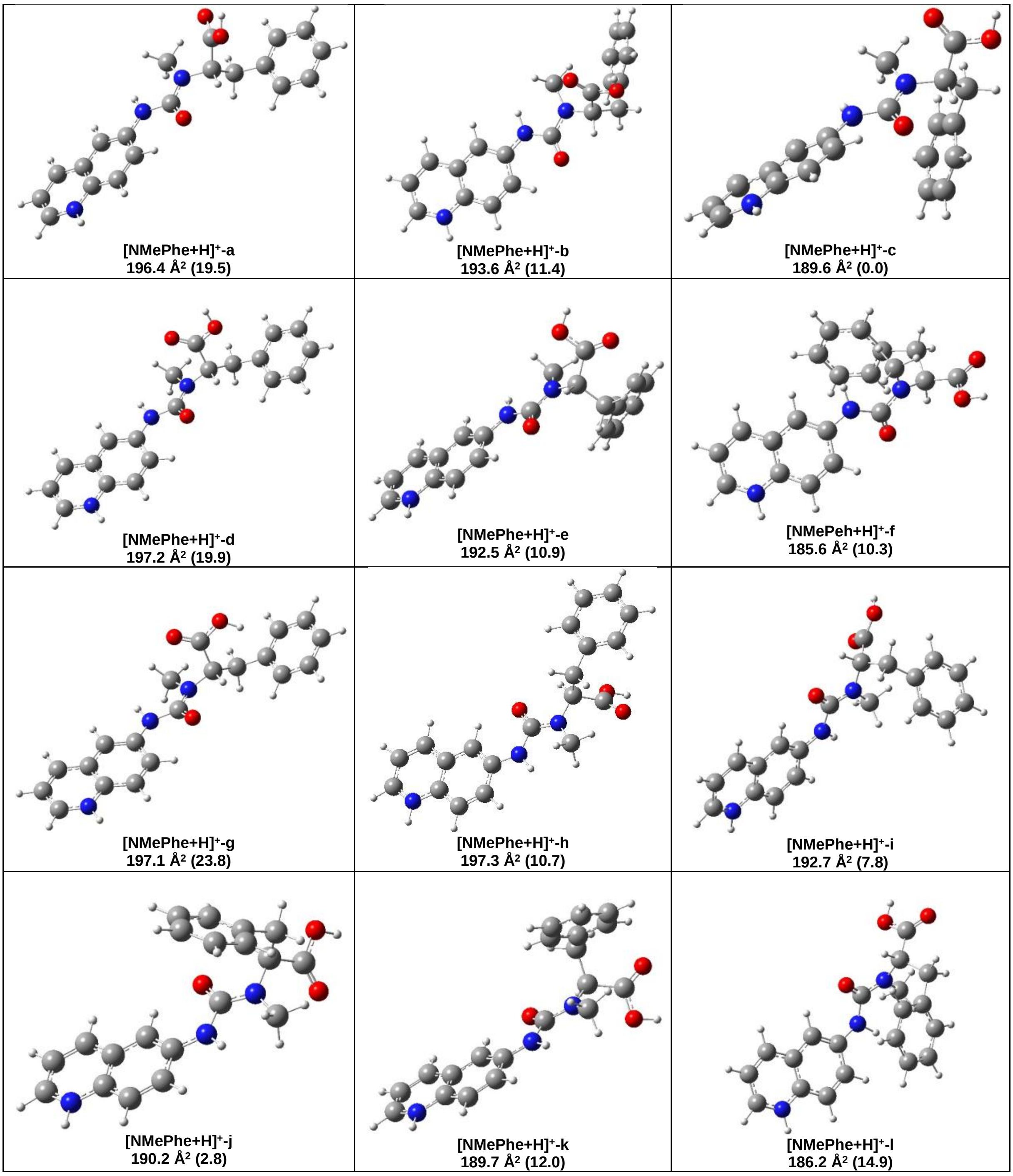


**Figure S17.** Optimized structures of different conformers of *N*-methylphenylalanine $[NMePhe+H]^+$ in the gas phase and their calculated $CCS_{N2}$ values. The numbers in parenthesis are the relative Gibbs free energies in kJ mol$^{-1}$.

**Table S17.** The calculated $CCS_{N2}$ values and relative Gibbs free energies for the conformers of *N*-methylphenylalanine $[NMePhe+H]^+$.

| $[M+H]^+$ | $\Delta$G (kJ mol$^{-1}$) | $CCS_{N2}$ (Å$^2$) |
|---|---|---|
| $[NMePhe+H]^+$-a | 19.5 | 196.4 |
| $[NMePhe+H]^+$-b | 11.4 | 193.6 |
| **$[NMePhe+H]^+$-c** | **0.0** | **189.6** |
| $[NMePhe+H]^+$-d | 19.9 | 197.2 |
| $[NMePhe+H]^+$-e | 10.9 | 192.5 |
| $[NMePhe+H]^+$-f | 10.3 | 185.6 |
| $[NMePhe+H]^+$-g | 23.8 | 197.1 |
| $[NMePhe+H]^+$-h | 10.7 | 197.3 |
| $[NMePhe+H]^+$-i | 7.8 | 192.7 |
| $[NMePhe+H]^+$-j | 2.8 | 190.2 |
| $[NMePhe+H]^+$-k | 12.0 | 189.7 |
| $[NMePhe+H]^+$-l | 14.9 | 186.2 |

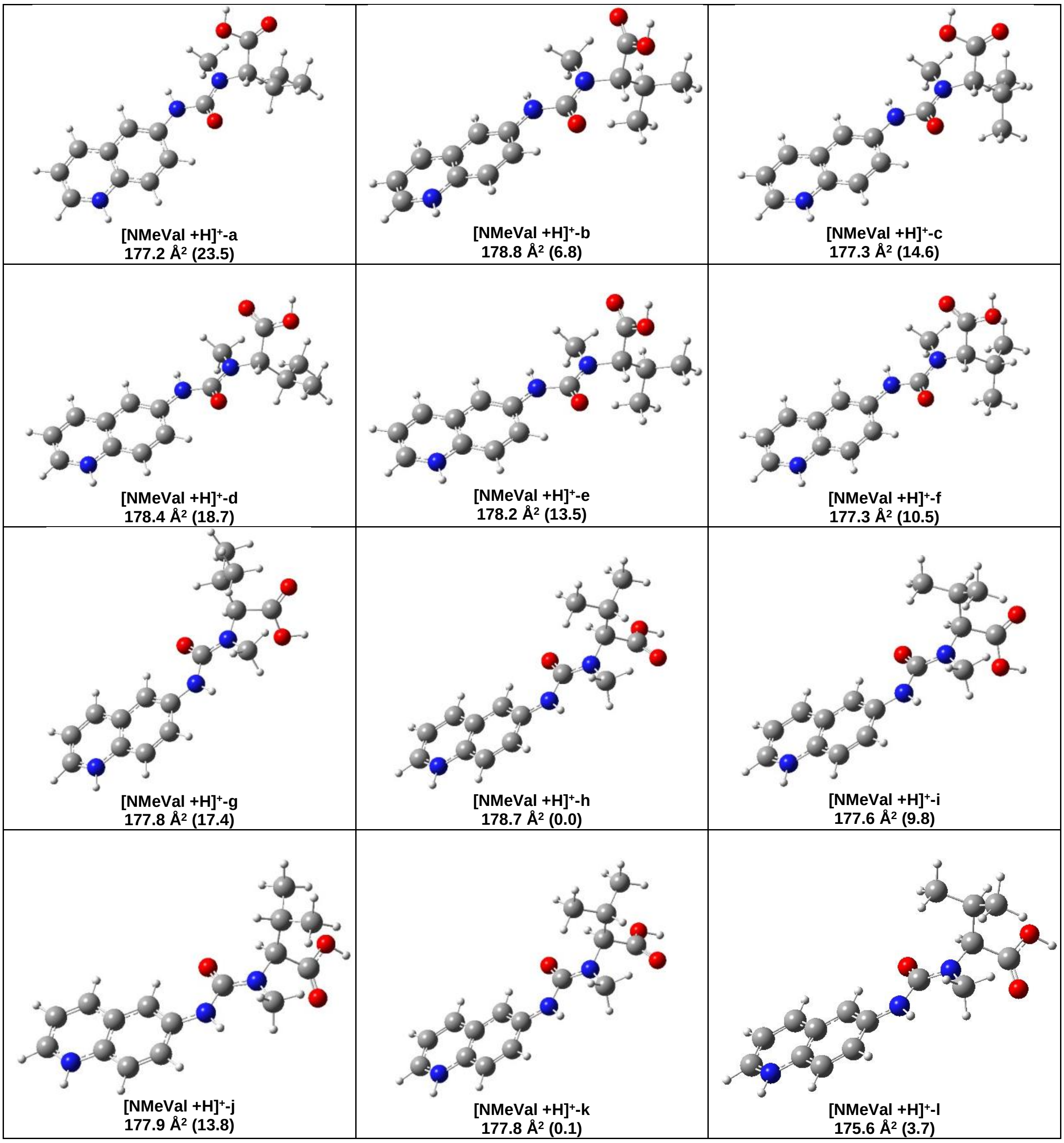


**Figure S18.** Optimized structures of different conformers of *N*-methylvaline [NMeVal+H]$^{+}$ in the gas phase and their calculated $CCS_{N2}$ values. The numbers in parenthesis are the relative Gibbs free energies in kJ mol$^{-1}$.

**Table S18.** The calculated $CCS_{N2}$ values and relative Gibbs free energies for the conformers of *N*-methylvaline $[NMeVal+H]^+$.

| $[M+H]^+$ | ΔG (kJ $mol^{-1}$) | $CCS_{N2}$ (Å$^2$) |
|---|---|---|
| $[NMeVal+H]^+$-a | 23.5 | 177.2 |
| $[NMeVal+H]^+$-b | 6.8 | 178.8 |
| $[NMeVal+H]^+$-c | 14.6 | 177.3 |
| $[NMeVal+H]^+$-d | 18.7 | 178.4 |
| $[NMeVal+H]^+$-e | 13.5 | 178.2 |
| $[NMeVal+H]^+$-f | 10.5 | 177.3 |
| $[NMeVal+H]^+$-g | 17.4 | 177.8 |
| **$[NMeVal+H]^+$-h** | **0.0** | **178.7** |
| $[NMeVal+H]^+$-i | 9.8 | 177.6 |
| $[NMeVal+H]^+$-j | 13.8 | 177.9 |
| $[NMeVal+H]^+$-k | 0.1 | 177.8 |
| $[NMeVal+H]^+$-l | 3.7 | 175.6 |

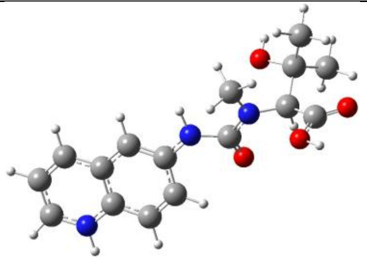

**[NMeValOH +H]+-a**
**178.4 Å² (14.0)**

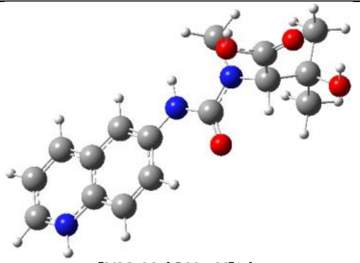

**[NMeValOH +H]+-b**
**182.2 Å² (17.6)**

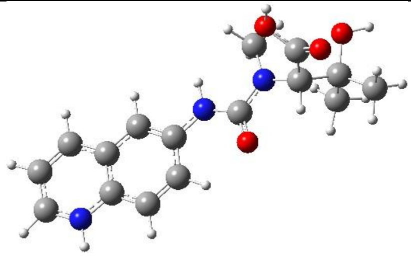

**[NMeValOH +H]+-c**
**181.3 Å² (12.8)**

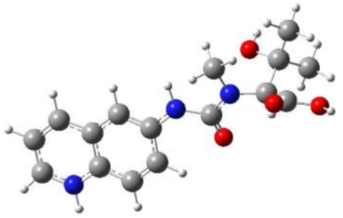

**[NMeValOH +H]+-d**
**180.5 Å² (10.0)**

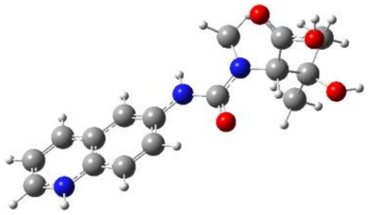

**[NMeValOH +H]+-e**
**181.6 Å² (25.1)**

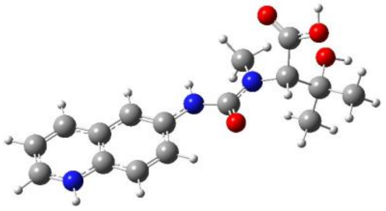

**[NMeValOH +H]+-f**
**180.3 Å² (0.6)**

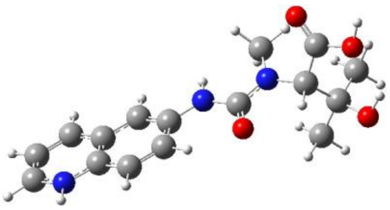

**[NMeValOH +H]+-g**
**181.9 Å² (14.8)**

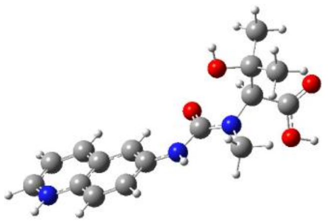

**[NMeValOH +H]+-h**
**179.7 Å² (11.2)**

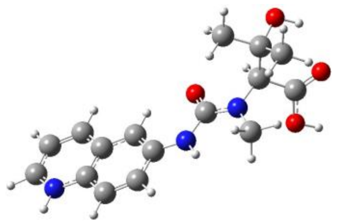

**[NMeValOH +H]+-i**
**179.7 Å² (4.8)**

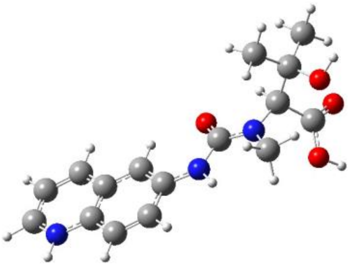

**[NMeValOH +H]+-j**
**181.5 Å² (2.6)**

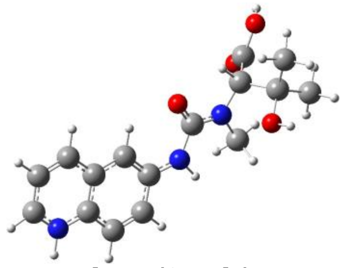

**[NMeValOH +H]+-k**
**178.4 Å² (8.9)**

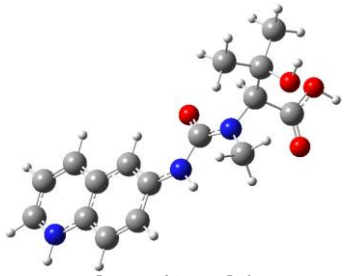

**[NMeValOH +H]+-l**
**178.8 Å² (0.0)**

**Figure S19.** Optimized structures of different conformers of 3-hydroxy-*N*-methylvaline [NMeValOH+H]+ in the gas phase and their calculated $CCS_{N2}$ values. The numbers in parenthesis are the relative Gibbs free energies in kJ mol$^{-1}$.

**Table S19**.The calculated $CCS_{N2}$ values and relative Gibbs free energies for the conformers of 3-hydroxy-*N*-methylvaline $[NMeValOH+H]^+$.

| **$[M+H]^+$** | **$\Delta G$ (kJ mol$^{-1}$)** | **$CCS_{N2}$ (Å$^2$)** |
|---|---|---|
| $[NMeValOH+H]^+$-a | 14.0 | 178.4 |
| $[NMeValOH+H]^+$-b | 17.6 | 182.2 |
| $[NMeValOH+H]^+$-c | 12.8 | 181.3 |
| $[NMeValOH+H]^+$-d | 10.0 | 180.5 |
| $[NMeValOH+H]^+$-e | 25.1 | 181.6 |
| $[NMeValOH+H]^+$-f | 0.6 | 180.3 |
| $[NMeValOH+H]^+$-g | 14.8 | 181.9 |
| $[NMeValOH+H]^+$-h | 11.2 | 179.7 |
| $[NMeValOH+H]^+$-i | 4.8 | 179.7 |
| $[NMeValOH+H]^+$-j | 2.6 | 181.5 |
| $[NMeValOH+H]^+$-k | 8.9 | 178.4 |
| **$[NMeValOH+H]^+$-l** | **0.0** | **178.8** |

**Table S20.** Comparison of the experimentally obtained and theoretically calculated CCS-values from the lipopeptide sample hydrolysate. The exact (calc.) *m/z* value of the protonated species is provided.

| AQC-AA | RT [min] | exp. $^{DT}CCS_{N2}$ [Å²] | calc. $^{DT}CCS_{N2}$ [Å²] | $\Delta^{DT}CCS_{N2}$ [%] | *m/z* | conformer with lowest energy |
|---|---|---|---|---|---|---|
| D-Asp | 2.19 | 175.2 | n/a | n/a | 304.093 | n/a |
| D-Glu | 1.7 | 178.2 | n/a | n/a | 318.108 | n/a |
| L-Glu | 1.84 | 178.2 | n/a | n/a | 318.108 | n/a |
| D-Ser | 1.13 | 168.8 | n/a | n/a | 276.098 | n/a |
| D-*a*Thr | 0.98 | 171.8 | 172.2 | 0.23 | 290.114 | I |
| L-Val | 1.15 | 175.6 | n/a | n/a | 288.134 | n/a |

**Table S21.** Comparison of experimentally obtained and theoretically calculated CCS-values from octreotide sample hydrolysate. The exact (calc.) *m/z* value of the protonated species is provided. Irregular peptide building block: Threoninol (Thr-ol).

| AQC-AA | RT [min] | exp. $^{DT}CCS_{N2}$ [Å²] | calc. $^{DT}CCS_{N2}$ [Å²] | $\Delta^{DT}CCS_{N2}$ [%] | *m/z* | conformer with lowest energy |
|---|---|---|---|---|---|---|
| D-Phe | 1.04 | 187.1 | n/a | n/a | 336.134 | n/a |
| L-Cys-IAA | 1.67 | 183.5 | n/a | n/a | 349.097 | n/a |
| L-Phe | 1.36 | 187.1 | n/a | n/a | 336.134 | n/a |
| D-Trp | 1.27 | 191.4 | n/a | n/a | 375.145 | n/a |
| L-Lys-bis-AQC | 1.38 | 209.4 | n/a | n/a | 487.209 | n/a |
| L-Thr | 0.98 | 174.1 | 174.2 | 0.06 | 290.114 | g |
| L-Thr-ol | 0.47 | 169.9 | n/a | n/a | 276.134 | n/a |

**Table S22.** Comparison of the experimentally obtained and theoretically calculated CCS-values from the aureobasidin A sample hydrolysate. The exact (calc.) *m/z* value of the protonated species is provided. Irregular amino acids and peptide building blocks, respectively: 3-hydroxy-*N*-methylvaline (NMeValOH), 2-hydroxy-3-methylpentanoic acid (OIle), *N*-methylvaline (NMeVal), *N*-methylphenylalanine (NMePhe).

| **AQC-AA** | **RT [min]** | **exp. $^{DT}CCS_{N2}$ [Å²]** | **calc. $^{DT}CCS_{N2}$ [Å²]** | **$\Delta^{DT}CCS_{N2}$ [%]** | ***m/z*** | **conformer with lowest energy** |
|---|---|---|---|---|---|---|
| L-Leu | 0.92 | 180.9 | 180.1 | -0.44 | 302.150 | j |
| L-NMeValOH | n/a | n/a | 178.8 | n/a | 318.124 | l |
| D-OIle | n/a | n/a | n/a | n/a | n/a | n/a |
| L-NMeVal[a] | 1.03 | 181 | 178.7 | -1.29 | 302.150 | h |
| L-Phe | 1.20 | 187.1 | n/a | n/a | 286.119 | n/a |
| L-NMePhe | 0.86 | 186.4 | 189.6 | 1.69 | 350. 150 | c |
| L-Pro | 0.91 | 168.6 | n/a | n/a | 286.119 | n/a |
| L-*a*Ile | 1.03 | 180.9 | 180.6 | -0.17 | 302.150 | n |

[a]: isobaric to Leu

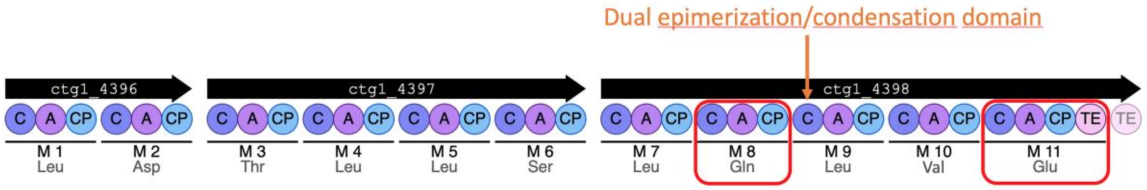


**Figure S20**. NRPS gene cluster of the investigated lipopeptide. Each module consists of a condensation (C), an adenylation (A) and a carrier protein (CP) domain. The adenylation domain recognizes and activates a specific amino acid and hands it over to the CP domain. C domains fuse two amino acids that are presented by CP domains. Finally, the formed peptide string is cleaved off the mega-enzyme with the help of a thioesterase domain (TE). The red boxes indicate modules 8 and 11, responsible for the recognition and activation of Gln and Glu, respectively. Dual epimerization/condensation domains are capable to convert the absolute configuration of the alpha-proton of the amino acid of the preceding module.

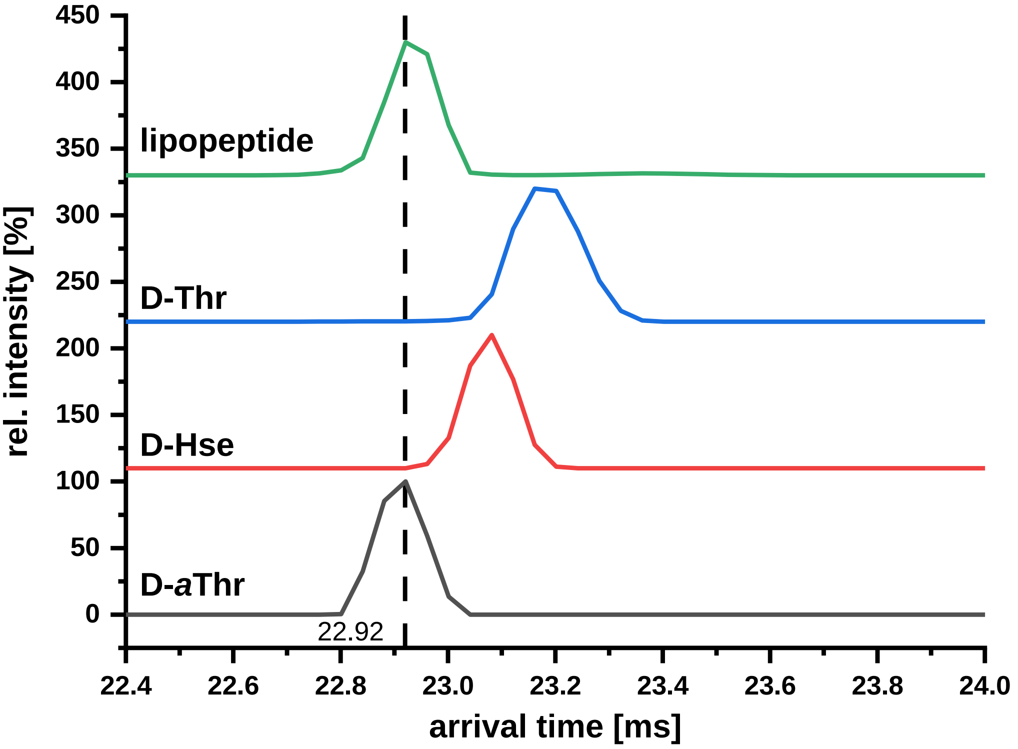


**Figure S21**. HRdm arrival time spectra of the lipopeptide hydrolysate sample and single D-Thr, D-Hse and D-*a*Thr standards. Same conditions as in Figure 5.

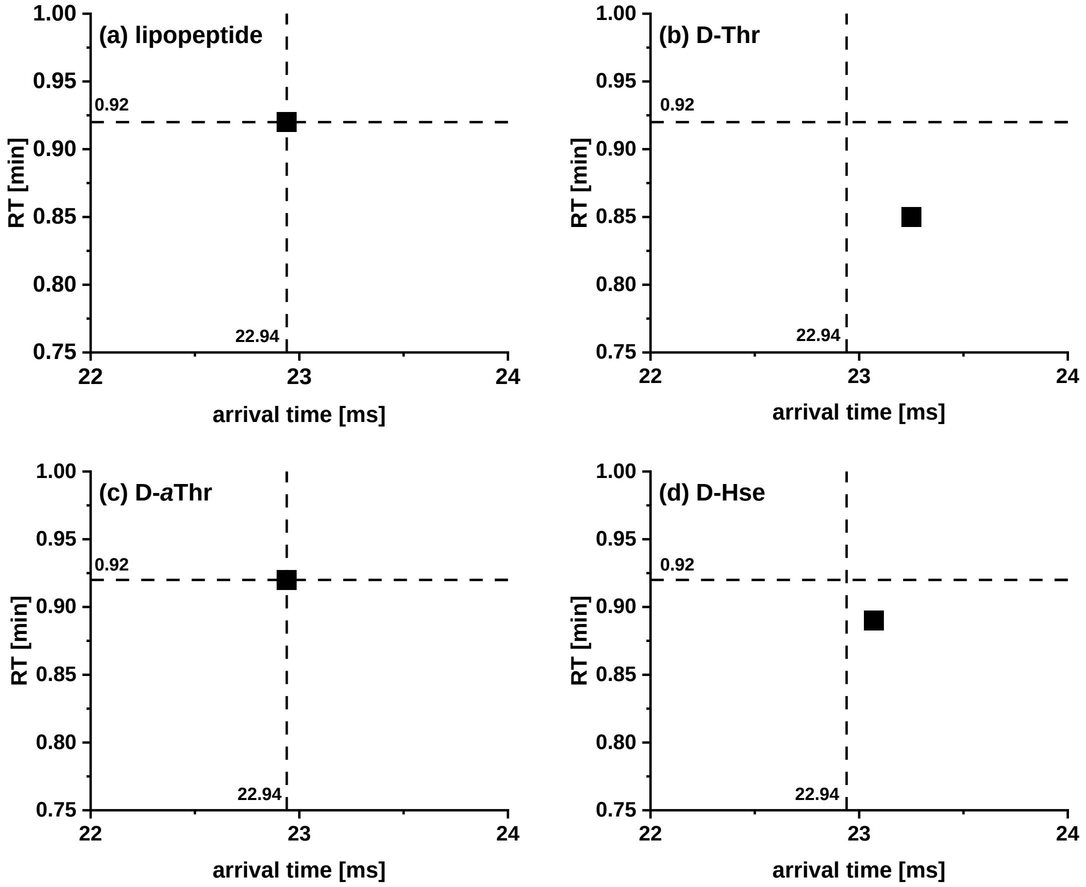


**Figure S22.** Retention time vs. arrival time plot for the (a) lipopeptide hydrolysate, (b) D-Thr, (c) D-*a*Thr and (d) D-Hse standards. Same conditions as in Figure 5.

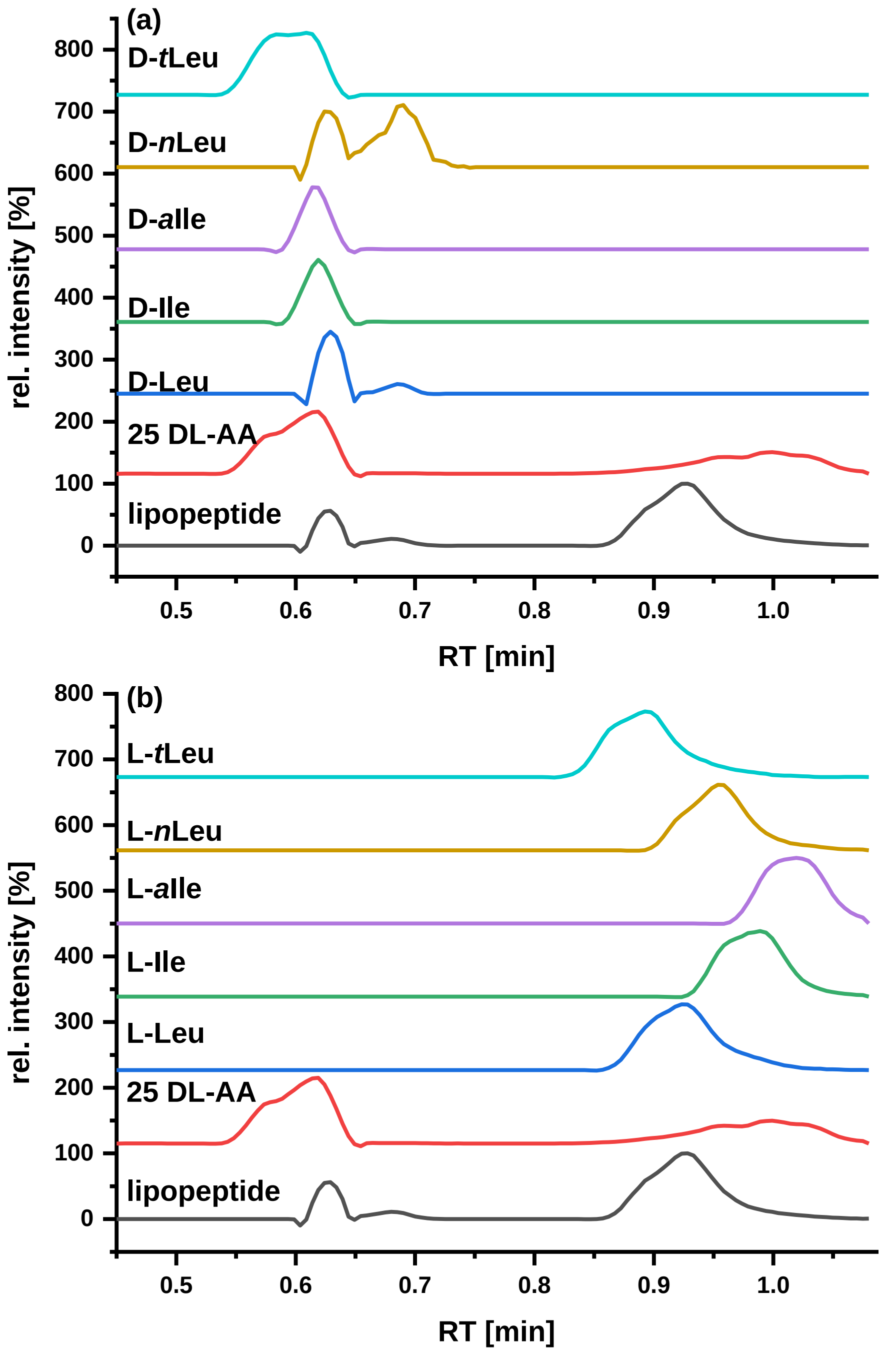


**Figure S23.** EICs of leucine isomers for the lipopeptide hydrolysate sample, 25 DL AQC-AA mix and single (a) D-AA and single (b) L-AA reference standard injections. Same experimental conditions as in Figure 5.

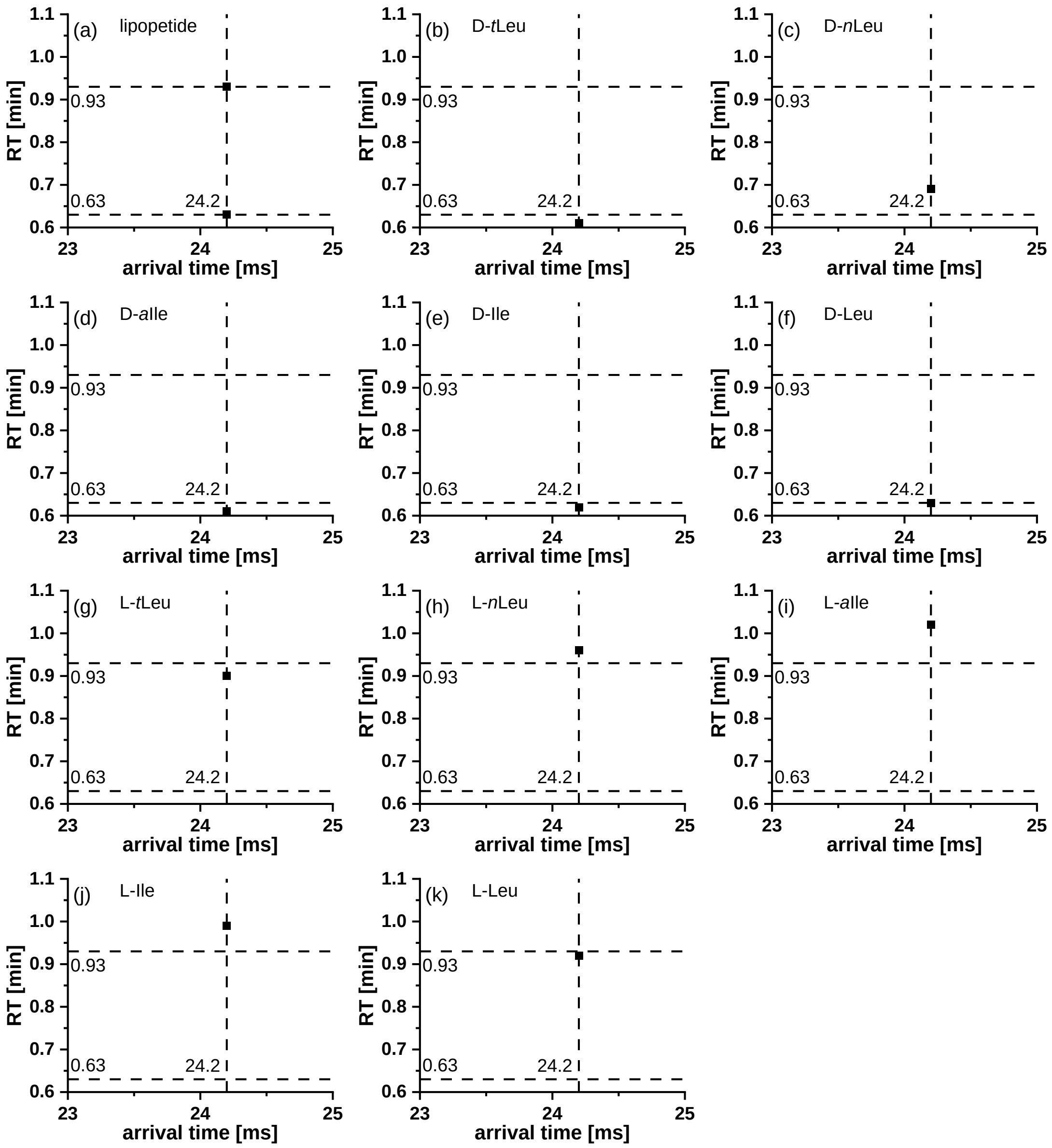


**Figure S24.** Retention time vs. drift time plot for the (a) lipopeptide hydrolysate, (b) D-*t*Leu, (c) D-*n*Leu, (d) D-*a*Ile, (e) D-Ile, (f) D-Leu, (g) L-*t*Leu, (h) L-*n*Leu, (i) L-*a*Ile, (j) L-Ile and (k) L-Leu. Same conditions as in Figure S23.

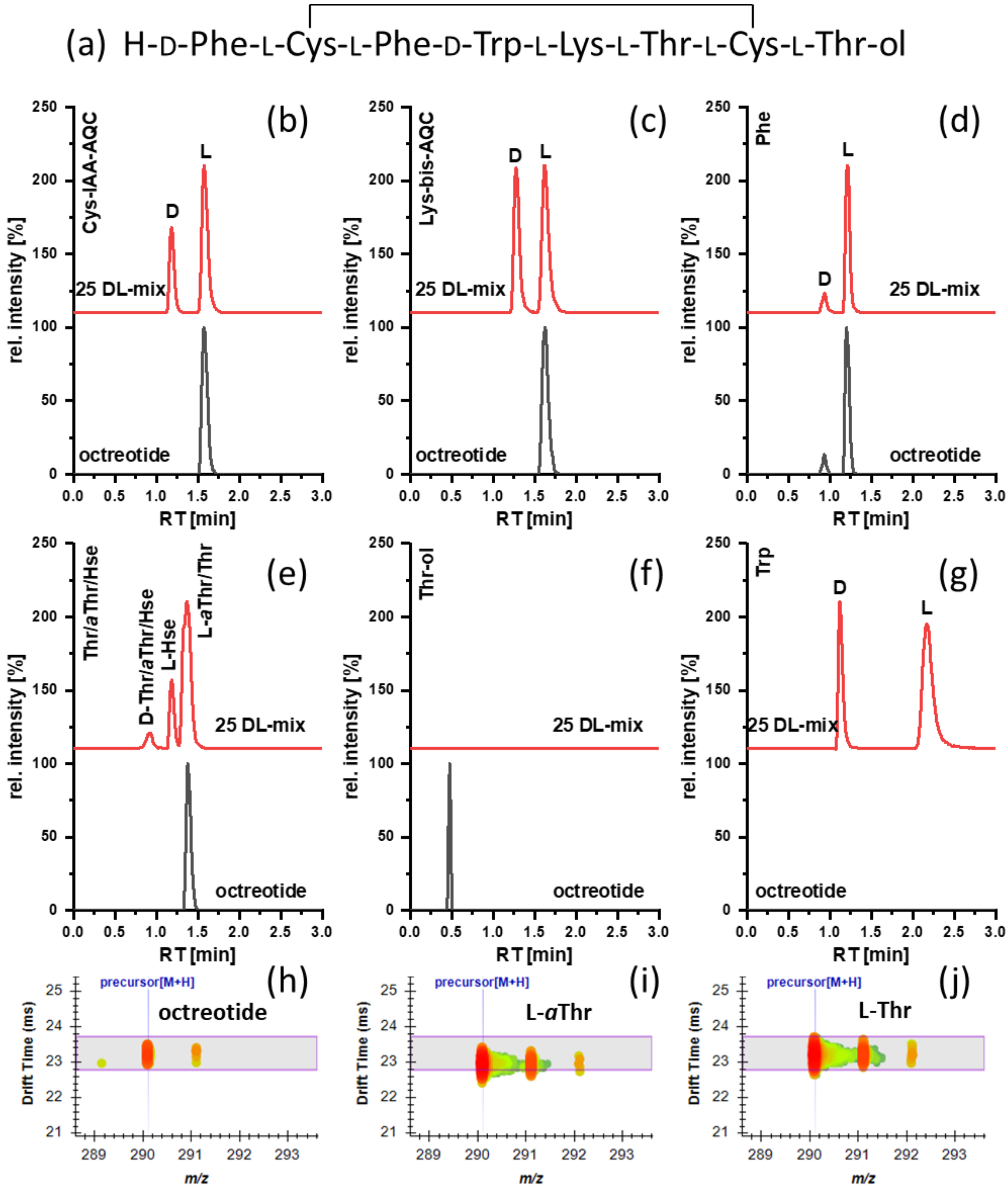


**Figure S25.** Enantioselective analysis of the peptide octreotide after its full hydrolysis**.** (a) Structure of octreotide. (b-g), EICs of the respective AQC-derivatized amino acids and drift time vs. *m/z* contour plots from threonine isomers of (h) octreotide hydrolysate, (i) L-*a*Thr and L-Thr single standards. Same experimental conditions as in Figure 5.

**Supplementary Note S6. Quantitative method performance**

Determination of limit of detection (LOD) was based on the slope *S'* and standard error of the y-intercept (σ) of calibration curve according to the ICH guideline Q2(R1):[5]

$$LOD = \frac{3.3\sigma}{S'} \qquad \text{(Eq. S1)}$$

The limit of quantification (LOQ) was calculated analogously:

$$LOQ = \frac{10\sigma}{S'} \qquad \text{(Eq. S2)}$$

**Table S23.** Calibration functions of the D-AQC-AAs including the linearity ($R^2$), the limit of detection (LOD), the limit of quantification (LOQ), the concentration range used for calibration, the accuracy and precision of a quality control sample and the applied quantification method. Either quantification based on light-to-heavy peak area ratios (L/H) or surrogate calibration with normalization of the peak areas by peak area of L-U-$^{13}C^{15}N$-Val (surrog.) was used depending on the signal intensity of the SIL-IS peak.

| AQC-AA[a] | slope | intercept | $R^2$ | LOD [µM] | LOQ [µM] | range [µM] | av. accuracy | precision | quant. Method |
|---|---|---|---|---|---|---|---|---|---|
| D-Ala | 3.69E-02 | -6.36E-05 | 0.999 | 0.14 | 0.42 | 0.2-10 | 90.6% | 16.1% | surrog. |
| D-Arg | 3.63E+00 | 3.17E-01 | 0.971 | 0.19 | 0.57 | 0.1-2 | 89.2% | 8.7% | L/H |
| D-Asn | 3.56E+00 | -5.91E-02 | 0.993 | 0.14 | 0.41 | 0.1-10 | 104.7% | 2.9% | L/H |
| D-Asp | 1.40E+00 | -3.02E-02 | 0.989 | 0.24 | 0.72 | 0.1-10 | 92.6% | 5.1% | L/H |
| D-Gln | 3.65E-01 | -9.83E-03 | 0.960 | 0.08 | 0.25 | 0.1-2 | 96.3% | 10.7% | surrog. |
| D-Glu | 2.21E+00 | -1.35E-02 | 0.976 | 0.19 | 0.56 | 0.1-2 | 104.4% | 13.1% | L/H |
| D-Gly | 9.78E-01 | 1.11E-02 | 0.994 | 0.12 | 0.35 | 0.1-10 | 94.6% | 3.5% | L/H |
| D-His | 4.33E-01 | 2.05E-02 | 0.983 | 0.17 | 0.51 | 0.1-2 | 90.7% | 12.8% | surrog. |
| D-Ile | 7.41E-01 | -1.32E-02 | 0.998 | 0.07 | 0.22 | 0.1-10 | 95.1% | 8.1% | L/H |
| D-Leu | 7.63E-01 | -2.26E-02 | 0.994 | 0.08 | 0.25 | 0.1-10 | 95.6% | 7.4% | L/H |
| D-Lys-bis-AQC | 6.10E-01 | 1.70E-03 | 0.931 | 0.19 | 0.56 | 0.1-2 | 93.8% | 9.3% | surrog. |
| D-Met | 2.55E-01 | -1.53E-02 | 0.975 | 0.08 | 0.25 | 0.1-2 | 95.8% | 8.1% | surrog. |
| D-Phe | 2.06E-01 | -7.60E-03 | 0.976 | 0.13 | 0.38 | 0.1-2 | 104.8% | 11.7% | surrog. |
| D-Pro | 2.81E+01 | -1.93E-01 | 0.985 | 0.15 | 0.46 | 0.1-2 | 92.8% | 5.1% | L/H |
| D-Ser | 1.87E-01 | -3.58E-03 | 0.993 | 0.09 | 0.28 | 0.1-2 | 91.1% | 3.9% | surrog. |
| D-Thr | 3.47E-02 | -4.54E-03 | 0.992 | 0.13 | 0.39 | 0.1-2 | 102.3% | 20.2% | surrog. |
| D-Trp | 9.35E-01 | -1.65E-02 | 0.950 | 0.10 | 0.30 | 0.1-2 | 96.3% | 9.7% | surrog. |
| D-Tyr | 5.05E+00 | -1.83E-01 | 0.993 | 0.14 | 0.44 | 0.1-2 | 100.1% | 9.1% | L/H |
| D-Val | 6.77E-01 | -1.15E-02 | 0.991 | 0.12 | 0.35 | 0.1-2 | 94.3% | 6.5% | L/H |

[a] Quantification of Cys was not possible as some sample preparation problems occurred and alkylation seems to be incomplete

**Table S24.** Calibration functions of the L-AQC-AAs including the linearity ($R^2$), the limit of detection (LOD), the limit of quantification (LOQ), the concentration range used for calibration, the accuracy and precision of a quality control sample and the applied quantification method. Either quantification based on light-to-heavy peak area ratios (L/H), surrogate calibration with normalization of the peak areas by peak area of L-U-$^{13}C^{15}N$-Val (surrog.) or calibration by normalization by total ion current (TIC) was used depending on most appropriate conditions.

| AQC-AA[a] | slope | intercept | $R^2$ | LOD [µM] | LOQ [µM] | range [µM] | av. accuracy | precision | quant. Method |
|---|---|---|---|---|---|---|---|---|---|
| L-Ala | 3.31E-01 | 2.34E-02 | 0.985 | 0.12 | 0.35 | 0.1-2 | 88.0% | 8.9% | surrog. |
| L-Arg | 1.06E+00 | 2.97E-02 | 0.988 | 0.08 | 0.24 | 0.1-2 | 92.3% | 6.5% | surrog. |
| L-Asn | 1.50E+00 | 1.31E-01 | 0.991 | 0.09 | 0.27 | 0.1-2 | 99.1% | 9.2% | L/H |
| L-Asp | 2.65E-01 | 1.90E-02 | 0.976 | 0.15 | 0.45 | 0.1-2 | 87.6% | 10.2% | surrog. |
| L-Gln | 1.27E+00 | 4.68E-02 | 0.959 | 0.18 | 0.54 | 0.1-2 | 90.6% | 12.2% | L/H |
| L-Glu | 1.18E+00 | 1.38E-01 | 0.959 | 0.20 | 0.59 | 0.1-2 | 102.3% | 8.0% | L/H |
| L-Gly | 9.78E-01 | 1.08E-02 | 0.994 | 0.11 | 0.35 | 0.1-10 | 94.5% | 3.3% | L/H |
| L-His | 5.08E-01 | 9.86E-03 | 0.993 | 0.13 | 0.39 | 0.1-2 | 83.2% | 9.4% | surrog. |
| L-Ile | 5.37E-01 | 3.04E-02 | 0.993 | 0.07 | 0.23 | 0.1-2 | 92.9% | 5.7% | L/H |
| L-Leu | 5.37E-01 | 3.02E-02 | 0.992 | 0.08 | 0.24 | 0.1-2 | 92.9% | 5.0% | L/H |
| L-Lys-bis-AQC | 3.63E+05 | 2.72E+04 | 0.950 | 0.16 | 0.48 | 0.1-2 | 92.0% | 14.8% | TIC |
| L-Met | 8.58E-01 | 4.52E-02 | 0.978 | 0.07 | 0.22 | 0.1-2 | 93.1% | 5.4% | surrog. |
| L-Phe | 1.44E+00 | 7.85E-02 | 0.996 | 0.05 | 0.16 | 0.1-2 | 96.9% | 5.1% | L/H |
| L-Pro | 2.97E+00 | 2.93E-02 | 0.987 | 0.17 | 0.51 | 0.1-2 | 98.5% | 11.2% | L/H |
| L-Ser | 1.99E+00 | 1.03E-01 | 0.967 | 0.15 | 0.47 | 0.1-2 | 91.5% | 8.6% | L/H |
| L-Thr | 2.24E+00 | 9.10E-02 | 0.994 | 0.08 | 0.23 | 0.1-2 | 91.4% | 3.5% | L/H |
| L-Trp | 1.45E+00 | 4.08E-02 | 0.991 | 0.07 | 0.21 | 0.1-2 | 97.0% | 3.9% | surrog. |
| L-Tyr | 2.91E+00 | 6.80E-02 | 0.994 | 0.08 | 0.24 | 0.1-10 | 95.7% | 2.1% | L/H |
| L-Val | 4.70E-01 | 2.15E-02 | 0.994 | 0.16 | 0.49 | 0.1-2 | 94.4% | 8.0% | L/H |

[a] Quantification of Cys was not possible as some sample preparation problems occurred and alkylation seems to be incomplete

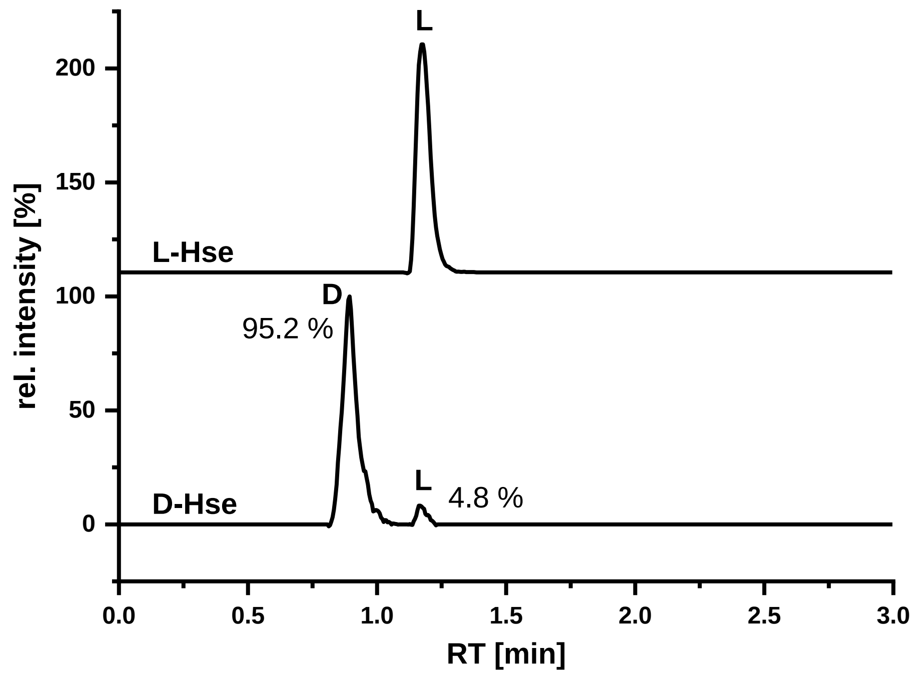


**Figure S26.** Extracted ion chromatograms (EICs) of AQC-derivatized L-Hse and D-Hse standards. The D-Hse standard has an L-Hse impurity peak with 4.8 % of the total peak area, documenting the applicability of the method for amino acids with extreme enantiomer ratio.

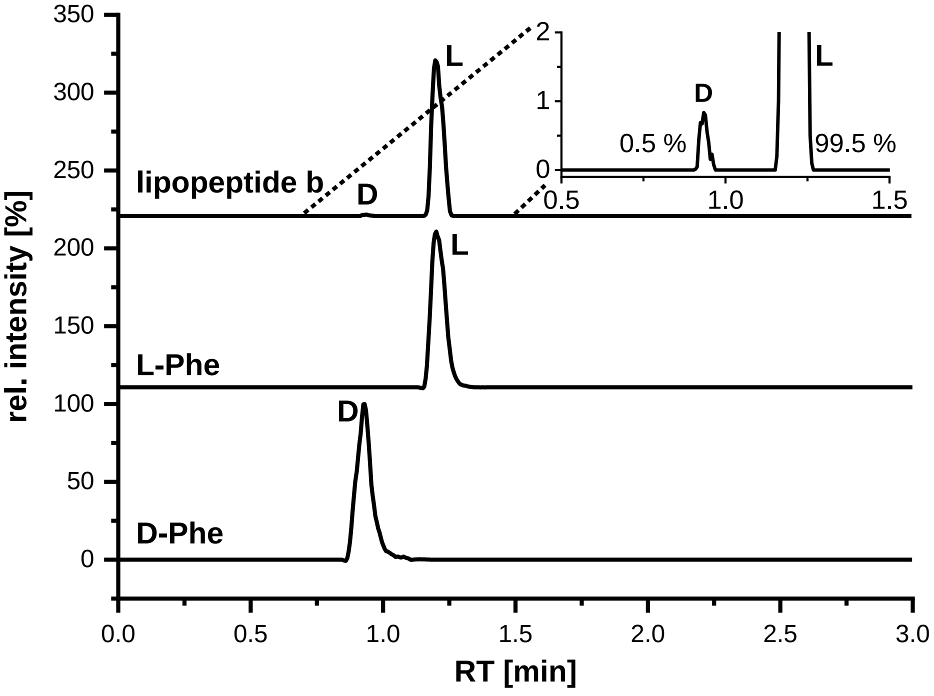


**Figure S27.** Extracted ion chromatograms (EICs) of single AQC-derivatized L-Phe and D-Phe standards and an additional lipopeptide sample b (after hydrolysis and AQC-derivatization) which has a small D-Phe peak (0.5 % peak area) while the majority is L-Phe (99.5 %), documenting the applicability of the method for amino acids with extreme enantiomer ratio.

.

**Supplementary References**

(1) Schmitt, K.; Woiwode, U.; Kohout, M.; Zhang, T.; Lindner, W.; Lämmerhofer, M. Comparison of small size fully porous particles and superficially porous particles of chiral anion-exchange type stationary phases in ultra-high performance liquid chromatography: effect of particle and pore size on chromatographic efficiency and kinetic performance. *J. Chromatogr. A* **2018**, *1569*, 149-159. DOI: https://doi.org/10.1016/j.chroma.2018.07.056.

(2) Geibel, C.; Dittrich, K.; Woiwode, U.; Kohout, M.; Zhang, T.; Lindner, W.; Lämmerhofer, M. Evaluation of superficially porous particle based zwitterionic chiral ion exchangers against fully porous particle benchmarks for enantioselective ultra-high performance liquid chromatography. *J. Chromatogr. A* **2019**, *1603*, 130-140. DOI: https://doi.org/10.1016/j.chroma.2019.06.026.

(3) Butler, K. E.; Dodds, J. N.; Flick, T.; Campuzano, I. D. G.; Baker, E. S. High-Resolution Demultiplexing (HRdm) Ion Mobility Spectrometry–Mass Spectrometry for Aspartic and Isoaspartic Acid Determination and Screening. *Anal. Chem.* **2022**, *94* (16), 6191-6199. DOI: 10.1021/acs.analchem.1c05533.

(4) May, J. C.; Knochenmuss, R.; Fjeldsted, J. C.; McLean, J. A. Resolution of Isomeric Mixtures in Ion Mobility Using a Combined Demultiplexing and Peak Deconvolution Technique. *Anal. Chem.* **2020**, *92* (14), 9482-9492. DOI: 10.1021/acs.analchem.9b05718.

(5) ICH. ICH Harmonised Tripartite Guideline - Validation of Analytical Procedures: Text and Methodology Q2(R1). 2005; pp 1-13.
